# Transferring Micellar Changes to Bulk Properties via Tunable Self-Assembly and Hierarchical Ordering

**DOI:** 10.1021/acsnano.2c06898

**Published:** 2022-11-28

**Authors:** Lisa Thomson, Daniel McDowall, Libby Marshall, Olivia Marshall, Henry Ng, W. Joseph A. Homer, Dipankar Ghosh, Wanli Liu, Adam M. Squires, Eirini Theodosiou, Paul D. Topham, Louise C. Serpell, Robert J. Poole, Annela Seddon, Dave J. Adams

**Affiliations:** † School of Chemistry, University of Glasgow, Glasgow G12 8QQ, U.K.; ‡ School of Engineering, University of Liverpool, Liverpool L69 3GH, U.K.; § Aston Institute of Materials Research, Aston University, Birmingham B4 7ET, U.K.; ∥ Department of Chemistry, University of Bath, Bath BA2 7AY, U.K.; ⊥ Sussex Neuroscience, School of Life Sciences, University of Sussex, Falmer BN1 9QG, U.K.; # School of Physics, HH Wills Physics Laboratory, University of Bristol, Tyndall Avenue, Bristol BS8 1TL, U.K.

**Keywords:** micelle, liquid crystal, dipeptide, polyelectrolyte, SAXS, WAXS

## Abstract

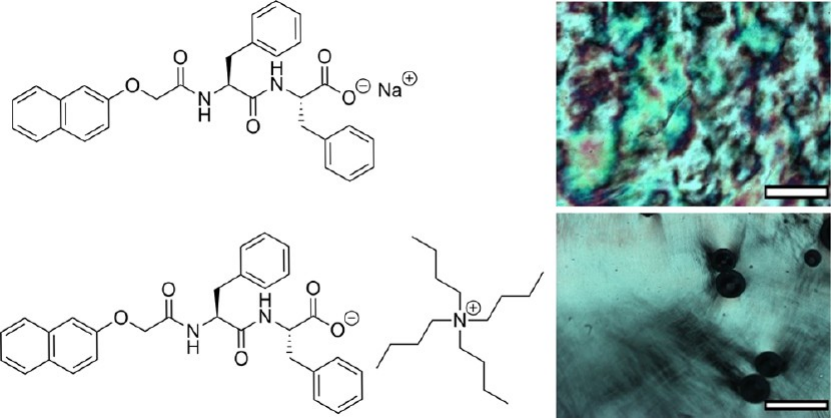

Hierarchical self-assembly is an effective means of preparing useful materials. However, control over assembly across length scales is a difficult challenge, often confounded by the perceived need to redesign the molecular building blocks when new material properties are needed. Here, we show that we can treat a simple dipeptide building block as a polyelectrolyte and use polymer physics approaches to explain the self-assembly over a wide concentration range. This allows us to determine how entangled the system is and therefore how it might be best processed, enabling us to prepare interesting analogues to threads and webs, as well as films that lose order on heating and “noodles” which change dimensions on heating, showing that we can transfer micellar-level changes to bulk properties all from a single building block.

## Introduction

Hierarchical assembly is a common aspect in natural materials that is often hard to control and direct in synthetic analogues. The ability to make tunable soft materials with controlled order over a wide range of length scales is a major challenge, but success would open applications in a range of fields including soft machines, soft robotics, and artificial muscles.^1^ Numerous classes of self-assembling building blocks exist which, due to the characteristics of the individual building block, give rise to a rich polymorphism.^2^ However, we are often left in a position where a single molecule is perceived to self-assemble into a single aggregated structure, which implies for each new structure that a new molecule is required. Furthermore, the ability to control the entire hierarchical assembly process from the molecular scale to the mesoscale is lacking.

Functionalized peptides can be used to form useful materials.^3−6^ The more hydrophobic dipeptides often form persistent micellar aggregates at low volume fractions in water.^7^ These subsequently can form gels on change of pH or addition of salts.^7,8^ There is a tension with this kind of material in terms of understanding and describing behavior with a significant focus on these materials as peptide-based. Many reports highlight (for example) the peptides packing into secondary structures like beta-sheets.^5,9,10^ A subset of peptide-based gelators has been described in terms of micellar behavior,^11−13^ but it is common to focus on low concentrations where gels are easily formed; phase diagrams are rare. The structures formed can often be persistent wormlike micelles, and hence polyelectrolyte behavior is expected. There are significant similarities between ionic wormlike micelles and polyelectrolytes, and analogies have been drawn in many cases.^14,15^ Indeed, wormlike micellar fluids are often referred to as “living polymers”.^16^

Ultimately, however, to design useful materials, we need to understand and control molecular packing and translate properties across length scales. There are examples where useful materials have been made via hierarchical peptide assembly,^17−20^ but a key question is whether we can invert the approach; rather than observing a specific behavior and then using the outcome, can we instead determine what properties would be required for a specific material and then rationally design a self-assembling system to exhibit these?

Here, we demonstrate that a single small molecule can be used to create a hierarchy of self-assembled structures that can be understood using conventional polymer-based theories, allowing us to understand materials with hierarchical order and control across multiple length scales. We use small angle scattering data to predict the macroscopic properties of the material and how it will behave when it is processed.

## Results and Discussion

**Figure 1:**
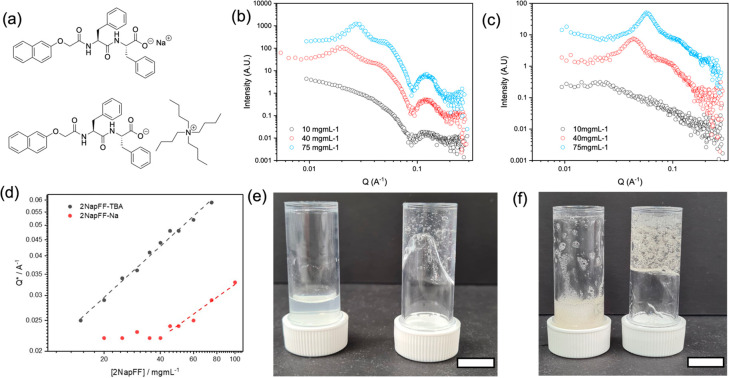
(a) Chemical structure of 2NapFF-Na and 2NapFF-TBA; (b) example 1D SAXS plots for 2NapFF-Na at 10 (black), 40 (red), and 75 mg/mL (blue); (c) example 1D SAXS plots for 2NapFF-TBA at 10 (black), 40 (red), and 75 mg/mL (blue); for (b) and (c), the data are offset on the *y*-axis arbitrarily for clarity; (d) concentration vs Q* peak position with fit to polyelectrolyte model for 2NapFF-Na (red) and 2NapFF-TBA (black). The lines show a theoretical c^0.5^ scaling; photographs of (e) 10 mg/mL and (f) 75 mg/mL 2NapFF-Na (left) and 2NapFF-TBA (right). The scale bar for (e) and (f) represents 1 cm. The bubbles are due to the stirring protocol just prior to photographing.

Solutions of 2NapFF (Figure 1a) were prepared at pH 10.5. Changing the counterion of the base used to deprotonate the 2NapFF at low concentration and high pH leads to different self-assembled structures in solution.^21^ Here we focus on two examples, the sodium salt and the tetrabutylammonium salt (2NapFF-Na and 2NapFF-TBA, respectively). These two salts were chosen on the basis of our work at low concentration where there were found to be significant differences in the self-assembled structure.^21^ Previous work on such systems has focused on concentrations of 10 mg/mL or less. Here, solutions were prepared at concentrations of 5–100 mg/mL (5–75 mg/mL for 2NapFF-TBA). It is necessary to carefully control the shear history during formulation to prepare reproducible samples (Section 1, Supporting Information).

1D SAXS data were collected on solutions of increasing concentrations of 2NapFF-Na and 2NapFF-TBA. The data for the 2NapFF-Na can be fitted to a flexible cylinder form factor with a radius of ∼4.2 nm (Figure 1b, intensity offset for clarity; Figure S7 and fitting parameters in Table S1, Supporting Information). The exceptions to this are the highest concentrations studied (60, 75, 100 mg/mL), where the flexibility has been lost and the fit is now to a rigid cylinder form factor. The length of the cylinders is outside of the fittable Q range (i.e., > 100 nm). These data demonstrate that the overall structure and dimensions of the cylinder are unchanged with concentration. The structures are actually hollow tubes;^7^ however, using SAXS, the contrast from the hollow core cannot be detected and so a fit to a flexible cylinder is most appropriate. Examination of the scale parameter in the model fit, indicating the volume fraction of scatterers in solution, shows that this value increases with increasing concentration, and as such, there are a greater number of cylinders formed. The reduced χ^2^ value of the fit increases with increasing concentration as the samples can no longer be considered to be dilute.^22^

A similar data series was collected for 2NapFF-TBA (Figure 1c, Figure S8, and Table S2). The underlying form factor fit to the data remains the same at all concentrations as a flexible cylinder with a radius of 1.5 nm. However, the Kuhn length becomes unfittable at concentrations above 30 mg/mL, indicating that the sample is now behaving as rigid cylinders. The scale factor does not increase drastically with increasing concentration, showing that the addition of more 2NapFF molecules to the system does not simply result in more cylinders in solution but rather adds length to the existing cylinders and promotes micellar branching.^23^

As the concentration increases from the dilute to what might be considered the semidilute regime^24^ in both samples, a peak (Q*) appears at low *Q*. This peak represents the correlation length of the system, ξ, sometimes called the screening length, and is the scale at which for charged systems all charge repulsions are screened. de Gennes showed for polyelectrolytes that the concentration dependence of a correlation length in solution can be rationalized simply by a scaling argument and that for a fully extended polyelectrolyte in the absence of salt ξ ∼ c^1/2^.^24,25^ Polyelectrolytes in the absence of salt will show a peak at *Q* = 2πξ^–1^; the peak is due to large osmotic pressures in polyelectrolyte solutions which do not allow correlation volumes to overlap as they might, for example, in a good solvent. A log–log plot of ξ (Q*) against concentration for 2NapFF-Na has a gradient of *c*^0.46^, close to the value expected by the scaling law for a polyelectrolyte with no salt (Figure 1d). Therefore, each wormlike micelle can be considered as a polyelectrolyte chain. At pH 10.5, each wormlike micelle is negatively charged. As such, a polyelectrolyte model fits our physical understanding of the system. At low concentrations (<20 mg/mL), typically used to prepare gels from such materials, the peak in Q* is absent.

The data for 2NapFF-TBA show a similar peak, which shows a gradient of 0.53, again consistent with a polyelectrolyte-like system. However, unlike 2NapFF-Na, the peak is evident at lower concentrations (15 mg/mL) indicating that there is a greater interaction between the flexible cylinders in solution at a lower concentration than for 2NapFF-Na. Plotting the change in correlation length (Figure S12) shows that 2NapFF-TBA has a more pronounced drop in correlation length with increasing concentration and that at 75 mg/mL the correlation length reached is far smaller than that seen for the same concentration of 2NapFF-Na (10 nm versus 22 nm respectively). Thus, the counterion not only controls the micellar structure but also influences the aggregate interactions via modification of the micellar dimensions.

**Figure 2:**
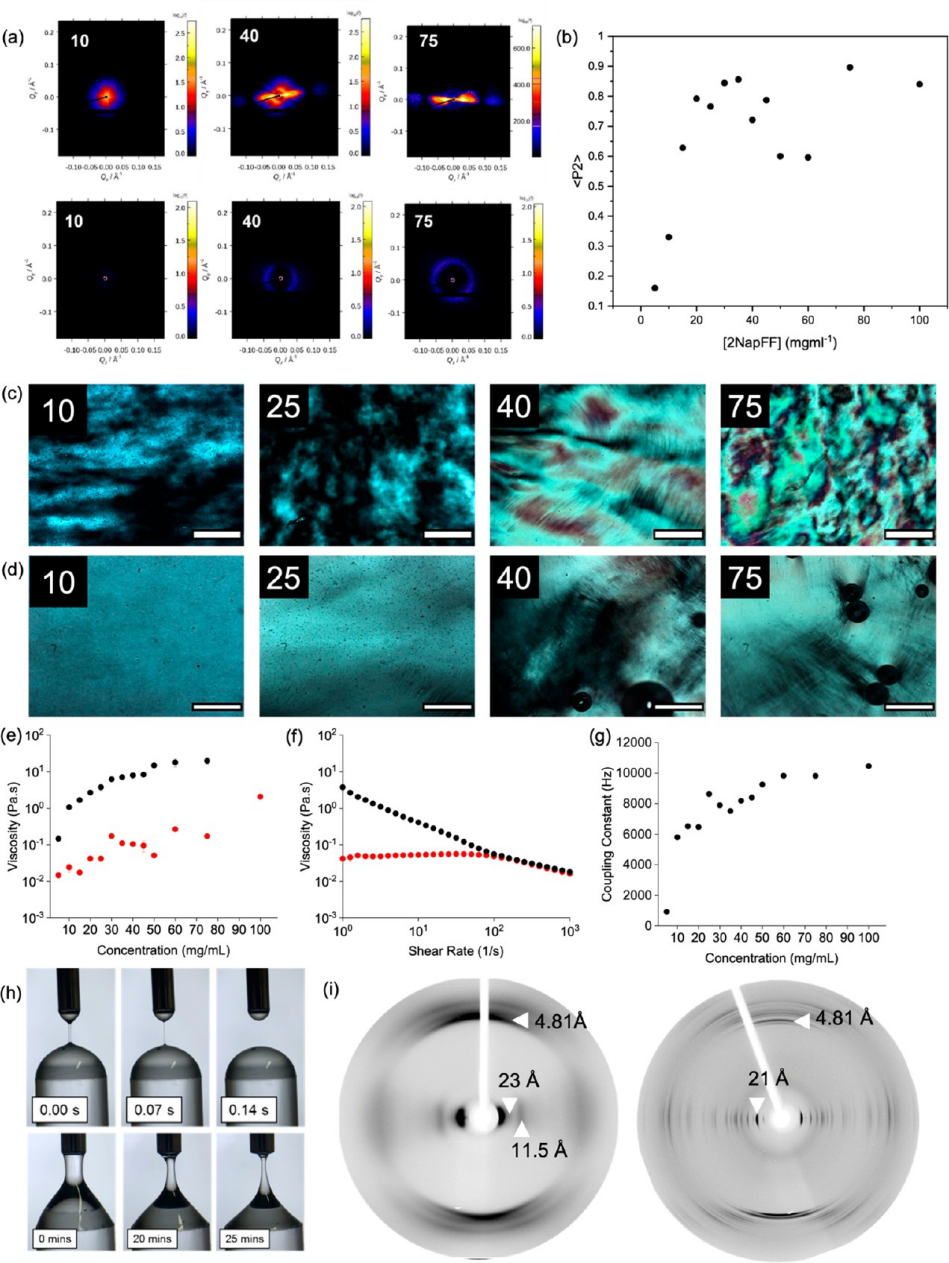
(a) Example 2D SAXS patterns for (top) 2NapFF-Na and (bottom) 2NapFF-TBA at 10, 40, and 75 mg/mL. (b) ⟨P2⟩ data for 2NapFF-Na; polarized optical microscopy (POM) images of (c) 2NapFF-Na and (d) 2NapFF-TBA at concentrations of 10, 25, 40, and 75 mg/mL (concentration as inset in top left of each image) taken at 5× magnification. Scale bars represent 500 μm. (e) Comparison of viscosity at a shear rate of 1 s^–1^ for 2NapFF-Na (red) and 2NapFF-TBA (black). (f) Example viscosity data for 2NapFF-Na (red) and 2NapFF-TBA (black) at 25 mg/mL. (g) Summary of ^23^Na NMR for 2NapFF-Na. (h) Dripping-onto-substrate images for 25 mg/mL 2NapFF-Na (top) and 2NapFF-TBA (bottom). (i) fXRD for (left) 2NapFF-Na and (right) 2NapFF-TBA.

The extended polyelectrolyte-like conformation of these interacting chains in the semidilute regime has implications for their alignment. As the concentration of 2NapFF-Na increases, the samples display an alignment parallel to the long axis of the capillary in which they are measured in the 2D SAXS data (Figure 2a). The degree of alignment can be quantified by fitting the ⟨P2⟩ order parameter (Figure 2b and Figure S11), and the samples show the distinctive Schlieren texture of a nematic liquid crystal (Figure 2c and Figure S15). There is an isotropic to nematic transition at a 2NapFF-Na concentration of approximately 20 mg/mL, corresponding to the concentration at which the correlation length peak becomes visible in the 1D pattern.

While the onset of the Q* peak can be linked to the ordering of the cylinders in 2NapFF-Na, the situation is different for 2NapFF-TBA. The 2D SAXS patterns show very little ordering, and this order is not linearly related to concentration (Figure 2a and Figure S10). Furthermore, a Q* peak appears in sample concentrations where no alignment is observed. This shows that the cylinders in 2NapFF-TBA are interacting at lower concentration but are unable to align in the manner seen for the Na analogue, again indicative of micellar branching.^15,23^ To test this hypothesis, we examined low-shear viscosity as a function of concentration which can indicate where changes in structure occur. At a shear rate of 1 s^–1^ (Figure 2e, example data Figure 2f, full data in Figure S17 and S18), the viscosity of the solutions mirrors the plot of ⟨P2⟩.

The wormlike micelles formed by 2NapFF-Na can be aligned in a magnetic field.^26^ The degree of alignment is again concentration dependent (Figure 2g) and correlates with the ⟨P2⟩ data. Hence, by simply changing the concentration of 2NapFF-Na, we have control over the ordering of the 2NapFF wormlike micelles. In comparison, the branched micelles formed by 2NapFF-TBA cannot be aligned in a magnetic field (Figures S24–26).

Changing the counterion used translates directly into differences in solution properties. The 2NapFF-TBA solutions are significantly more viscous than the 2NapFF-Na solutions (Figure 2e). This increase in viscosity could be partly the reason for the inability to align 2NapFF-TBA in a magnetic field. This, however, cannot be the only factor as the viscosity of 2NapFF-TBA at 5 mg/mL is similar to that of 2NapFF-Na at concentrations of 30–50 mg/mL (Figure 2e), which can be aligned. The extensional viscosities are very different; the extensional relaxation time (λ_E_) was studied at 25 mg/mL using the dripping-onto-substrate technique.^27^ For 2NapFF-TBA, λ_E_ is too large to measure (the filament dries out before breaking) whereas for 2NapFF-Na λ_E_ = 14.6 ± 6.5 ms (Figure 2h).

WAXS data show that the degree of order in each system depends on the counterion used (Figure S13 and S14). For 2NapFF-Na, peaks in the WAXS appear above 10 mg/mL, coinciding with the onset of alignment. The peaks in the WAXS arise from each cylinder lining up in the same direction as the cylinder next to it, and this “confinement” leads to ordering in the packing. For 2NapFF-TBA, the WAXS only shows a single peak in this region (Figure S14) corresponding to the radius of the much thinner 2NapFF-TBA cylinder. Air-dried aligned fibers were prepared, and fiber X-ray diffraction (fXRD) data were collected (these are not dehydrated and retain some water). These data (Figure 2i) show that on drying to form the stalk, the 2NapFF-Na shows a classic fiber diffraction pattern consistent with flexible fibers aligned along the sample axis. The 2NapFF-TBA diffraction pattern is more consistent with randomly oriented crystallites which give rise to much sharper reflections. In both cases, organization along the fiber axis appears similar while the packing and lateral organization are different. We can therefore control the degree of order within the system both in the solution and dry state by choice of counterion. There are analogies here with controlling the degree of order in DNA stalks with humidity.^28^

**Figure 3:**
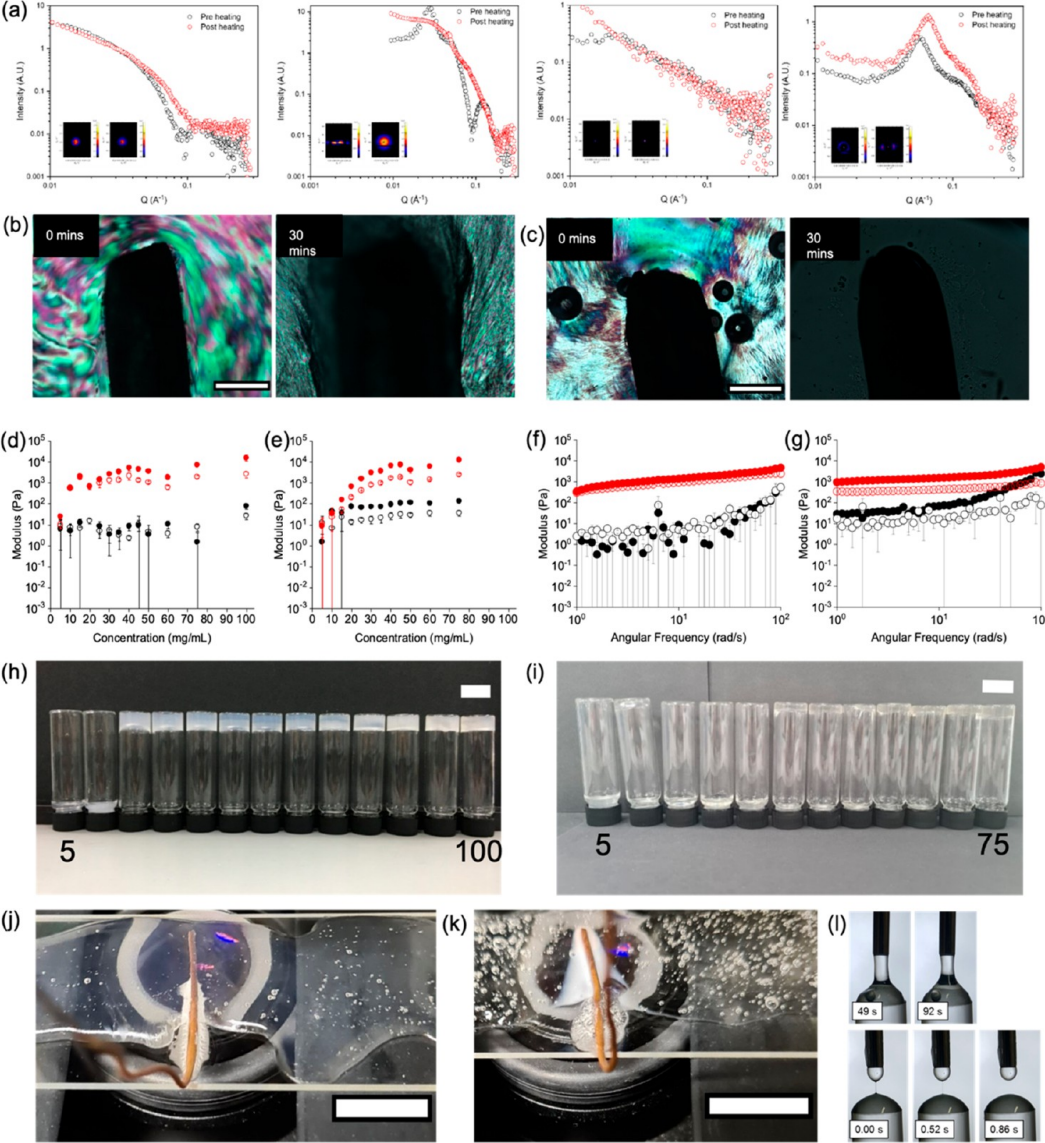
(a) SAXS plots for heat–cool cycles—from left to right 2NapFF-Na at 10 and 75 mg/mL and 2NapFF-TBA at 10 and 75 mg/mL. The black data shows before heating and red data after a heat–cool cycle. Inset shows the 2D plots. Heating (b) 2NapFF-Na and (c) 2NapFF-TBA using a copper wire. POM images taken before heating (0 min) and after 30 min of continuous heating through the wire. Scale bars represent 500 μm. (d) and (e) show a summary of frequency sweep data collected for 2NapFF-Na and 2NapFF-TBA, respectively, measured at room temperature (black) and at room temperature 2 h after a heat–cool cycle (red). (f) and (g) show example frequency data for 2NapFF-Na and 2NapFF-TBA, respectively, at 25 mg/mL measured at room temperature (black) and at room temperature 2 h after a heat–cool cycle (red). For (d)–(g), *G*′ is represented by filled circles and *G*′′ by empty circles. Error bars represent the standard deviation between measurements, which were carried out in triplicate. (h) and (i) show photographs of 2NapFF-Na and 2NapFF-TBA, respectively, 1 day after heat–cool. Scale bars represent 2 cm. (j) and (k) show photographs of 2NapFF-Na and 2NapFF-TBA, respectively, at a concentration of 75 mg/mL taken at 15 min of heating with a copper wire. 2NapFF-TBA becomes cloudy compared to 2NapFF-Na which shows no change. Superglue is seen in both images at the bottom of the slide which was used to secure the copper wire. (l) Dripping-onto-substrate images for 25 mg/mL 2NapFF-Na (top) and 2NapFF-TBA (bottom) after a heat–cool cycle.

Subjecting the micellar solutions at low concentrations to a heat–cool cycle can have a profound effect on their structure.^8^ Heat–cool cycles were performed on solutions of 2NapFF-Na at a concentration of 10, 40, and 75 mg/mL. In all cases, the radii of the cylinders decrease, and the Kuhn length is reduced (Figure 3a). This is extremely marked in the highest concentration samples which now fit to a flexible cylinder form factor, showing that the rigidity has been lost (Table S3). The samples post heating also show a reduced intensity correlation peak, and this peak has shifted to higher *Q*, and thus a lower value of ξ, reflecting their reduced radii. The orientation in the 2D scattering pattern is lost, and the samples are now isotropic (Figure 3a). The lack of order can also be seen in the polarized microscopy (Figure 3b,c and Figures S31 and S32) and NMR experiments (Figures S19–S26) where a change in the texture and a loss of the splitting of the D_2_O peak are seen, respectively. Before heat–cool, 2NapFF-Na samples exhibit a loss modulus (G′′) less than the storage modulus (*G*′) with the exception of 2NapFF-Na at 100 mg/mL (Figure 3d and Figures S33–S35). At concentrations of 25 mg/mL and greater, *G*′ is higher than *G*′′ in small amplitude oscillatory shear after a heat–cool cycle, although tan δ > 0.16.

Before the heat–cool cycle, the 2NapFF-TBA samples are visibly much more viscous compared to 2NapFF-Na (Figure S30). Without heat–cool, *G*′ is greater than G′′ for 2NapFF-TBA concentrations of 15 mg/mL and higher (Figure 3e and Figures S36–S38). When 2NapFF-TBA samples are heated and cooled for 2 h, *G*′ and G′′ increase significantly (*G*′ is larger than *G*′′ for 2NapFF-TBA concentrations of 15 mg/mL and greater) but again are not true gels with tan δ > 0.2 for all concentrations. Example frequency data for both 2NapFF-Na and 2NapFF-TBA are shown in Figures 3f,g. The 2NapFF-TBA samples were less stable to inversion after a heat–cool cycle than the 2NapFF-Na samples (Figures S39 and S40).

When 2NapFF-TBA samples are removed from the oven, the viscosity is significantly lower as compared to prior to heating. Monitoring over the heat–cool cycle, the viscosity drops approximately 4 orders of magnitude when heating to 60 °C; in comparison, the viscosity of the 2NapFF-Na solutions do not decrease when heating (Figure S41). Again, DoS was used to study the λ_E_ of the fluids and the different counterion behavior is reversed as compared to before heating. 2NapFF-Na was too viscous to measure, whereas 2NapFF-TBA was measured to be λ_E_ = 346 ± 110 ms (Figure 3l). In addition to this, the 2NapFF-TBA also shows visible changes when heating whereas 2NapFF-Na does not (Figures 3j,k). At higher concentrations (>25 mg/mL), phase separation occurs in the 2NapFF-TBA samples on heating, with the samples re-homogenizing spontaneously on cooling (Figure S42). Such lower critical solution temperature (LCST) behavior has been shown for other micellar and discotic systems,^29−31^ but it is interesting that only the 2NapFF-TBA shows this effect. The reason as to why this is observed with 2NapFF-TBA and not 2NapFF-Na is presumably due to the differences in micellar structures present, but more work is needed to understand this fully. The use of a copper wire to heat the solutions (Figure 3j,k) allows us to induce gradients of heating within the sample and hence regions above the LCST and regions below it, shown by the difference in color of the solution in Figure 3k.

Hence, changing the counterion results in differences in molecular packing, which translates directly into differences in properties such as the concentration dependence of order, distance between micelles, and alignment of the primary structures. These differences result in changes in flow behavior, extensional viscosity, and the formation of liquid crystalline phases. Understanding this allows us to rationally determine which systems should be used for different applications. To exemplify this, we have chosen a small number of specific examples.

**Figure 4:**
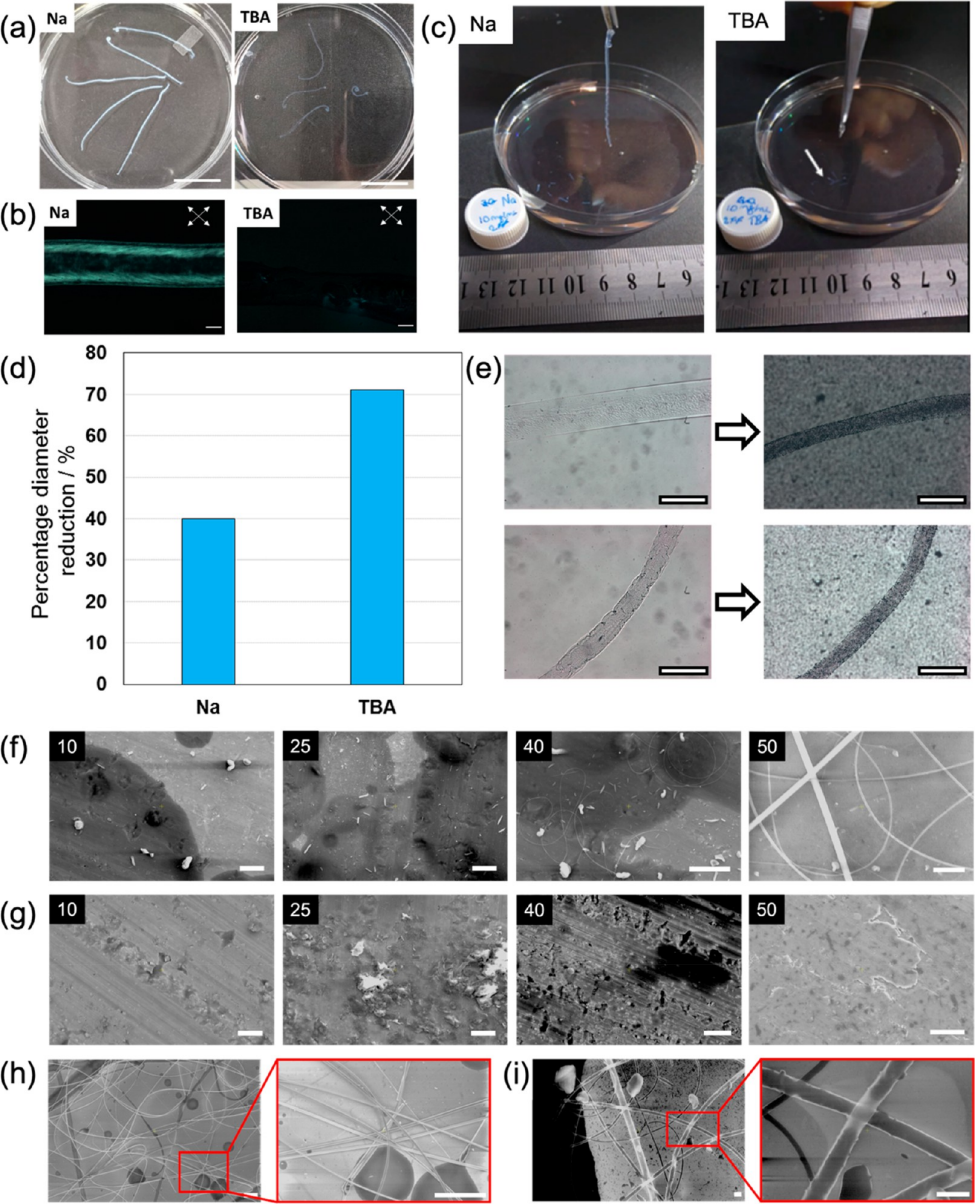
(a) Gel noodles made with both counterions (at 25 mg/mL 2NapFF); (b) cross-polarized optical microscope images (scale bars represent 0.2 mm); (c) photographs showing differences in mechanical strength of the noodle. The noodles were formed in a dish and then picked up at one end using tweezers and lifted out of the calcium bath. The 2NapFF-Na noodles are robust enough to be picked up. The 2NapFF-TBA noodles break when lifting, even after multiple attempts. The white arrow is added to guide the eye to broken sections of noodles. (d) The diameters of the noodles shrink on a heat–cool cycle, with the 2NapFF-Na shrinking more than the 2NapFF-TBA noodles; (e) photographs showing example noodles before and after a heat–cool cycle. Top shows a 2NapFF-Na noodle and bottom shows a 2NapFF-TBA noodle; (f) and (g) SEM images of substrates following electrospinning of 2NapFF-Na and 2NapFF-TBA solutions from 10 to 50 mg/mL, respectively; (h) SEM images of microscale fibers formed by electrospinning of 50 mg/mL of 2NapFF-Na (scale bar represents 5 μm); (i) SEM images of nanoscale fibers formed by electrospinning of the same solution (scale bar represents 500 nm).

Gel noodles can often be formed from solutions such as these used here by extrusion into a bath of a gelling agent, typically a divalent salt.^32−34^ Gel noodles can be prepared from a range of molecules, but there are limited design rules. Such noodles have applications in conductive materials^35,36^ and for directing cell growth,^37^ for example. Here, we find noodles can be formed from both the 2NapFF-Na and 2NapFF-TBA (Figure 4a). However, the noodles formed from the 2NapFF-Na are far more mechanically robust such that these can be lifted out of the gelling bath post formation (Figure 4c). These noodles show far greater alignment than those formed from 2NapFF-TBA, correlating with the higher degree of order seen for the precursor solutions by WAXS and POM (Figure 2). We can link successful noodle formation to the precursor solutions—effective cross-linking of aligned structures is required with both the higher viscosity of the 2NapFF-TBA and branching of the micellar solutions precluding robust noodles being formed. Of further interest, heating and cooling the noodles results in a shrinking in the diameter all cases (Figure 4d,e), but significantly greater shrinking for the 2NapFF-Na noodles; this correlates with a shrinking of the hollow core of the aligned micelles formed by 2NapFF-Na.^8^ Since the 2NapFF-TBA do not have this core, shrinking is not possible.

Electrospinning can be carried out with a range of macromolecules including many different polymers.^38^ With this polymer model in mind, we explored our 2NapFF solutions. Solutions of 2NapFF-Na were able to successfully begin forming thin, short electrospun fibers at a concentration of 40 mg/mL, improving significantly at 50 mg/mL (Figure 4f). These fibers exhibited a broad range of diameters from 0.1 to 3.5 μm (Figure 4h,i). In comparison, no successfully electrospun structures were formed from 2NapFF-TBA at any concentration trialed under otherwise identical experimental conditions as for 2NapFF-Na (Figure 4g). In polymer-based electrospinning, chain entanglement and intermolecular forces, along with other well-known factors, play critical roles in jet elongation and subsequent fiber formation.^38,39^ Our results can be understood through the conceptualization of wormlike micelles behaving as supramolecular polymer chains, with increasing concentration resulting in increased chain entanglement, as observed with the 2NapFF-Na series. While there are likely differences in charge density between the different micellar aggregates present in the two systems and potentially conductivity, we ascribe the difference in success in electrospinning of the two samples here to the viscosity. Excessive viscosity as shown by 2NapFF-TBA can be problematic in electrospinning due to the short time frame in which fiber formation occurs, where high viscosity samples do not allow adequate mobility of the molecules into elongated fibrous structures during the brief window of jet formation.

**Figure 5:**
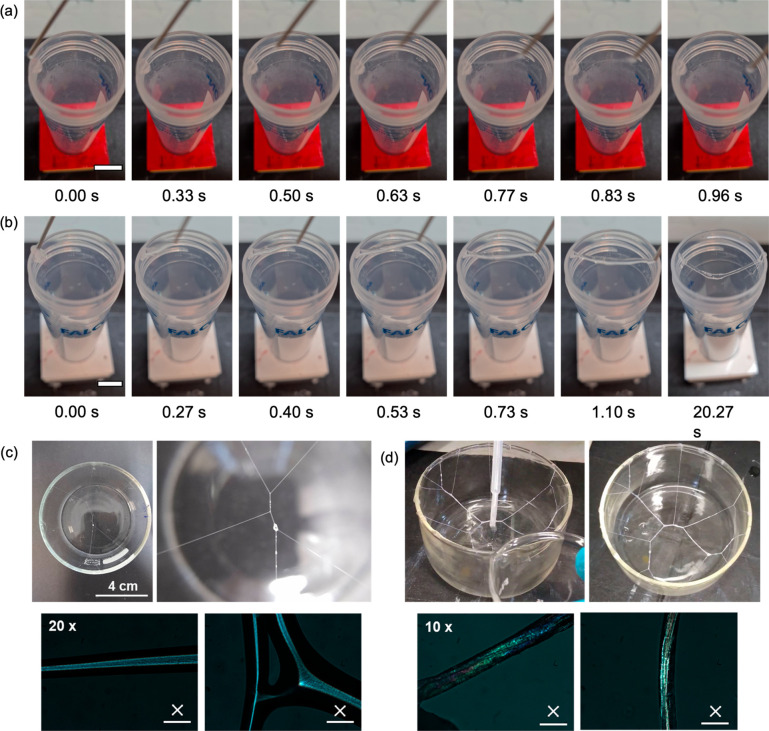
Frames (with time stamps) from a video showing the stringing of 75 mg/mL (a) 2NapFF-Na and (b) 2NapFF-TBA across a Falcon tube. (c) Photographs (above) and cross-polarized optical microscopy images (below, scale bars represent 0.1 mm) of the web formed after being air-dried overnight. (d) Photographs (above) and cross-polarized optical microscopy images (below, scale bars represent 0.2 mm) of an acidified web.

In comparison, the 2NapFF-TBA is the preferred solution for the formation of threads (Figure 5a). The high extensional viscosity for 2NapFF-TBA means it is possible to drag a solution between a gap and form persistent threads as long as 25 cm (Figure S51). In the analogous experiment with 2NapFF-Na, the thread breaks almost immediately (Figure 5a). The properties of the 2NapFF-TBA therefore allow weblike structures to be formed by extruding across space. Once bridged, the liquid threads are stable and can be dried (Figure S52). By sequentially adding and joining multiple threads, a weblike structure can be formed and dried overnight (Figure 5c). Alternatively, once the liquid thread structure has been formed, concentrated HCl can be added to the bottom of the beaker. The acidic vapor gels the threads, resulting in increased opacity and robustness (Figure 5d). Long strings (up to 77 cm) can be formed when allowed to fall freely with gravity (Figure S55).

## Conclusions

Hierarchical assembly is an effective means of preparing a range of useful, exciting materials. However, control of assembly across length scales is a difficult challenge, often confounded by the perceived need to redesign the molecular building blocks when new properties are needed. Here, we show that we can treat a simple dipeptide building block as a polyelectrolyte and use well-understood robust polymer physics approaches to understand the self-assembly of this LMWG over a wide concentration range, allowing us to prepare a range of different materials from a single building block. We show that we can collect scattering data for systems at a range of concentrations. If there is a peak at low *Q*, we can use this to determine whether the system is behaving as an extended polyelectrolyte; by comparing the onset of the slope and how quickly the correlation length drops, we can compare between systems formed using different counterions. We show, for example, that how entangled a system is correlates with how it might be best processed. From the 2D scattering data, we can determine whether alignment increases with concentration. If it does, then the system is behaving like a liquid crystal and (for example as shown here) it should form noodles. If alignment does not increase with concentration, then the system is forming branched or entangled micelles and will be more suitable to forming threads. From the data, we can also estimate at what concentration this macroscopic behavior should occur.

Previous work on such systems has focused on low concentrations (10 mg/mL and below). While there are differences at this concentration in terms of extensional and shear viscosity arising from the different micelles formed,^21^ moving here to higher concentrations has significantly extended the range of properties. Understanding the self-assembly process of these materials allows us to prepare interesting analogues to threads and webs as well as films that lose order on heating and noodles which change dimensions on heating, showing that we can transfer micellar changes to bulk properties. Our approach above can be used for other dipeptide-based systems (see Figures S56–S65), and we highlight that this understanding will likely transfer to other materials such as peptide amphiphiles^32,40^ and molecular rotors^33^ where the underlying principles are close. As such, this work shows that we can apply understanding from the well-understood field of polyelectrolytes to the more challenging field of self-assembled materials.

## Experimental Section

### Materials

All chemicals were purchased from Sigma-Aldrich and used as received. Deionized water was used throughout. 1ThNapFF was synthesized as described elsewhere.^41^

### Synthesis of 2NapFF

2NapFF was synthesized as previously described.^42,43^ Analytical data: ^1^H NMR ((CD_3_)_2_SO) δ 8.45 (1H, NH, d, *J* = 8.0 Hz) 8.13 (1H, NH, d, *J* = 8.5 Hz) 7.84 (2H, Ph, m) 7.73 (1H, Ph, m) 7.47 (1H, Ph, m) 7.36 (1H, Ph, m) 7.20 (11H, Ph, m) 4.65 (1H, CH, m) 4.54 (2H, CH_2_ from 2-napthoxyacetic acid, m) 4.48 (1H, CH, m) 2.98 (4H, CH_2_ Ph, m). ^13^C NMR ((CD_3_)_2_SO) δ 173.18, 171.30, 167.67 (C=O) 155.95, 137.98, 137,83, 134.49, 129.82, 129.71, 129.59, 129.21, 128.65, 128.44, 127.97, 127.25, 126.89, 126.71, 124.32, 118.92, 107.79 (aromatic C) 67.14 (CH_2_ from 2-napthoxyacetic acid), 53.96, 53.69 (CH) 37.88, 37.14 (CH_2_ Ph). Mass spec [M + Na]^+^ found at 519.1894 and calculated to be 519.1890.

### Preparation of 2NapFF Solutions

To produce a solution of 2NapFF requires a predetermined mass of 2NapFF, 1 molar equiv of a hydroxide (either sodium or tetrabutylammonium hydroxide) with respect to 2NapFF, and deionized water to make up the final volume (Table 1).^44^ Based on calculations to have a 1:1 molar ratio of 2NapFF to hydroxide, solutions of concentration 50 mg/mL or greater of 2NapFF required 1 M hydroxide. All others were prepared using 0.1 M hydroxide. All solutions were formed at room temperature (normally between 22 and 25 °C).

**Table 1:** Mass of 2NapFF and Volumes of Hydroxide and Water Needed to Make 1 mL of Various Concentrations of 2NapFF Solutions^a^

concentration of 2NapFF solution (mg/mL)	mass of 2NapFF (mg)	volume of 0.1 M sodium or tetrabutylammonium hydroxide solution (mL)	volume of water (mL)
5	5	0.10	0.90
10	10	0.20	0.80
15	15	0.30	0.70
20	20	0.40	0.60
25	25	0.50	0.50
30	30	0.60	0.40
35	35	0.70	0.30
40	40	0.81	0.19
45	45	0.91	0.09
50	50	0.10*	0.90
60	60	0.12*	0.88
75	75	0.15*	0.85
100	100	0.20*	0.80

a Asterisks represent when 1 M hydroxide was used to make the 1:1 XOH:2NapFF ratio possible.

### Preparation of Full Concentration Series Solutions

Three mL of each concentration of 2NapFF solution (5–100 mg/mL) were prepared using the quantities in Table 1, using NaOH or TBAOH. Solutions were made in Sterilin vials, wrapped in Parafilm, and left to stir overnight at 1000 rpm using 13 × 3 mm stirrer bars. The following day, once a homogeneous solution was present, the pH was adjusted to 10.5 ± 0.1 using 0.1 M, 1 and 2 M of the corresponding hydroxide; or 1 and 2 M HCl as required. All solutions were prepared in this way, unless specified.

### Preparation of Stirring Effects Solutions

17 mL of solution of 2NapFF-Na at a concentration of 40 mg/mL and 9 mL of 2NapFF solutions prepared at concentrations of 10 and 100 mg/mL were prepared using the quantities in Table 1. Solutions were made in 50 mL Falcon tubes, stirred with the same (25 × 8 mm) stirrer bars, and wrapped in Parafilm. Three different stirring methods were used on each sample over a 7-day period to establish if sample preparation history could affect the data collected. Samples were either stirred continuously at 400 rpm for 7 days, stirred at 1000 rpm for 7 days, or stirred overnight at 400 rpm and then left to stand undisturbed for the remaining six of the seven-day period. Viscosity measurements were performed every day for 7 days, with day 1 representing the day following the creation of the samples. The sample was discarded after viscosity measurement. The pH was adjusted every day to 10.5 ± 0.1 using 0.1, 1, and 2 M NaOH or 1 and 2 M HCl as required for each solution. This did not affect the overall concentration of the solutions as no more than 3 μL of base was added each day.

For 2NapFF-Na solutions prepared at a concentration of 40 mg/mL, solutions were also examined over a 7-day period but preparing the samples in 7 mL Sterilin vials instead of 50 mL Falcon tubes to establish if the containers used to prepare samples could also affect stirring and therefore sample viscosity. Seven lots of 3 mL of 2NapFF-Na samples at a concentration of 40 mg/mL were prepared using the quantities in Table 1. Solutions were made in 7 mL Sterilin vials, stirred with the same (13 × 3 mm) stirrer bars, and wrapped in Parafilm. Samples were then stirred at 1000 rpm for 7 days. Viscosity measurements were performed every day for 7 days, with day 1 representing the day following the creation of the samples. The pH was adjusted daily to 10.5 ± 0.1 using 0.1, 1, and 2 M NaOH or 1 and 2 M HCl as required for each solution.

For 2NapFF-Na solutions prepared at a concentration of 40 mg/mL, additional stirring effects samples were created—denoted as “recovery” experiments. 9 mL of 2NapFF solution at a concentration of 40 mg/mL was prepared using the quantities in Table 1. Solutions were made in 50 mL Falcon tubes, stirred with the same (25 × 8 mm) stirrer bars, and wrapped in Parafilm. Two different stirring methods were used on each sample over a 7-day period to establish if any sample preparation history could affect the data collected. Samples were either stirred at 400 or 1000 rpm for 3 days, and then both were left to stand undisturbed for the remaining four days of the 7-day period. Viscosity measurements were performed every day for 7 days, with day 1 representing the day following the creation of the samples. The pH was adjusted daily to 10.5 ± 0.1 using 0.1, 1, and 2 M NaOH or 1 and 2 M HCl as required for each solution.

A second “recovery” experiment using 2NapFF-Na at a concentration of 40 mg/mL was also created with an aim of establishing the effects of combined stirring and resting on viscosity. Four lots of 9 mL of 2NapFF solution at a concentration of 40 mg/mL were prepared using the quantities in Table 1. Solutions were made in 50 mL Falcon tubes, stirred with the same (25 × 8 mm) stirrer bars, and wrapped in Parafilm. Solutions were either stirred at 400 rpm for 1 day; stirred at 400 rpm for 7 days; stirred at 1000 rpm for 1 day; or stirred at 1000 rpm for 7 days. After these initial different stir periods, viscosity was measured. Solutions were then left undisturbed for a 7-day period. Viscosity measurements were then taken after this 7-day rest period. The pH was adjusted to pH 10.5 ± 0.1 using 0.1, 1, and 2 M NaOH or 1 and 2 M HCl as required for each solution before each viscosity measurement was performed.

### Preparation of Solutions for Viscosity Time Sweep

3 × 4 mL of 2NapFF-Na solutions at a concentration of 40 mg/mL were prepared using the quantities in Table 1. Solutions were made in Sterilin vials, wrapped in Parafilm, and left to stir for 2 days at 400 rpm using 13 × 3 mm stirrer bars. The pH was adjusted to pH 10.5 ± 0.1 using 0.1, 1, and 2 M NaOH or 1 and 2 M HCl as required.

### Rheology

Viscosity measurements were carried out using an Anton Paar Physica MCR101 rheometer. Measurements were performed using a 50 mm cone geometry (CP50) with gap distance between the geometry and the plate set to 0.101 mm and temperature set to 25 °C. All samples were poured onto the plate to minimize shearing that would be caused by pipetting the solutions. Fresh solution was used for all runs, unless otherwise stated. All viscosity measurements were carried out in duplicate, and values were averaged. Error bars represent the standard deviation between the replicates.

Viscosity measurements carried out when heating and cooling were carried out using an Anton Paar Physica MCR301 rheometer. Measurements were performed using a 50 mm cone geometry (CP50) with the gap distance between the geometry and the plate set to 0.101 mm and temperature cycled from 25 to 60 °C and back to 25 °C. Samples were poured onto the plate to minimize shearing that would be caused by pipetting the solutions. Fresh solution was used for each run.

Strain and frequency measurements were carried out using an Anton Paar Physica MCR101 rheometer, measuring 2 mL of sample in 7 mL Sterilin vials. Measurements were performed using a vane geometry with a gap distance between the geometry and the bottom of the sample vial set to 1.8 mm and temperature set to 25 °C. All strain and frequency measurements were carried out in triplicate and values averaged. Error bars represent the standard deviation between the replicates. Frequency sweeps were performed under a strain of 0.1%. Strain sweeps were performed at an angular frequency of 10 rad/s.

### Viscosity Time Sweep

In addition to regular viscosity measurements, additional viscosity measurements were performed using a CP50 geometry and 0.101 mm gap at 25 °C. For these additional viscosity measurements, the shear rate was held constant for 10 min at 1, 5, 10, 50, 100, 500, and 1000 s^–1^ before moving to the next shear rate to look for instability within the sample.

### Preshear of Solutions Using a Rheometer

In addition to regular viscosity measurements additional viscosity measurements were performed using a CP50 geometry and 0.101 mm gap at 25 °C. Two identical viscosity measurements were performed immediately one after the other on a single solution with the geometry remaining in the measuring position between the first and second viscosity measurements. Samples were left to sit under the CP50 geometry for about 2 min undisturbed between measurements.

### Dripping-onto-Substrate (DoS): Extensional Relaxation Time Measurements

The extensional relaxation times of the fluids were investigated using dripping-onto-substrate (DoS). DoS is a previously reported technique^27^ that involves video recording the dispensing of a droplet of fluid onto a substrate, resulting in the formation of an unstable liquid bridge that subsequently thins and breaks. The speed and shape with which the unstable liquid bridge breaks is analyzed to determine the extensional relaxation time.

The experiments were performed by dispensing a fluid from a 19G flat-headed needle (connected to a 10 mL syringe) onto a 4 mm diameter cylindrical glass substrate. The fluid was dispensed using an Alaris Carefusion syringe pump at a flow rate of 0.2 mL/h, but dispensing was stopped immediately prior to droplet contact. The thinning process was recorded on an iPhone 8 with a clip-on macrolense using either the Slo-Mo (240 frames per second (fps)) or video (30 fps) settings. Video recordings were converted from .mov files on the iPhone by default to individual frame-by-frame .tiff files. Then MatLab was used to analyze the thinning process. The conversion process require different software for each step as follows.

1. VLC media player (version 3.0.12) converts .mov to .mp4.^45^
2. FFmpeg (2021-04-04 build) converts .mp4 to .avi.^46^
3. ImageJ (version 1.52n) converts .avi to .tiff.^47^

The.tiff files were analyzed in MatLab (version R2021a)^48^ and the “canny edge” detection system used to extract the evolution of filament diameter with time.

NaOH and TBAOH 2NapFF at 25 mg/mL were studied both before and after a heat–cool cycle. Prior to the heat–cool the NaOH sample underwent a rapid thinning process (<1 s) whereas for the TBAOH sample the liquid bridge did not break. Instead, a thin filament formed but after 25 min dried out before breaking. After the heat–cool the NaOH sample exhibited solid-like behavior, where the droplet (prior to touching the substrate) possessed a nonspherical shape. Upon contact with the substrate no thinning is observed and the liquid bridge “jams”. For TBAOH post-heat–cool, the droplet makes contact with the substrate and broke over the course of 30–60 s. For the NaOH post-heat–cool and TBAOH pre-heat–cool, the liquid bridge did not break, and the data could not be analyzed.

For 2NapFF-Na pre-heat–cool and 2NapFF-TBA post-heat–cool, the process of thinning followed that as previously reported for elastic fluids (polyacrylamide and poly(ethylene oxide)^27,49^). The initial droplet contact and thinning processes are dominated by numerous factors such as surface tension, viscosity, and inertial forces. At later stages, a slender filament (where height is 10× width) forms and subsequently thins and breaks. This process is termed the elastocapillary regime and has been described by an equation by Entov and Hinch^50^ (eq 1)
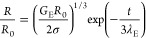
1where *G*_E_ is the elastic modulus, λ_E_ the extensional relaxation time, *R*_0_ the radius of the dispensing needle, *R* the filament radius, and σ the surface tension. The thinning of the slender filament is therefore fitted to an exponential decay to obtain the extensional relaxation time (λ_E_).

For 2NapFF-TBA post-heat–cool, the entire process was not videoed and analyzed using MatLab because it took 30–60 s. The volume of data would be impractical to analyze. Instead, only the slender filament region which is used to calculate λ_E_ was processed and analyzed.

### pH Measurements

A calibrated FC2020 Hanna pH probe was used to measure the pH of all solutions. The accuracy of the measurements stated by the supplier is ±0.1. The probe was calibrated with pH 4, 7, and 10 buffers. Measurements were carried out at room temperature (normally between 22 and 25 °C).

### Optical Microscope

Optical microscope images were collected using a Nikon Eclipse LV100 microscope at 5× magnification unless otherwise stated. Images were collected under polarized light. Solutions were made as described in Table 1 and transferred to a microscope slide by a cut plastic Pasteur pipet or by scooping for imaging. The plastic Pasteur pipet was cut to widen the pipet to try and reduce any shearing. Scale bars were added to images using the software ImageJ.

### SAXS and WAXS

Solutions were prepared as described above. To ensure aging was not an issue, samples were always run on day 7 following the creation of the solutions. Data were collected on a SAXSLAB Ganesha 300XL instrument (Xenocs) at the University of Bristol. Solutions were prepared as described in Table 1 with 70 μL then transferred to a 1.5 mm borosilicate glass capillary (Capillary Tube Supplies Ltd.). Samples were loaded into the capillaries using a wide bore glass Pasteur pipette. Higher concentration samples (typically over 50 mgmL^–1^ were extremely viscous and required brief centrifugation (1600 rpm, 60 s) to ensure that they were loaded into the capillary without any air bubbles. Capillaries were sealed with UV curable epoxy for 30 min (Norland) and measured for 3600 s in a *Q* range of 0.007–0.25 Å^–1^ for SAXS measurements and 600 s in a *Q* range of 0.07–2.8 Å^–1^ for WAXS measurements.

For systems based on 1ThNapFF, the samples were mounted horizontally in an Anton Paar SAXSPoint instrument. Wide- and small-angle X-ray scattering patterns were obtained with sample–detector distances 115 and 572 mm, respectively, using Cu Kα radiation (wavelength 1.54 Å), with 10 min exposure times. 2D scattering patterns were acquired on a Dectris Eiger detector and reduced by azimuthal integration into 1D radial profiles of intensity against scattering vector using Anton Paar SAXSAnalysis software.

In all cases, data were subsequently corrected for capillary and solvent (water) background and fitted using SasView software.^51^

### NMR Spectroscopy

Solutions were prepared as described above. 1 mL of each solution was pipetted into a 5 mm NMR tube. Using a 400 MHz Bruker spectrometer, ^2^H and ^23^Na NMR (if appropriate) spectra were collected as described previously^21^ and analyzed, and the phases were adjusted using TopSpin 4.0.7. Samples were heated to 60 °C inside the spectrometer and cooled back to 25 °C also in the spectrometer.

### Heat–Cool Inversion Tests

3 mL of all concentrations of 2NapFF-Na (5–100 mg/mL) and 2NapFF-TBA (5–75 mg/mL) were formed as described in Table 1. 2 mL was then pipetted into 14 mL glass vials and then placed into an oven set to 60 °C for 1 h. After 1 h, solutions were allowed to cool undisturbed on the bench at room temperature over 2 h. These were then inverted and monitored for instability every day for a 14 day period.

### Heat–Cool Rheology

10 × 3 mL of all concentrations of 2NapFF-Na (5–100 mg/mL) and 2NapFF-TBA (5–75 mg/mL) were formed as described in Table 1. Two mL were then pipetted into 7 mL Sterilin vials and then placed into an oven set to 60 °C for 1 h. After an hour, solutions were allowed to cool undisturbed on the bench at room temperature over 2 h. After 2 h, samples were examined using rheology.

### Copper Wire Heating

Solutions were prepared as described above. A copper wire (which had been placed in concentrated acid to remove its casing and then dried) was attached to a microscope slide using super glue. The wire was bent and attached to the slide such that solution could fill around and underneath it. The solution was poured/scooped onto the slide and placed under the microscope. The end of the copper wire not attached to the slide was heated with a Bosch heat gun on maximum setting for 30 min. Control samples were also prepared in the same way, but without the wire attached to the slide being heated. Images were taken under cross-polarized light during this time to monitor changes.

### Electrospinning

Solutions of 10–50 mg/mL 2NapFF were prepared as described above using either NaOH or TBAOH and slowly loaded into disposable polypropylene syringes. A syringe pump (model Alladin-8000, World Precision Instruments, UK) set to a flow rate of 0.2 mL/h supplied solution to a 20G needle tip (Hamilton Kel-F Hub blunt point needle) positively charged to 15 kV by a variable high voltage DC power supply (model 73030, Genvolt, Shropshire, UK). A tip-collector distance of 12 cm was used with a flat, electrically grounded target plate, coated in aluminum foil which was used as the collector material as well as glass microscope slides. Samples were produced in ambient environmental conditions and recorded within the ranges of 21.0–23.3 °C with a relative humidity of 30–40%.

### Scanning Electron Microscope (SEM)

SEM images were taken using a Quattro S environmental scanning electron microscope (Thermo Fisher Scientific, Waltham, MA). Imaging of materials collected on glass substrates was performed in low vacuum mode with a pressure range of 50–75 Pa and beam voltage of 10–15 kV using a low vacuum detector. Samples analyzed on foil substrates were imaged in high vacuum mode with a beam voltage of 5 kV using an Everhart–Thornley detector. Images were analyzed in ImageJ software to measure fiber diameters.

### Formation of Gel Noodles

10 mg/mL solutions were prepared as described above, using either NaOH or TBAOH. Gel noodles were formed using the injection of 10 μL of 2NapFF-Na or 2NapFF-TBA solution into trigger medium (50 mM CaCl_2_ adjusted to pH 10.5 with the corresponding hydroxide (NaOH or TBAOH)). A 2–20 μL pipette was used to perform a static injection as has been detailed in previously published work.^52^

For heat–cool noodle experiments, noodles were prepared using the syringe-pump and spin-coater setup as previously described. The flow rate was set to 100 mL/h with the spin-coater rotating at 100 rpm. The needle connected to the syringe pump was held vertically into the spinning CaCl_2_ solution (0.5 M). For the heat–cool noodles, the CaCl_2_ concentration was optimized to allow formation of mechanically robust noodles. When 50 mM CaCl_2_ was used, gelation was too slow and continuous noodles did not form. Noodles were formed from solutions of 2NapFF (10 mg/mL, pH 10.5) prepared using either NaOH or TBAOH as the base. The base used determines the counterion present within the solutions. The 2NapFF-Na noodles were strong enough to be lifted out of the Petri dish while the 2NapFF-TBA noodles broke down when we attempted to remove them from the Petri dish. We therefore prepared two sets of noodles for each counterion, a control sample and a heat–cool sample. The gel noodles were prepared for 3 s for each sample. This gave noodles of a reasonable size to allow imaging along the entire length of the noodle in each condition. For each sample, the noodles were first imaged under a light microscope. Then the Petri dish in which the noodles had been prepared was transferred directly to a water bath heated at 60 °C (or kept at room temperature for the control samples) and left for 1 h. After heating, the samples were left to cool for 1 h before being imaged again under the microscope to obtain the after images.

The gel noodles were imaged in the Petri dish in which they were formed on a moveable stage. This allowed for easy measurement with minimal disruption to the gels. A 1 mm scale bar at the same magnification was used to set a scale in ImageJ (version 1.52n). For each sample the noodle was imaged down the entire length resulting in 30 to 40 images. The diameter at 5 equidistant points along the noodle in each image collected was measured. This resulted in a large number of data points (70–250) for each sample meaning an average diameter and standard deviation could be calculated. With this a percentage reduction was calculated (so as to compare noodles of different started diameter). Box plots were prepared to show the size distributions.

### Threads

Solutions of 2NapFF at a concentration of 75 mg/mL were prepared as described in Table 1, using TBAOH or NaOH as required. Using a 2–20 μL pipet tip attached to a 1 mL syringe (the join was sealed with Parafilm), solutions could be drawn and strung across surfaces to create web patterns.

**Figure 6:**
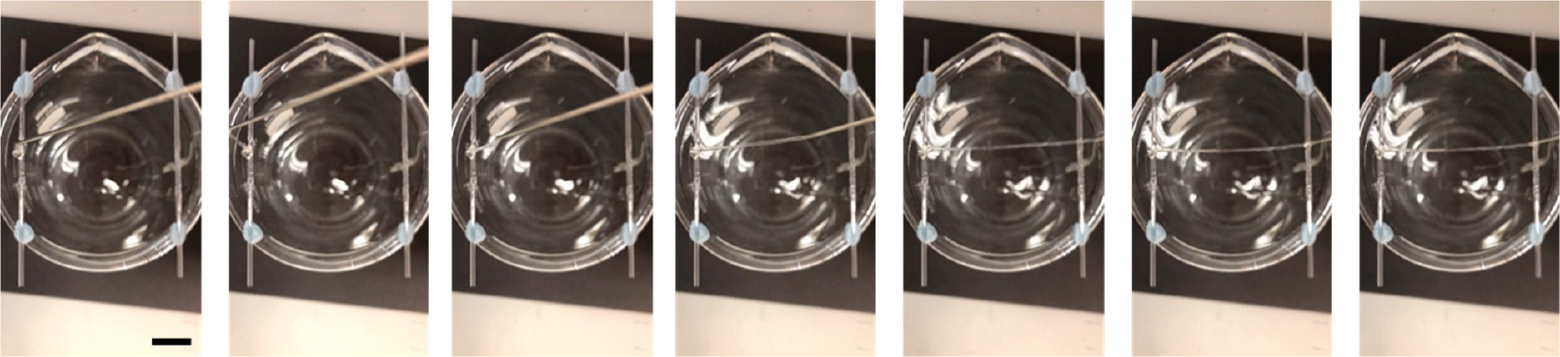
Liquid bridge formation using 75 mg/mL 2NapFF-TBA. Frames from a video showing the formation of a liquid bridge across a 6 cm gap. Scale bar represents 1.5 cm.

To form air-stable threads using the 2NapFF solution, a small amount of solution was dispensed onto a surface and then pulled vertically while slowly dispensing the fluid, resulting in a liquid thread being formed. As part of the solution is anchored to the surface, this facilitates stretching of the thread as it forms. With a liquid thread running from the surface to the end of the dispensing tip, the thread is then bridged/stretched across a gap. Once bridged, if the tip is simply pulled away, the thread acts as a rope and will be pulled away from the gap. Thus, the dispensing tip must be touched onto the surface of the beaker to “end” the thread as shown in Figure 6. The threads can be left uncovered in the open-air to dry overnight. Alternatively, 12 M HCl can be added to the bottom of the beaker, where the acidic vapor gels the threads.

### Fiber X-ray Diffraction

Samples were allowed to align overnight at room temperature by placing a 10 μL droplet between two wax-filled glass capillary tubes. The resulting fiber bundle was placed on a goniometer head, and X-ray fiber diffraction data was collected using a Rigaku rotating anode (CuKalpha) and Saturn CCD detector with exposure times of 30–60 s and specimen to detector distances of 50 and 100 mm. The diffraction patterns were examined using mosflm and converted to tiff format for analysis using CLEARER.^53^

## Supporting Information Available

Additional rheology data, full fitting data tables for SAXS, and further images of the systems (PDF)
