# Supplementary material for: Transferring Micellar Changes to Bulk Properties via Tunable Self-Assembly and Hierarchical Ordering
Source: ACS Nano. 2022 Nov 28;16(12):20497–509. doi: 10.1021/acsnano.2c06898 (PMC9798853; doi:10.1021/acsnano.2c06898)
Supplement: Supplementary file 1 — nn2c06898_si_001.pdf [file nn2c06898_si_001.pdf]

Transferring micellar changes to bulk properties via tunable self-assembly and hierarchical ordering

Lisa Thomson,[a] Daniel McDowall,[a] Libby Marshall,[a] Olivia Marshall,[a] Henry Ng,[b] W. Joseph A. Homer,[c] Dipankar Ghosh,[a] Wanli Liu,[d] Adam M. Squires,[d] Eirini Theodosiou,[c] Paul D. Topham,[c] Louise C. Serpell,[e] Robert J. Poole,[b] Annela Seddon[f] and Dave J. Adams[a,]**

a School of Chemistry, University of Glasgow, Glasgow, G12 8QQ, U.K.

b School of Engineering, University of Liverpool, Liverpool, L69 3GH, U.K.

c Aston Institute of Materials Research, Aston University, Birmingham, B4 7ET, U.K.

d Department of Chemistry, University of Bath, Bath BA2 7AY, U.K.

e Sussex Neuroscience, School of Life Sciences, University of Sussex, Falmer, BN1 9QG, U.K.

f School of Physics, HH Wills Physics Laboratory, University of Bristol, Tyndall Avenue, Bristol, BS8 1TL, U.K.

SUPPORTING INFORMATION

1. Discussion of Sample Preparation and Sample History

Wormlike micelles are considered a "living" system.[1-4] Wormlike micelles are transient and have unique viscoelastic behaviour.[1] Under shear, the micelles can continuously break and reform.[2] Many other factors are known to influence micellar growth and properties such as pH,[4, 5] the addition of salt,[6-8] and temperature.[9, 10] We investigate here the effects of shear specifically to gain the most reproducible data possible from these living systems. We selected exemplar solution concentrations of 10, 40 and 100 mg/mL 2NapFF-Na based on our SAXS data. These concentrations have different structures and packing, as discussed in the main text, and hence could be affected differently by the investigated factors.

1.1. Stirring Effects and Sample Aging

As a "living" system, wormlike micelles continuously break and reform when shear is applied,[2] and rheological properties (for example, as previously shown by a self-assembled flavonoid liquid crystal system) are known to be shear history dependent.[11] To make our samples, we must apply a shear by stirring to give a homogeneous solution. Hence, we investigate the rate of stirring and time of stirring on our 2NapFF samples at concentrations of 10, 40 and 100 mg/mL.

Samples were stirred at three different rates, keeping the container and stirrer bar size constant. Stirring method 1 stirs the samples continuously at 400 rpm (set by stirrer plate) for seven days. Method 2 stirs the samples continuously at 1000 rpm (set by stirrer plate) for seven days. Method 3 stirs the samples overnight at 400 rpm (set by stirrer plate) to create a homogeneous sample and are then left undisturbed to stand for the remaining six days. Data collected are shown in Figure S1. Using stirring method 1 (400 rpm) we see in general a gradual decrease in viscosity over the seven-day period. Using stirring method 2 (1000 rpm), we see solutions of concentration 10 and 40 mg/mL 2NapFF come to equilibrium viscosity after one day stirring at this higher shear rate. Comparing the 40 mg/mL solutions stirred at 400 rpm and 1000 rpm, the equilibrium viscosity is established quicker at the higher shear rate. At 1000 rpm stirring, equilibrium is reached after one day, however when stirred at the slower rate of 400 rpm, the equilibrium viscosity is not reached until day five.

Fluid properties are known to be time-dependent due to entanglement rates.[12] The rate of entanglement is slow and based on Brownian-motion. However, disentanglement is driven by strain/shear and is therefore quicker.[12, 13] Hence, when our samples are allowed to stand undisturbed on the bench, we remove the shear which would be responsible for continuously breaking the micelles. We therefore are allowing the wormlike micelles the chance to slowly grow and entangle, allowing the viscoelasticity of the solutions to increase.[14] We see shear history impacts the rheological properties for 2NapFF concentrations of 10 and 40 mg/mL, where the viscosity gradually increases over the seven day period when left undisturbed. For 100 mg/mL, however, we see a gradual decrease in viscosity over the seven-day period when left undisturbed. We hypothesis that this is due to how viscous and concentrated these samples are. This concentration was visibly very viscous and moving onto the rheometer and lowering the geometry for measurement may have disturbed the samples. Lowering the geometry may have caused premature alignment before measuring, which would decrease the viscosity. The lowering of the geometry for measurement could have also applied shear to the sample which could have broken some wormlike micelles, again lowering the viscosity prematurely.

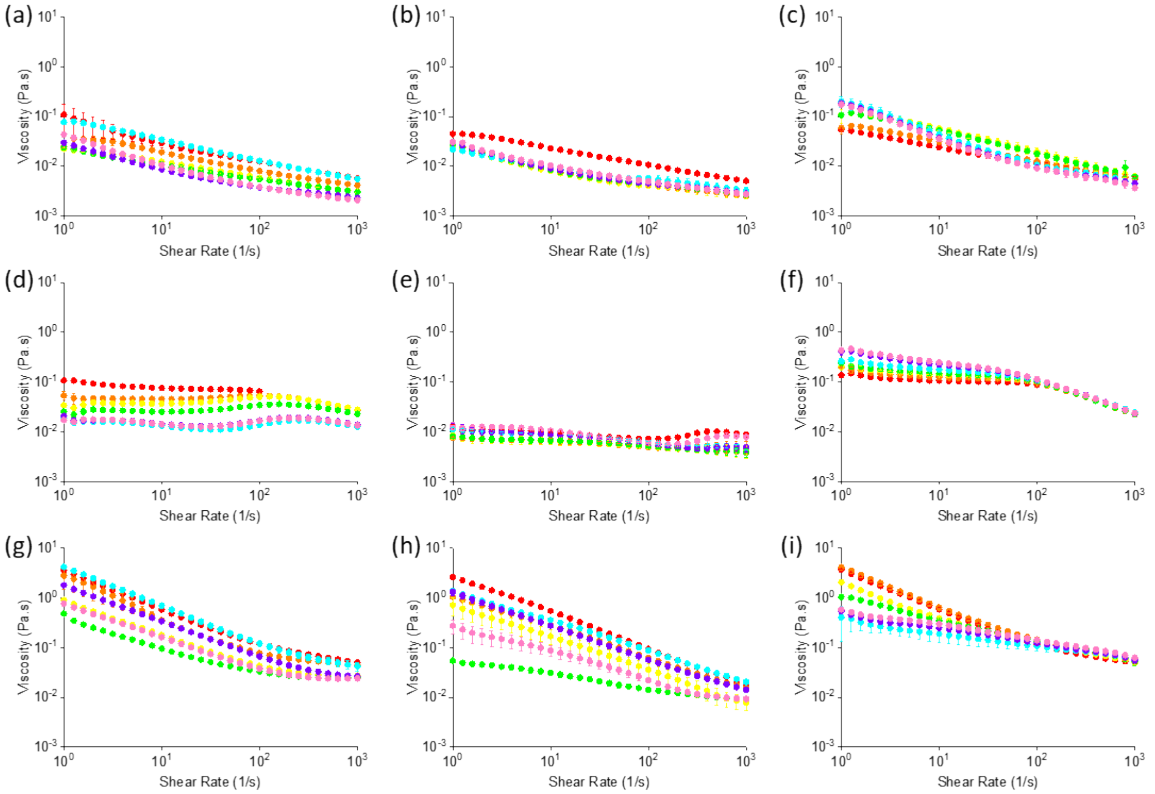

Figure S1. Viscosity data for solutions of 2NapFF at (a)-(c) 10 mg/mL; (d)-(f) 40 mg/mL; (g)-(i) 100 mg/mL at pH 10.5 whilst varying stirring over a seven-day period. Plots show viscosity after day 1 (red, the day following the creation of the samples), day 2 (orange), day 3 (yellow), day 4 (green), day 5 (light blue), day 6 (purple) and day 7 (pink). Samples were: (a), (d), (g) stirred continuously at 400 rpm for seven days; (b), (e), (h) stirred continuously at 1000 rpm for seven days; (c), (f), (i) stirred overnight at 400 rpm, then left undisturbed to stand for the remaining six days. Data was collected in duplicate with error bars showing the standard deviation between the measurements. Following each viscosity measurement, any used sample was removed from the rheometer and discarded to ensure further runs only used fresh sample.

In addition to the rate of stirring, the containers and stirrer bars used to prepare the samples also affects the sample viscosity, Figure S2. Stirring consistently at 1000 rpm for both sample containers, we see clear differences in viscosity trend over the seven-day monitoring period. For samples prepared in Falcon tubes and stirred with 25x8 mm stirrer bars, Figure S3a, the viscosity comes to a steady state equilibrium after only one day of stirring at 1000 rpm. However, when stirring in a smaller container (Sterilin vial) and using a smaller stirrer bar (13x3 mm), we see a general decreasing trend in viscosity as stirring time is increased. It is therefore important to consistently prepare the samples by stirring at the same rates, but also by stirring in the same containers.

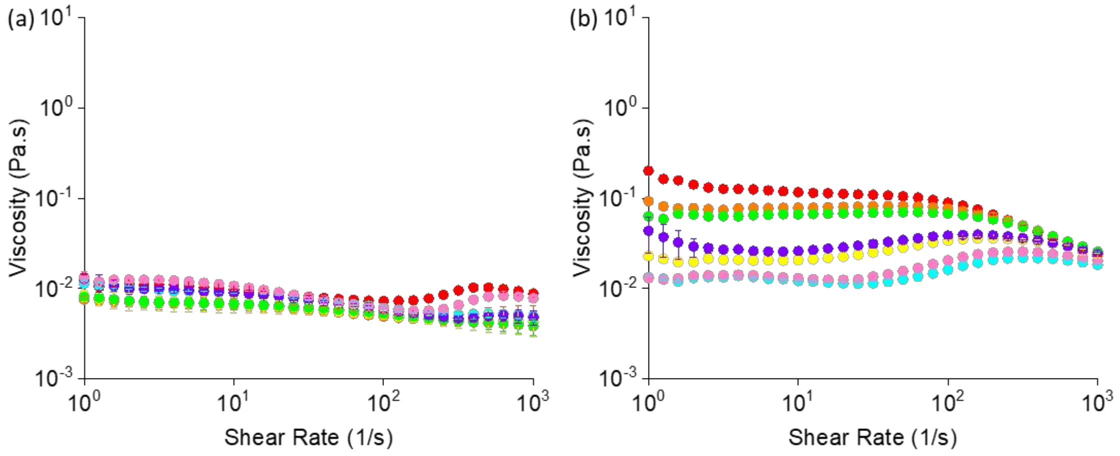

Figure S2. Viscosity data for solutions of 2NapFF at 40 mg/mL at pH 10.5, keeping constant stirring rate of 1000 rpm over a seven-day period, prepared in (a) a falcon tube and 25x8 mm stirrer bar and (b) a 7 mL Sterilin vial and 13x3 mm stirrer bar. Plots show viscosity after day 1 (red, the day following the creation of the samples), day 2 (orange), day 3 (yellow), day 4 (green), day 5 (light blue), day 6 (purple) and day 7 (pink). Data was collected in duplicate with error bars showing the standard deviation between the measurements. Following each viscosity measurement, any used sample was removed from the rheometer and discarded to ensure further runs only used fresh sample.

Additional stirring experiments were also created – denoted as "recovery" experiments. Here, we examine our 40 mg/mL 2NapFF concentration solutions whilst combining stirring methods. First, we stirred samples at either 400 rpm or 1000 rpm continuously for three days, then samples were left to stand undisturbed on the bench for the remaining four days (Figure S3). Combining the stirring and standing methods for these samples, we see the viscosity remain constant while stirring. When stirring is stopped, the viscosity increases, like that seen in Figure S1f. The viscosity come to equilibrium after a total of five days for samples stirred at 400 rpm and after six days for the samples stirred at 1000 rpm.

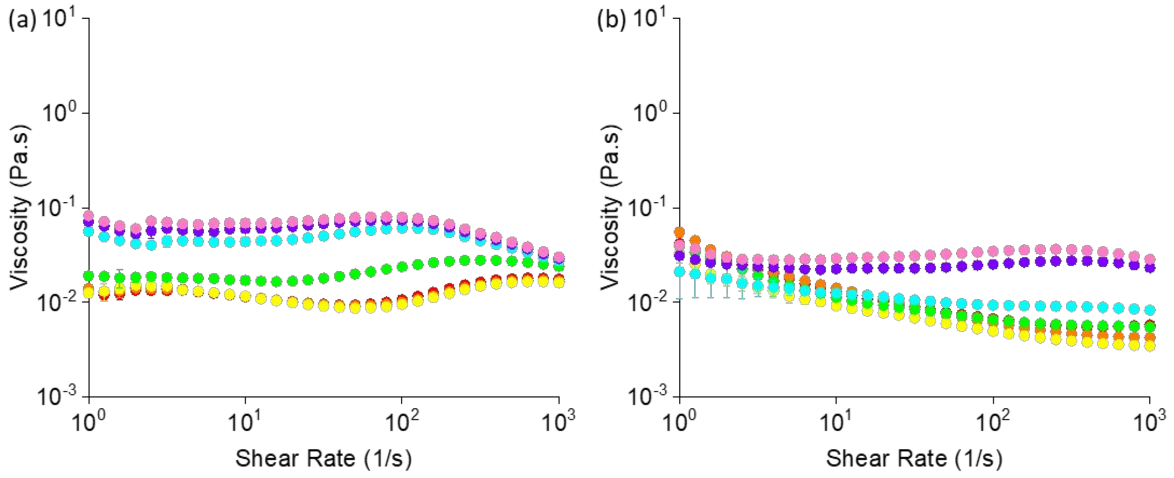

Figure S3. Viscosity data for solutions of 2NapFF at 40 mg/mL at pH 10.5 whilst varying stirring over a seven-day period; showing day 1 (red, the day following the creation of the samples), day 2 (orange), day 3 (yellow), day 4 (green), day 5 (light blue), day 6 (purple) and day 7 (pink). Samples were (a) stirred for 3 days at 400 rpm, then left undisturbed to stand for the remaining four days; (b) stirred for 3 days at 1000 rpm, then left undisturbed to stand for the remaining four days. Data was collected in duplicate with error bars showing the standard deviation between the measurements. Following each viscosity measurement, any used sample was removed from the rheometer and discarded to ensure further runs only used fresh sample.

To further examine the effects of combined stirring methods and viscosity steady state equilibrium, additional "recovery" stirring experiments were also created. Here, we examine our 40 mg/mL 2NapFF concentration solutions at either 400 rpm or 1000 rpm stirred for either one day or seven days. All samples were then left to stand undisturbed on the bench for a seven-day period after stirring. We aim to establish the effects of different stirring rates and times on the viscosity increases we have already noted when samples are left undisturbed after stirring. Combining the 400 rpm stirring rate and resting, Figure S4(a), we see similar viscosity plot shapes to those seen in Figure S1d. We also see the viscosity increase like that shown in Figure S1f. The size of the viscosity increase over the seven day standing period is approximately half an order of magnitude greater, regardless if samples were originally stirred at 400 rpm for one day or for seven days. However, when we combine stirring at 1000 rpm and then resting, Figure S5b, we see similar to Figure S1e that the viscosity of samples stirred at 1000 rpm comes to equilibrium after one day. Unlike when stirring at 400 rpm, we do not see an increase in viscosity once stirring has stopped. As the micelles have been stirred more at 1000 rpm compared to 400 rpm, we assume the micelles have been broken more during shear applied when stirring and hence in the same stand time period, we do not see the micelles entangle as quickly to increase the viscosity when shear is removed.

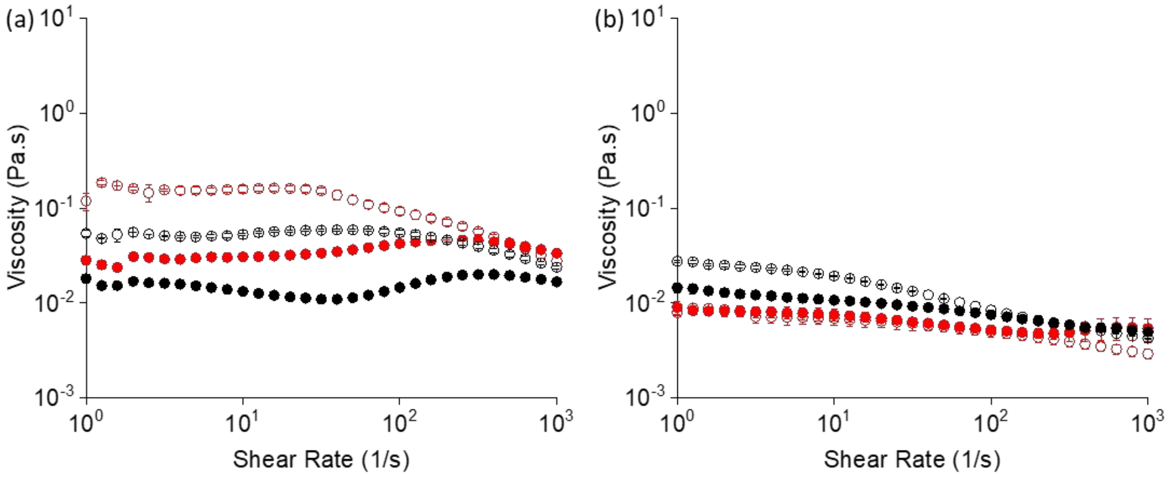

Figure S4. Viscosity data for solutions of 2NapFF at 40 mg/mL at pH 10.5 to examine the effects of stirring at different rates and then removing shear. Samples were stirred at (a) 400 rpm and (b) 1000 rpm for one day (filled red, the day following the creation of the samples); and seven days (filled black), and then left undisturbed for seven days following stirring. Viscosity measured after the seven-day rest period is represented by hollow red circles for samples stirred for one day before the rest period and hollow black circles for samples stirred for seven days before the rest period. Data was collected in duplicate with error bars showing the standard deviation between the measurements. Following each viscosity measurement, any used sample was removed from the rheometer and discarded to ensure further runs only used fresh sample.

1.2. Sample Instability

We have previously shown that the viscosity of 2NapFF solutions at concentrations of 10 mg/mL or lower show shear thinning behaviour when shear rate is increased.[15] Liquid crystal systems, due to their preferred orientation, can show more complex viscosity behaviour.[16] Combinations of shear thinning, shear thickening and plateaus can be observed within a single sample. To prove that these are true features and not simply sample artefacts, we check for any time dependency and instability within these 2NapFF samples, using the 40 mg/mL concentration sample as an exemplar. We begin by set the rheometer to hold a constant shear rate values of 1, 5, 10, 50, 100, 500 and 1000 s^{-1} for 10 minutes before moving to the next (Figure S5). We can see over the 10 minute period of constant shear at each rate there is no considerable change in viscosity. This suggests the shear thickening/plateaus seen in the 40 mg/mL samples are not simply due to a time dependence or instability.

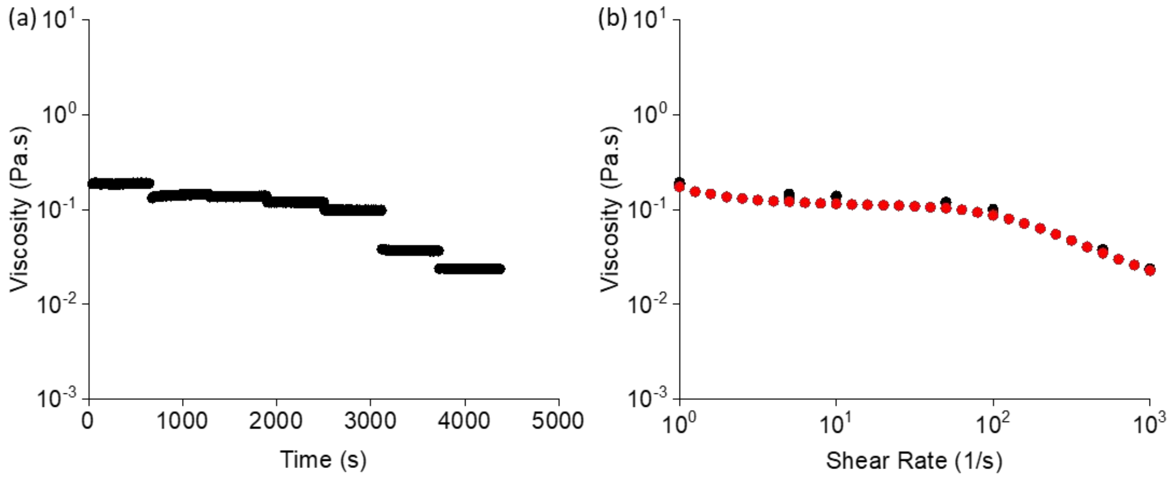

Figure S5. Viscosity time sweep for 2NapFF solutions at pH 10.5 at a concentration of 40 mg/mL, stirred for 2 days before measurement. (a) Full viscosity data collected when shear rate was held constant for 10 minutes at 1, 5, 10, 50, 100, 500 and 1000 s^{-1}. (b) Regular viscosity run of 2NapFF at pH 10.5 (red) at a concentration of 40 mg/mL and viscosity data, taken from (a), when holding the shear rate constant (black), to compare. Error bars represent the standard deviation between measurements, which were carried out in duplicate.

Additionally, we examined the effects of pre-shearing on our 2NapFF solutions at concentrations of 10, 40 and 100 mg/mL. As previously stated, wormlike micelles constantly break and reform when shear is applied.[2] To again check for instability and sample memory and history, we use the rheometer to pre-shear our samples. Here, we use the same sample to perform two consecutive viscosity sweeps at each investigated concentration. From our data in Figure S6, we see that our samples are stable after pre-shearing and does not alter the viscosity for concentrations of 10 mg/mL and 40 mg/mL 2NapFF. For solutions at a concentration of 100 mg/mL 2NapFF, there is a small decrease in viscosity caused by pre-shearing.

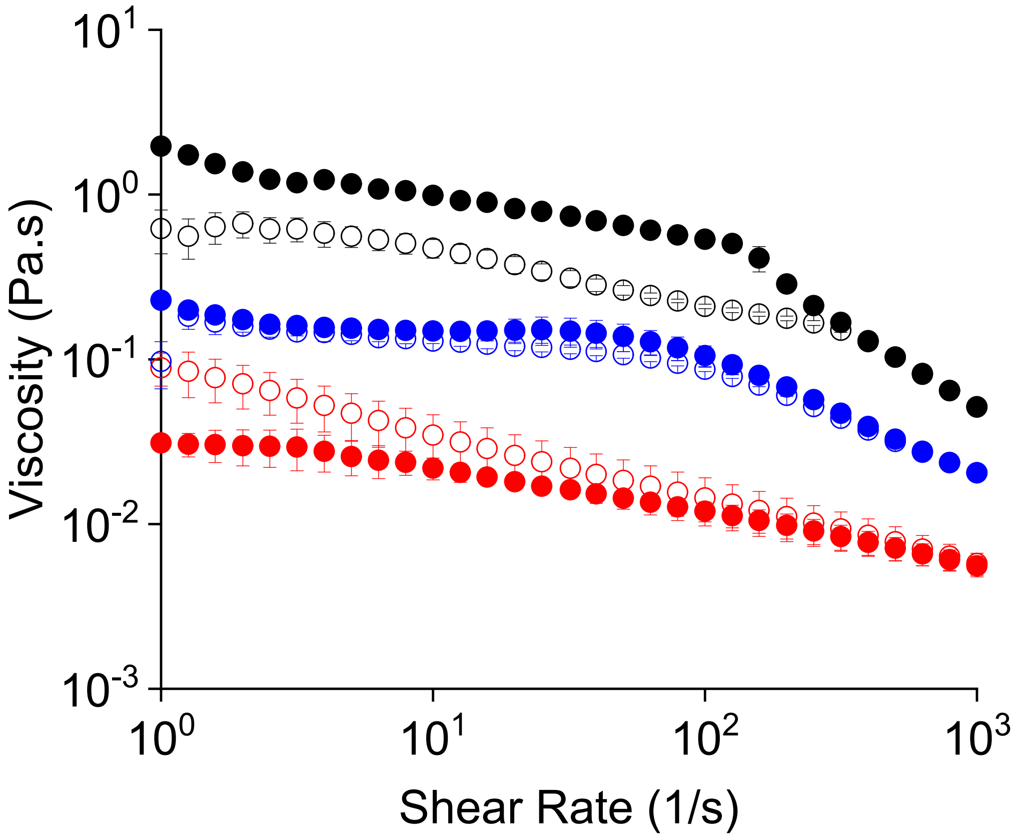

Figure S6. Viscosity data for 2NapFF solutions at pH 10.5 at a concentration of 10 mg/mL (red); 40 mg/mL (blue); 100 mg/mL (black) which have been pre-sheared using the rheometer. Filled circles represent the first viscosity measurement and empty circles represent the second viscosity measurement of the same sample. The geometry was not lifted between the first and second viscosity measurements and samples were left to sit for about two minutes undisturbed between measurements. Error bars represent the standard deviation between measurements, which were carried out in duplicate.

2. Supplementary Figures

1D SAXS data

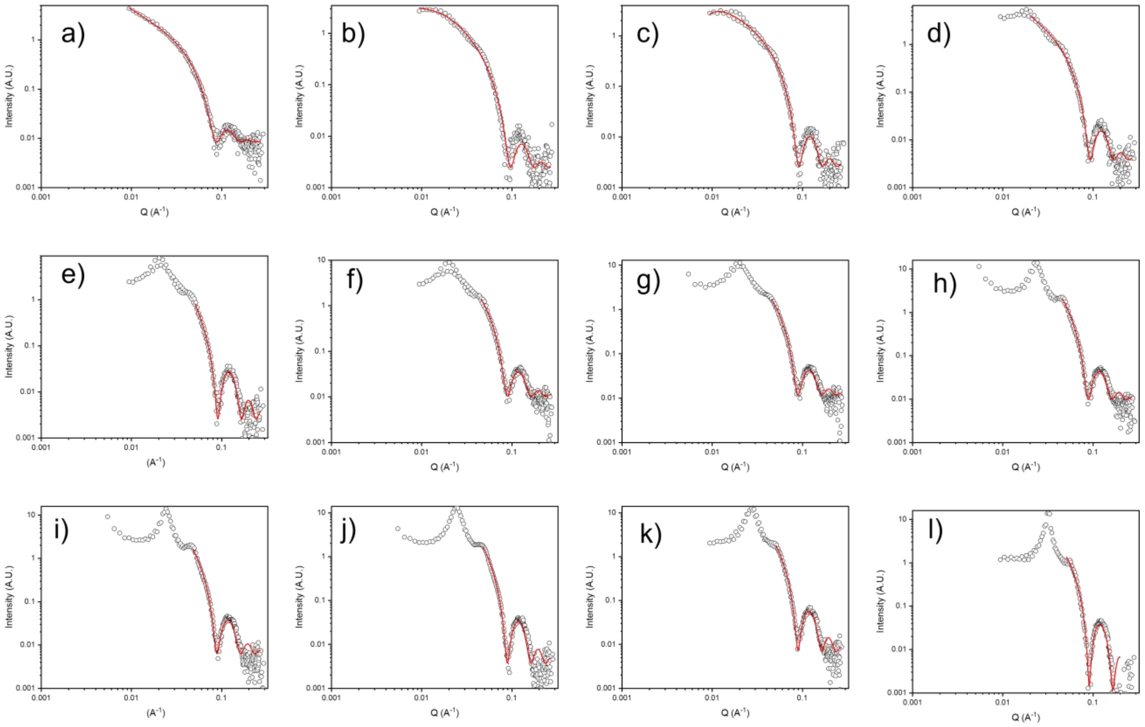

Figure S7. 1D SAXS data (black open circles) with form factor fits (red line) for 2NapFF-Na; a) 10 mg/mL; b) 15 mg/mL; c) 20 mg/mL; d) 25 mg/mL; e) 30 mg/mL; f) 35 mg/mL; g) 40 mg/mL; h) 45 mg/mL; i) 50 mg/mL; j) 60 mg/mL; k) 75 mg/mL; l) 100 mg/mL.

Table S1. Fitting parameters for SAXS data taken for increasing concentration of 2NapFF-Na.

**Concentration / (mg/mL)**	**100**	**75**	**60**	**50**	**45**	**40**	**35**
Model	Cylinder	Cylinder	Cylinder	Flexible cylinder	Flexible cylinder	Flexible cylinder	Flexible cylinder
Background	0.0008 ± 0.0001	0.0034 ± 0.0002	0.015 ± 0.0002	0.0063 ± 0.0002	0.0097 ± 0.0002	0.0118 ± 0.0002	0.0103 ± 0.0002
Scale	0.006 ± 2.6 x 10^{-5}	0.005 ± 5.8 x 10^{-5}	0.005 ± 3.0 x 10^{-5}	0.004 ± 7.2 x 10^{-5}	0.004 ± 7.5 x 10^{-5}	0.004 ± 6.4 x 10^{-5}	0.003 ± 5.1 x 10^{-5}
Length / Å	>1000	>1000	>1000	>1000	>1000	>1000	>1000
Kuhn Length / Å			53.1 ± 2.1	66.1 ± 4.3	69.8 ± 4.7	130.0 ± 15.7	97.7 ± 11.8
Radius / Å	42.7 ± 0.04	42.7 ± 0.04	42.7 ± 0.05	43.1 ± 0.05	43.1 ± 0.06	42.8 ± 0.07	42.7 ± 0.07
χ^2	13.65	8.17	5.91	11.79	10.81	6.18	6.92
	**30**	**25**	**20**	**15**	**10**		
Model	Flexible cylinder	Flexible cylinder	Flexible cylinder	Flexible cylinder	Flexible cylinder		
Background	0.0036 ± 0.0001	0.0040 ± 0.0001	0.0026 ± 0.0001	0.0023 ± 0.0001	0.0084 ± 0.0001		
Scale	0.003 ± 4.5 x 10^{-5}	0.002 ± 1.52 x 10^{-5}	0.001 ± 3.2 x 10^{-6}	0.001 ± 3.1 x 10^{-6}	0.001 ± 3.2 x 10^{-6}		
Length / Å	>1000	>1000	144.48 ± 23.4	250.2 ± 3.1	>1000		
Kuhn Length / Å	53.9 ± 0.6	209.3 ± 155.5	382.9 ± 18.6	196.3 ± 5.7	760.5 ± 121.1		
Radius / Å	42.2 ± 0.07	41.0 ± 0.09	41.8 ± 0.09	41.2 ± 0.1	43.5 ± 0.1		
χ^2	4.15	16.76	12.85	5.45	2.15		

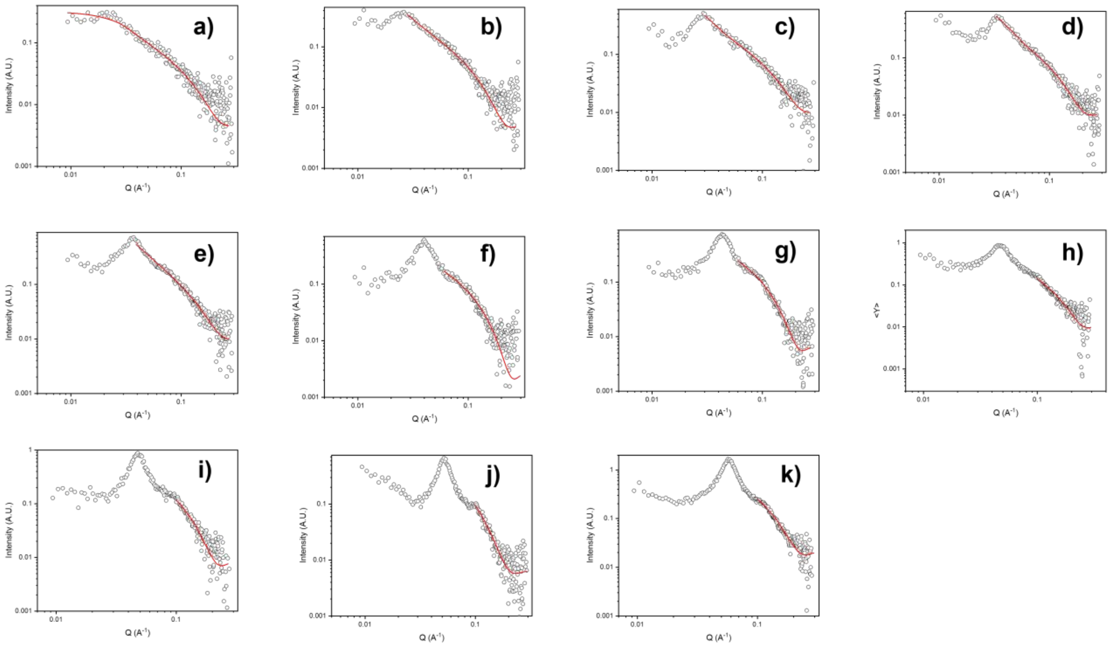

Figure S8. 1D SAXS data (black open circles) with form factor fits (red line) for TBA-2NapFF-TBA; a) 10 mg/mL; b) 15 mg/mL; c) 20 mg/mL; d) 25 mg/mL; e) 30 mg/mL; f) 35 mg/mL; g) 40 mg/mL; h) 45 mg/mL; i) 50 mg/mL; j) 60 mg/mL; k) 75 mg/mL.

Table S2. Fitting parameters for SAXS data taken for increasing concentration of 2NapFF-TBA.

**Concentration (mg/mL)**	**10**	**15**	**20**	**25**	**30**	**35**	**40**	**45**	**50**	**60**	**75**
**Model**	Flexible Cylinder	Flexible Cylinder	Flexible Cylinder	Flexible Cylinder	Flexible Cylinder	Flexible Cylinder	Cylinder	Cylinder	Cylinder	Flexible Cylinder	Cylinder
**Scale**	0.0009 ± 2.8 x 10^{-5}	0.001 ± 2.5 x 10^{-5}	0.002 ± 4.4 x 10^{-5}	0.002 ± 7.0 x 10^{-5}	0.002 ± 0.0002	0.002 ± 5.5 x 10^{-5}	0.002 ± 8.8 x 10^{-5}	0.01 ± 0.0003	0.01± 0.0002	0.001 ± 7.3 x 10^{-5}	0.02 ± 0.0002
**Background**	0.005 ± 0.0003	0.005 ± 0.0003	0.009 ± 0.0004	0.01 ± 0.0003	0.01 ± 0.0004	0.002 ± 0.0002	0.01 ± 0.0005	0.01 ± 0.0009	0.007 (fixed)	0.06 ± 0.0003	0.02 ± 0.0008
**Length / Å**	197.1 ± 11.0	316.1 ± 123.2	>1000	>1000	>1000	>1000	>1000	>1000	>1000	>1000	>1000
**Kuhn Length / Å**	112.2 ± 11.4	110.2 ± 13.0	69.8 ± 3.6	54.3 ± 2.2	38.4 ± 6.8	>1000				3.9 ± 0.08	
**Radius / Å**	14.1 ± 0.4	15.3 ± 0.3	14.3 ± 0.3	15.9 ± 0.3	13.8 ± 0.4	14.6 ± 0.3	16.6 ± 0.3	13.6 ± 0.3	16.0 ± 0.2	16.7 ± 0.2	15.3 ± 0.2
χ^2	2.33	2.41	2.40	2.37	2.79	2.17	1.84	2.73	34.6	1.85	2.50

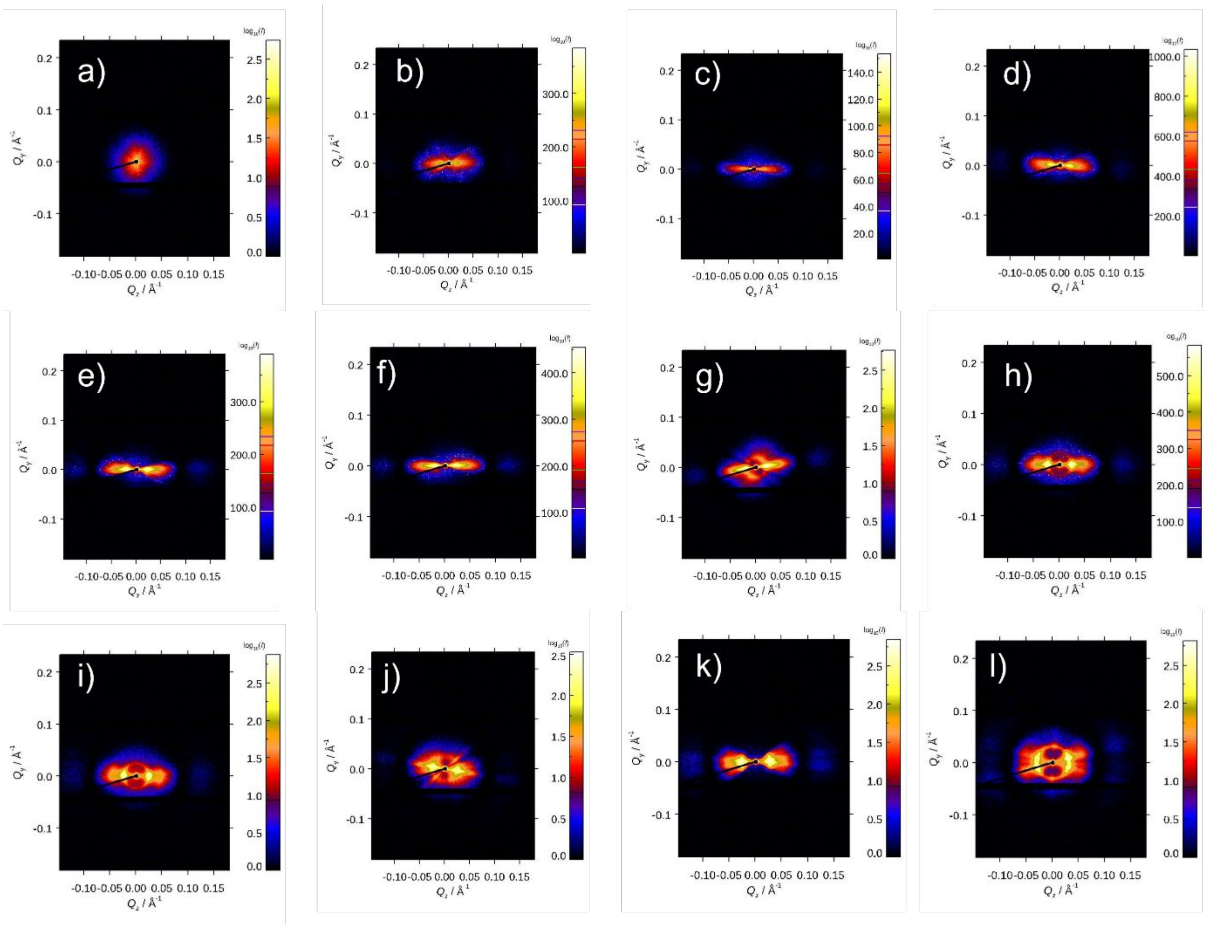

Figure S9. 2D SAXS patterns for Na-2NapFF; a) 10 mg/mL; b) 15 mg/mL; c) 20 mg/mL; d) 25 mg/mL; e) 30 mg/mL; f) 35 mg/mL; g) 40 mg/mL; h) 45 mg/mL; i) 50 mg/mL; j) 60 mg/mL; k) 75 mg/mL; l) 100 mg/mL.

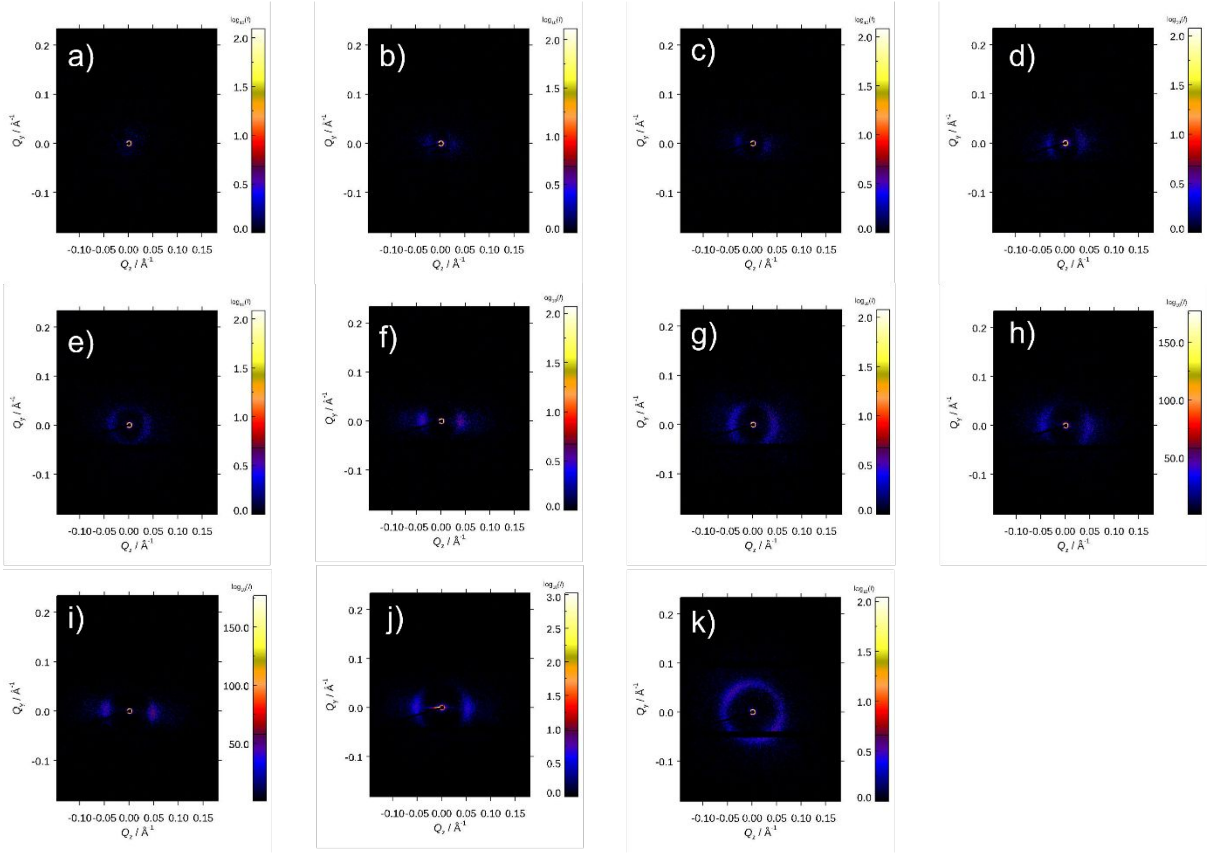

Figure S10. 2D SAXS patterns for 2NapFF-TBA; a) 10 mg/mL; b) 15 mg/mL; c) 20 mg/mL; d) 25 mg/mL; e) 30 mg/mL; f) 35 mg/mL; g) 40 mg/mL; h) 45 mg/mL; i) 50 mg/mL; j) 60 mg/mL; k) 75 mg/mL.

<P2> order parameter

Data were fitted using the in-house written IDL based software programme SEE, developed and kindly shared by Prof. Robert Richardson. 2D data files were regrouped by selecting the position of the first scattering ring with background boxes. <P2> was then calculated from a fit to the I v Φ data using the analysis described in Sims *et al.*[17]

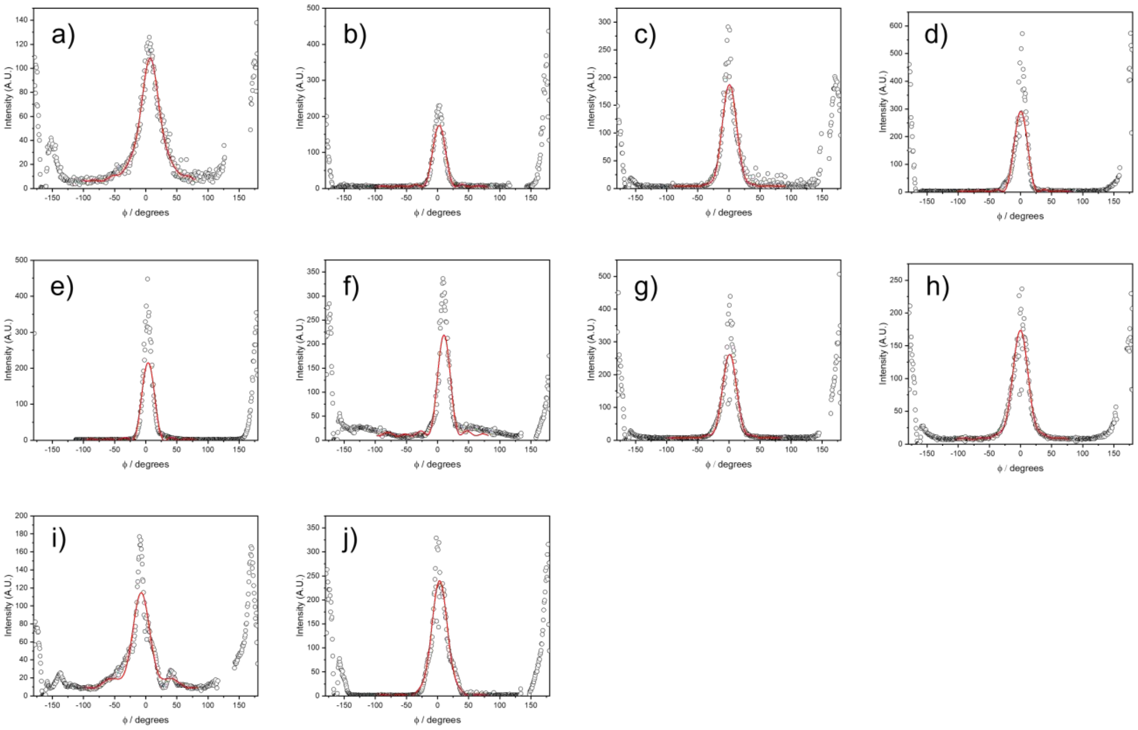

Figure S11. Fits of I v Φ data for Na-2NapFF; a) 15 mg/mL; b) 20 mg/mL; c) 25 mg/mL; d) 30 mg/mL; e) 35 mg/mL; f) 40 mg/mL; g) 45 mg/mL; h) 50 mg/mL; i) 60 mg/mL; j) 75 mg/mL.

Correlation length v concentration

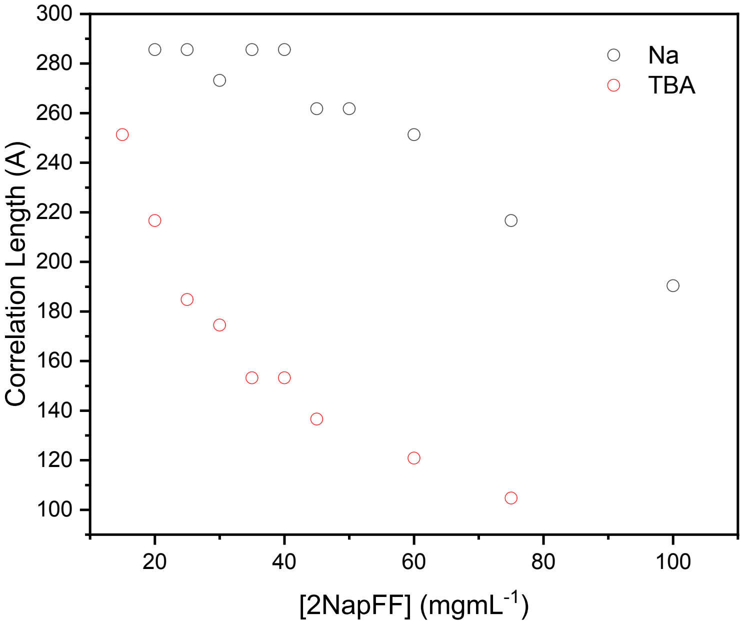

Figure S12. Plot of the correlation length (calculated from the Q* peak) against concentration for 2NapFF-Na and 2NapFF-TBA

WAXS data

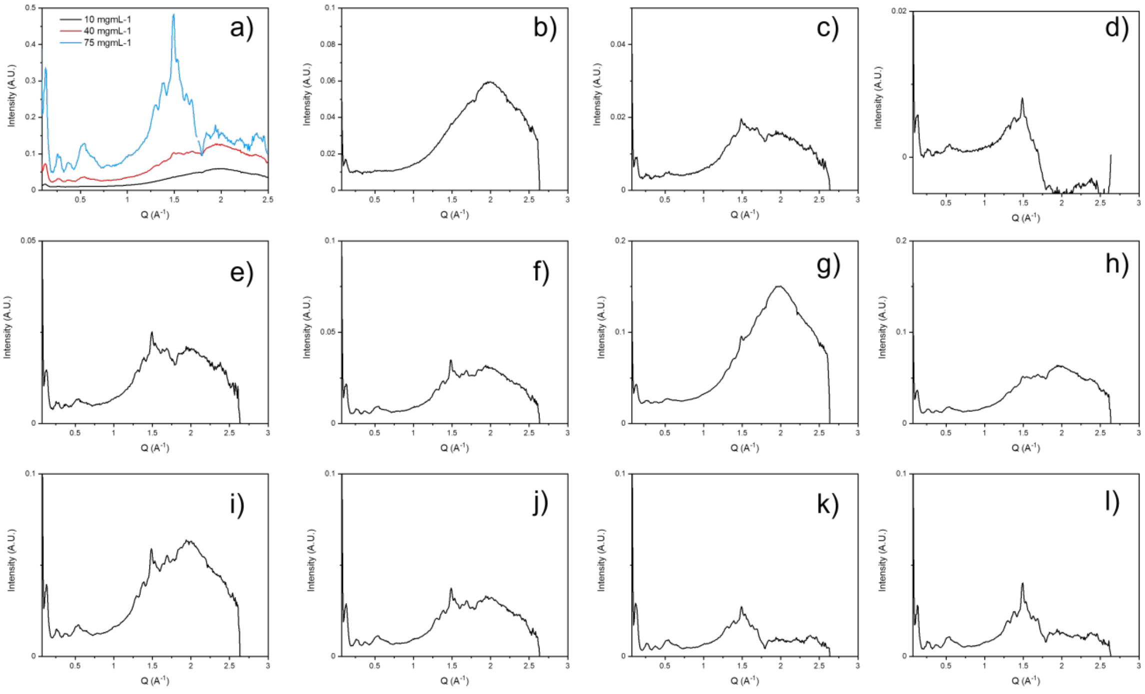

Figure S13. Plot of WAXS data for 2NapFF-Na: a) 10, 40 and 75 mg/mL plotted (with arbitrary scaling) for comparison; b) 10 mg/mL; c) 15 mg/mL; d) 20 mg/mL; e) 25 mg/mL; f) 30 mg/mL; g) 35 mg/mL; h) 40 mg/mL; i) 45 mg/mL; j) 50 mg/mL; k) 60 mg/mL; l) 75 mg/mL.

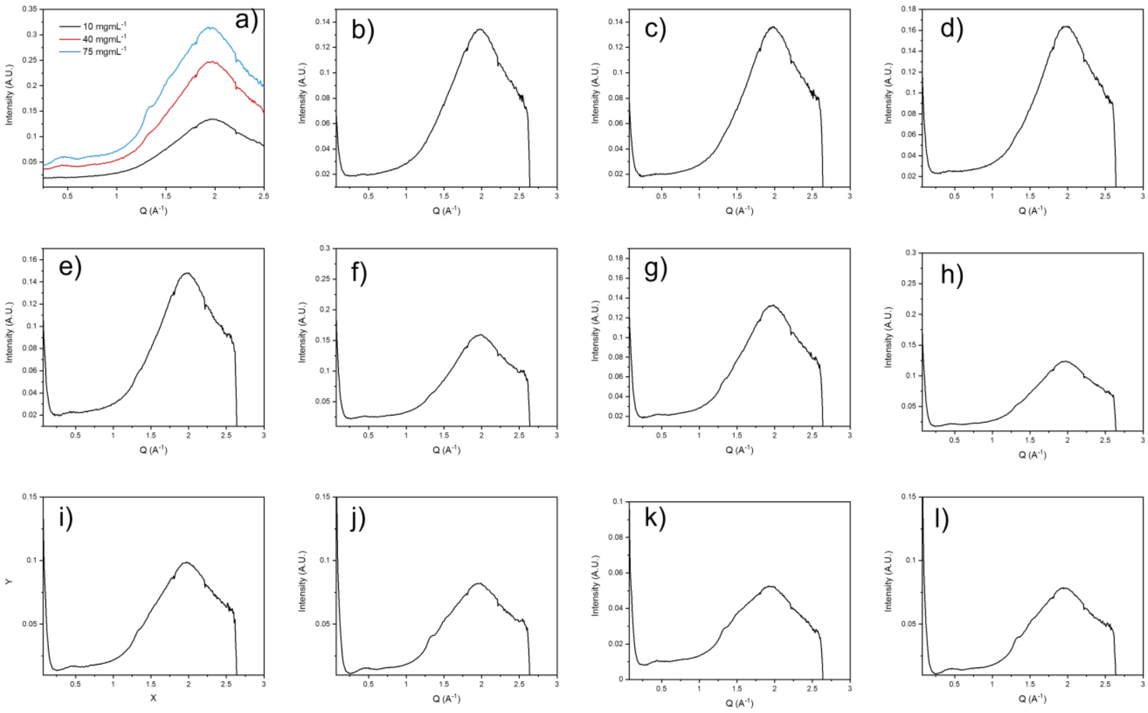

Figure S14. Plot of WAXS data for 2NapFF-TBA; a) 10, 40 and 75 mg/mL plotted (with arbitrary scaling) for comparison; b) 10 mg/mL; c) 15 mg/mL; d) 20 mg/mL; e) 25 mg/mL; f) 30 mg/mL; g) 35 mg/mL; h) 40 mg/mL; i) 45 mg/mL; j) 50 mg/mL; k) 60 mg/mL; l) 75 mg/mL.

Heat cool data

Table S3. Fitting parameters for SAXS data measured pre and post heating for 2NapFF-Na and 2NapFF-TBA.

	**Na – 75 pre heat**	**Na – 75 post heat**	**Na – 10 pre heat**	**Na – 10 post heat**	**TBA – 75 pre heat**	**TBA – 75 post heat**	**TBA – 10 pre heat**	**TBA – 10 post heat**
**Model**	Cylinder	Flexible Cylinder	Flexible Cylinder	Flexible Cylinder	Cylinder	Cylinder	Flexible Cylinder	Cylinder + PL
**Background**	0.0034 ± 0.0002	0.0084 ± 0.0002	0.0084 ± 0.0001	0.013 ± 0.0002	0.02 ± 0.0002	0.008 ± 0.0007	0.0009 ± 2.8×10^{-5}	0.009 ± 0.001
**Scale**	0.005 ± 5.8×10^{-5}	0.0023 ± 0.0008	0.001 ± 3.2×10^{-5}	0.001 ± 5.3×10^{-6}	0.02 ± 0.0008	0.02 ± 0.0002	0.005 ± 0.0003	0.002 0.0003
**Length / Å**	>1000	>1000	>1000	>1000	>1000	>1000	197.1 ± 11.0	>1000
**Kuhn Length / Å**		13.2 ± 6.6	760.5 ± 121.1	281.8 ± 4.5			112.2 ± 11.4	
**Radius / Å**	42.7 ± 0.04	21.2 ± 0.1	43.5 ± 0.1	34.0 ± 0.1	15.3 ± 0.2	16.8 ± 0.2	14.1 ± 0.4	14.1 ± 0.2
**Power Law Scale**								1.3e-8 ± 5.4e-9
**Power Law**								4.0 ± 0.09
χ^2	8.17	4.65	2.15	2.98	2.50	1.96	2.33	1.59

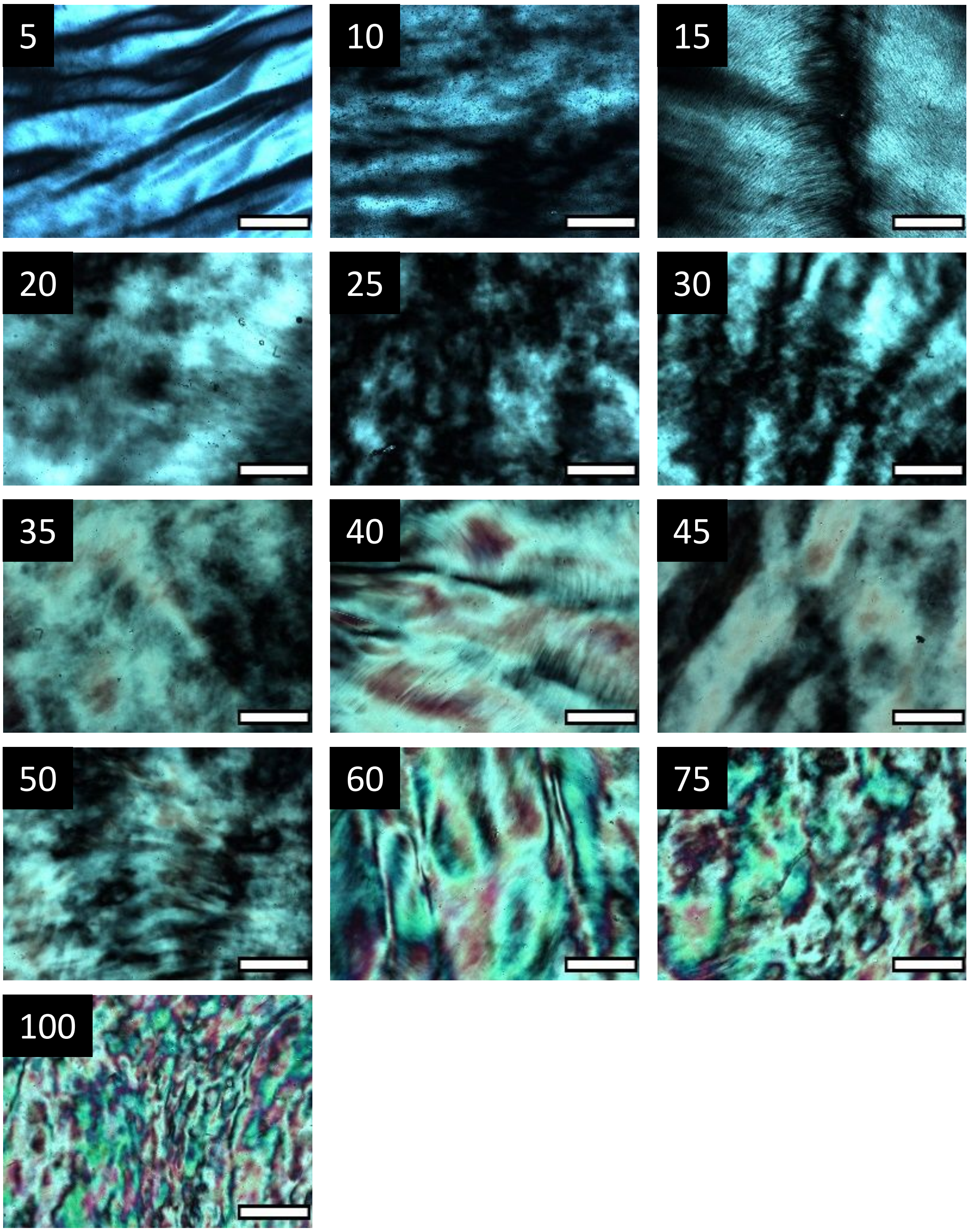

Figure S15. POM images for 2NapFF solutions (made with NaOH) at concentrations ranging from 5 mg/mL to 100 mg/mL. Images were taken at 5x magnification under cross-polarised light. Scale bars represent 500 μm.

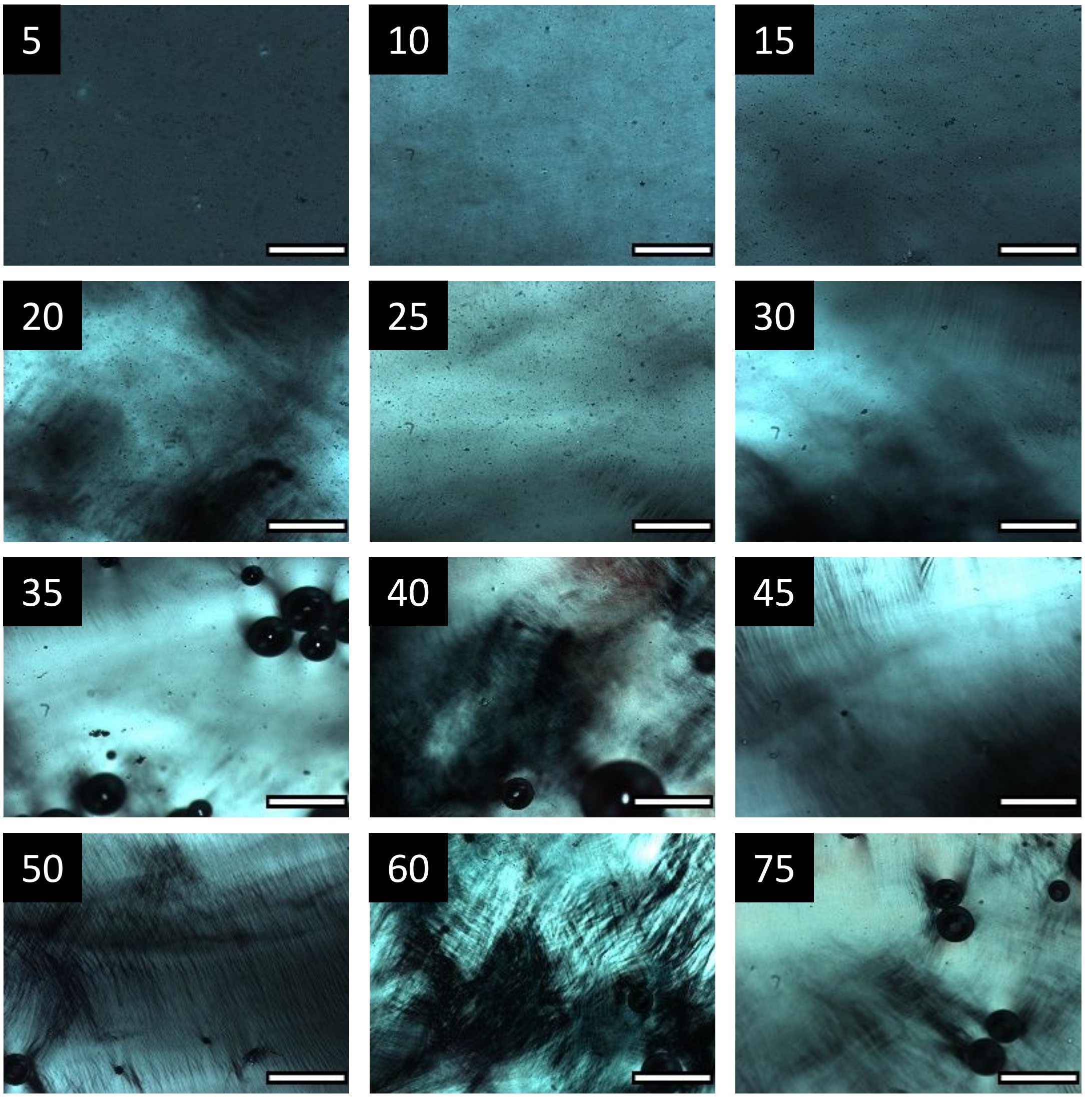

Figure S16. POM images for 2NapFF solutions (made with TBAOH) at concentrations ranging from 5 mg/mL to 75 mg/mL. Images were taken at 5x magnification under cross-polarised light. Scale bars represent 500 μm.

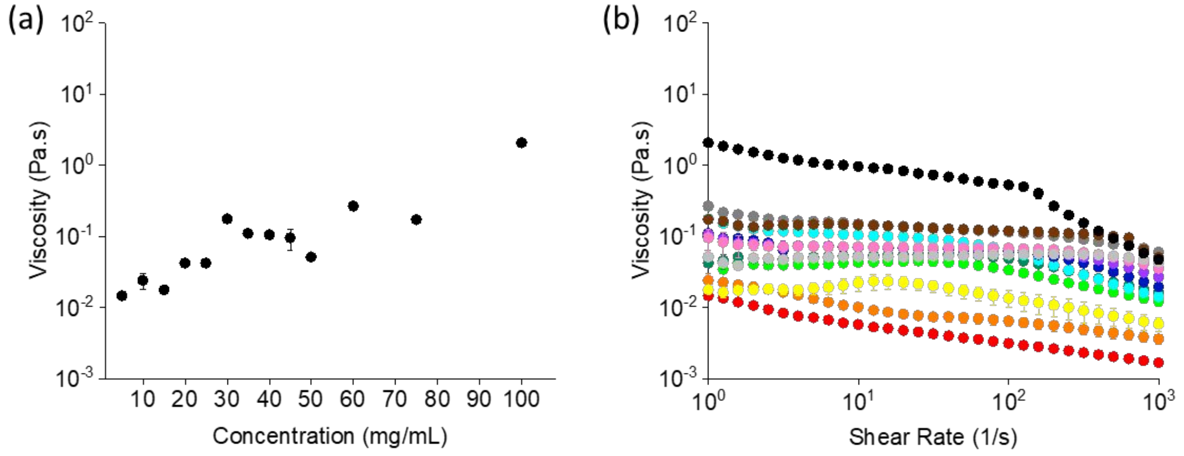

Figure S17. (a) Summary of 2NapFF-Na viscosity at concentrations of 5-100 mg/mL taken at 1 s^{-1}. (b) Full viscosity data for 2NapFF-Na at concentrations of 5 (red); 10 (orange); 15 (yellow); 20 (light green); 25 (dark green); 30 (light blue); 35 (dark blue); 40 (purple); 45 (pink); 50 (light grey); 60 (dark grey); 75 (brown); 100 (black) mg/mL of gelator. Error bars represent the standard deviation between measurements, which were carried out in duplicate.

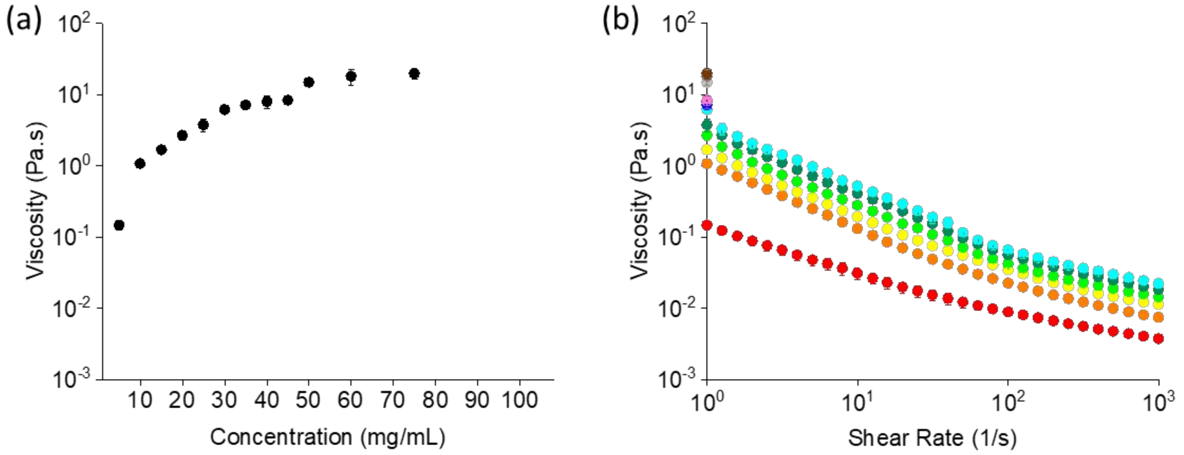

Figure S18. (a) Summary of 2NapFF-TBA viscosity at concentrations of 5-75 mg/mL taken at 1 s^{-1}. (b) Full viscosity data for 2NapFF-TBA at concentrations of 5 (red); 10 (orange); 15 (yellow); 20 (light green); 25 (dark green); 30 (light blue); 35 (dark blue); 40 (purple); 45 (pink); 50 (light grey); 60 (dark grey); 75 (brown) mg/mL of gelator. Error bars represent the standard deviation between measurements, which were carried out in duplicate First data points are shown only for 2NapFF concentrations of 35 mg/mL and greater because at these concentrations, the samples climbed on to the top of the geometry during rotation.

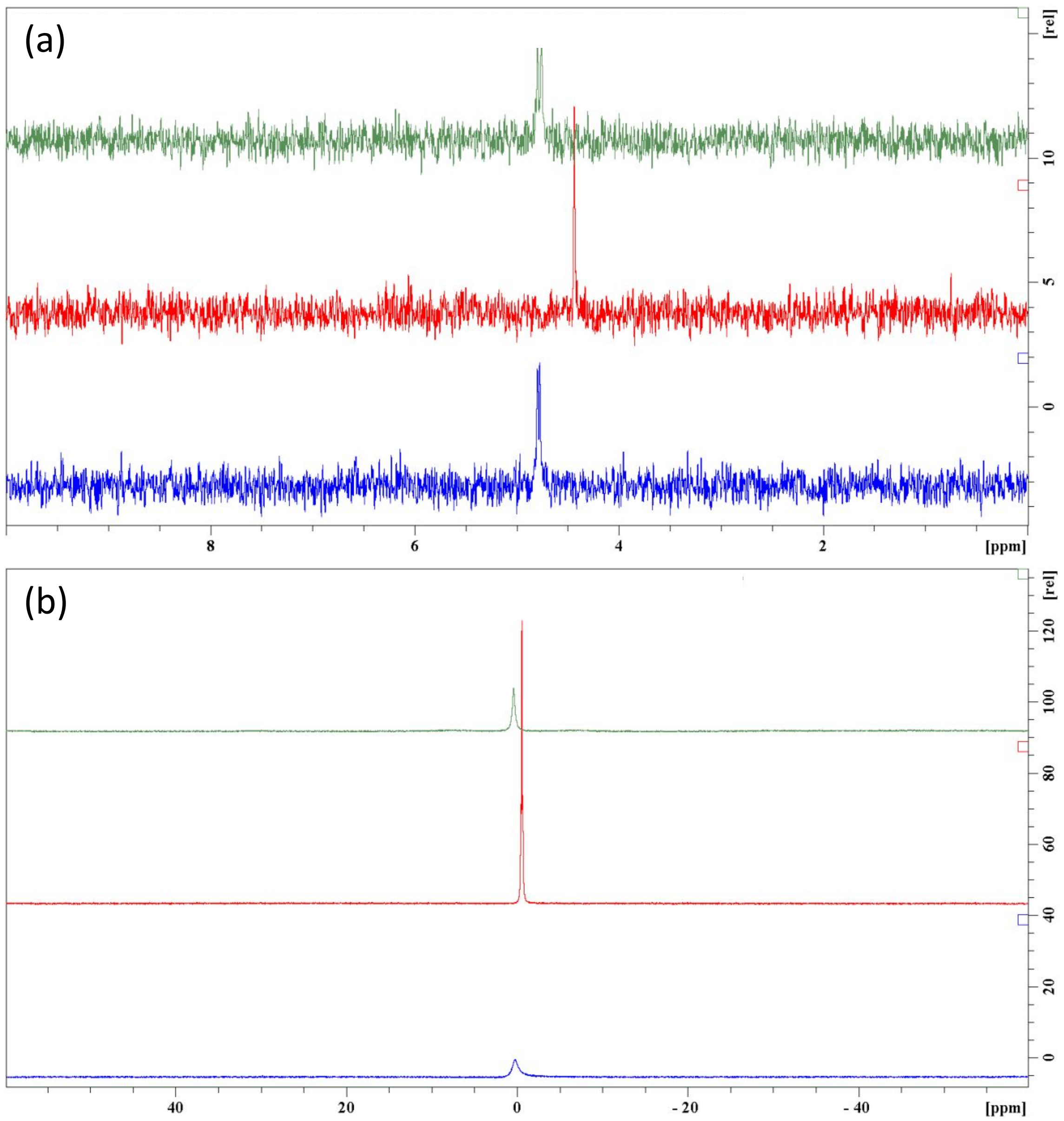

Figure S19. (a) ^{2}H and (b) ^{23}Na heat-cool NMR spectroscopy data for 10 mg/mL 2NapFF solution made with NaOH. Green data was collected at 25 °C, before heating to 60 °C (red) and cooling to 25 °C (blue).

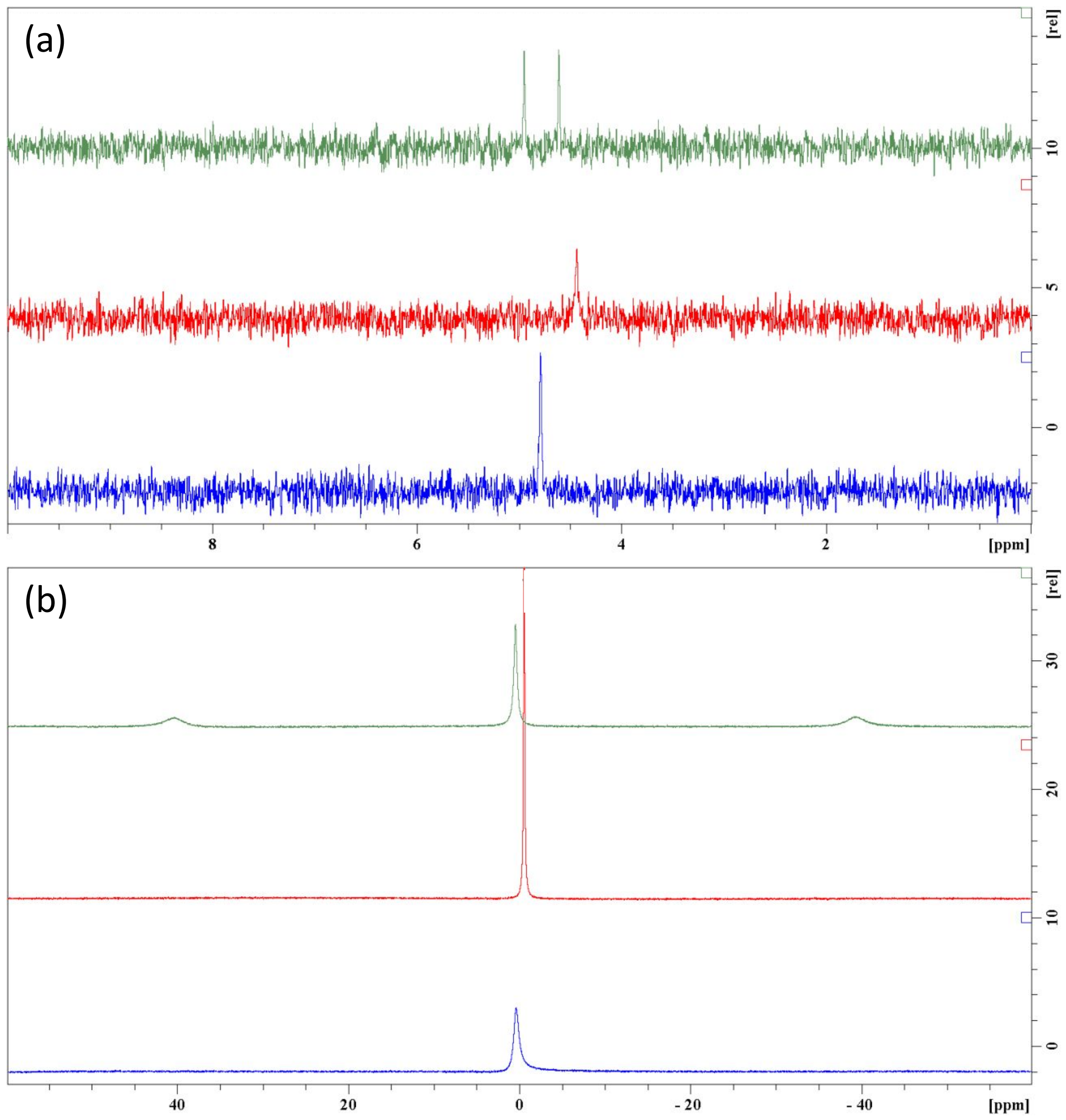

Figure S20. (a) ^{2}H and (b) ^{23}Na heat-cool NMR spectroscopy data for 25 mg/mL 2NapFF solution made with NaOH. Green data was collected at 25 °C, before heating to 60 °C (red) and cooling to 25 °C (blue).

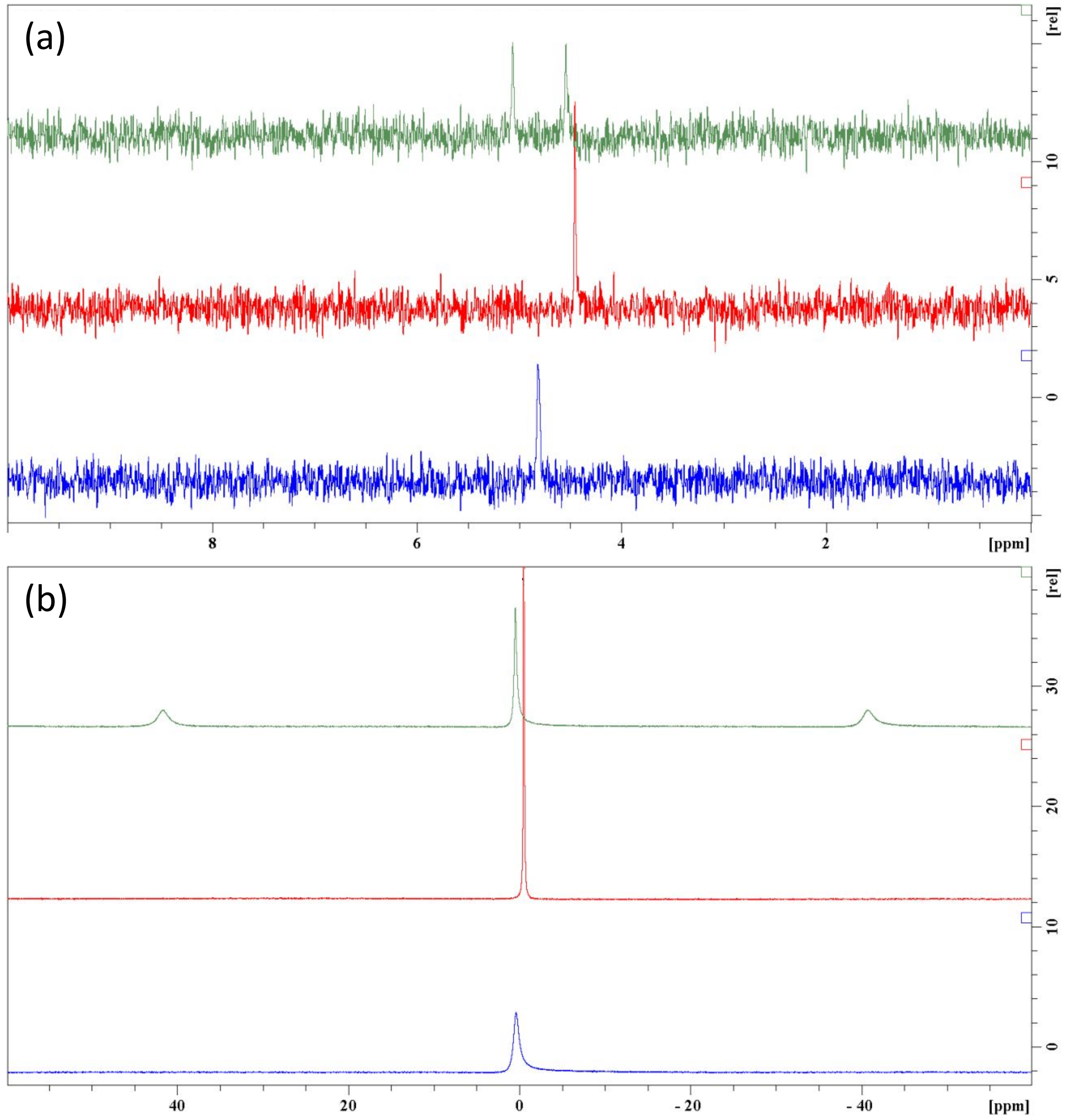

Figure S21. (a) ^{2}H and (b) ^{23}Na heat-cool NMR spectroscopy data for 40 mg/mL 2NapFF solution made with NaOH. Green data was collected at 25 °C, before heating to 60 °C (red) and cooling to 25 °C (blue).

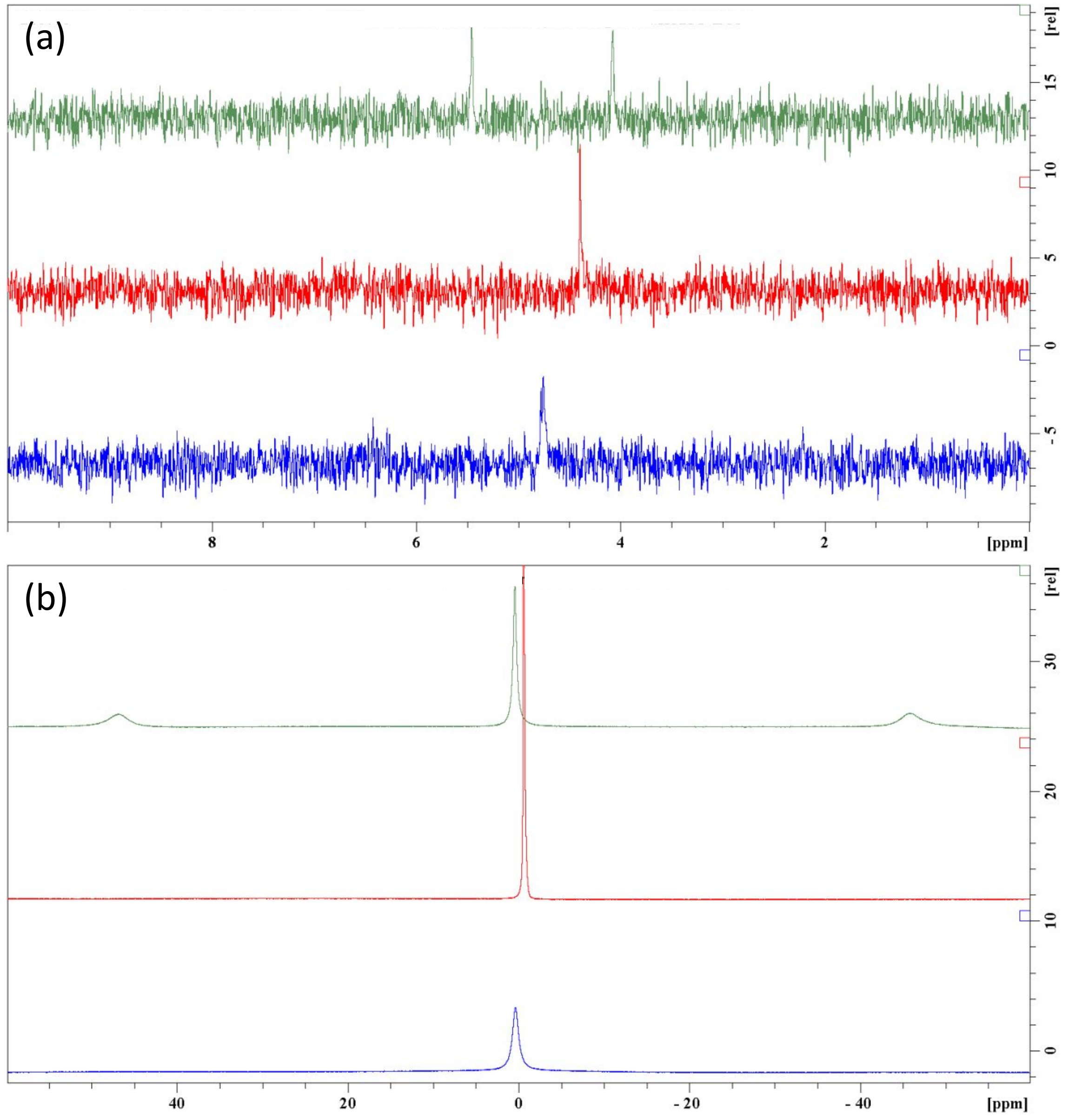

Figure S22. (a) ^{2}H and (b) ^{23}Na heat-cool NMR spectroscopy data for 75 mg/mL 2NapFF solution made with NaOH. Green data was collected at 25 °C, before heating to 60 °C (red) and cooling to 25 °C (blue).

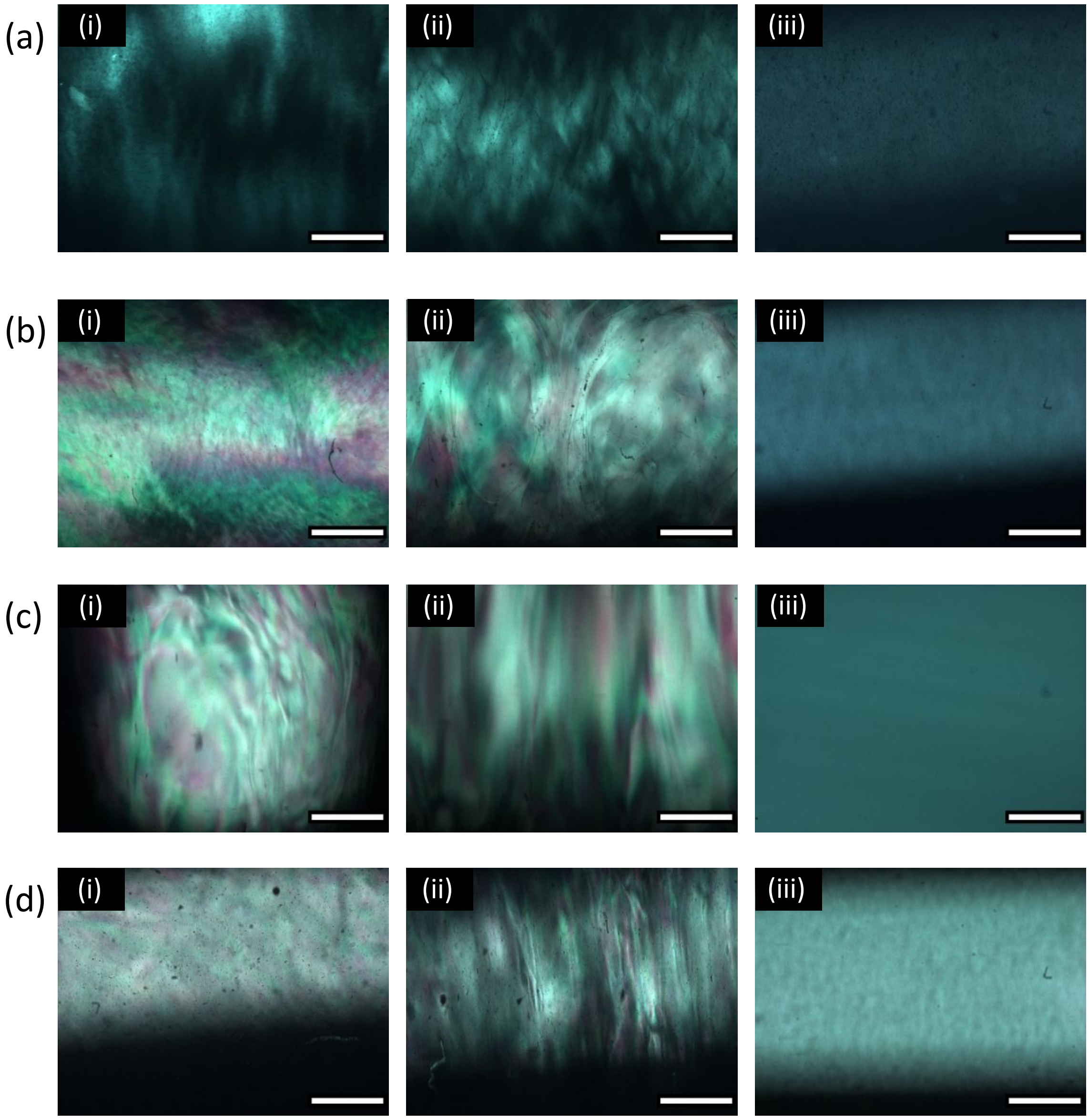

Figure S23. POM images of 2NapFF-Na at exemplar concentrations of (a) 10 mg/mL; (b) 25 mg/mL; (c) 40 mg/mL; (d) 75 mg/mL taken (i) before inserting the sample into the NMR spectrometer; (ii) after inserting the sample into the NMR spectrometer; (iii) after heating the sample to 60 °C and cooling back to 25 °C inside the NMR spectrometer. Images taken under cross-polarised light. Scale bars represent 500 μm.

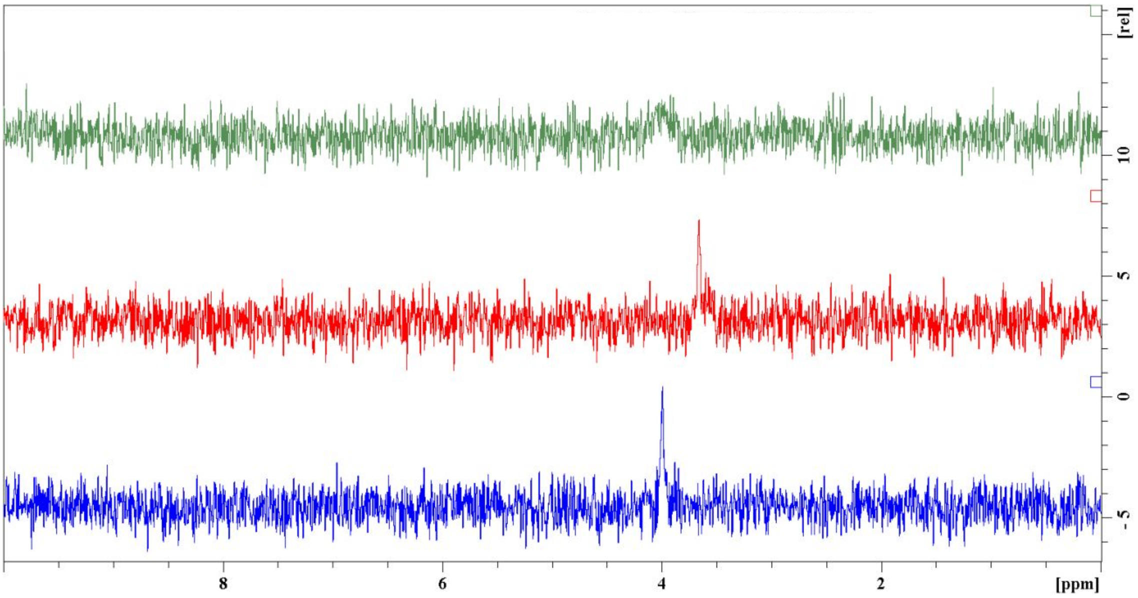

Figure S24. ^{2}H heat-cool NMR spectroscopy data for 10 mg/mL 2NapFF solution made with TBAOH. Green data was collected at 25 °C, before heating to 60 °C (red) and cooling to 25 °C (blue).

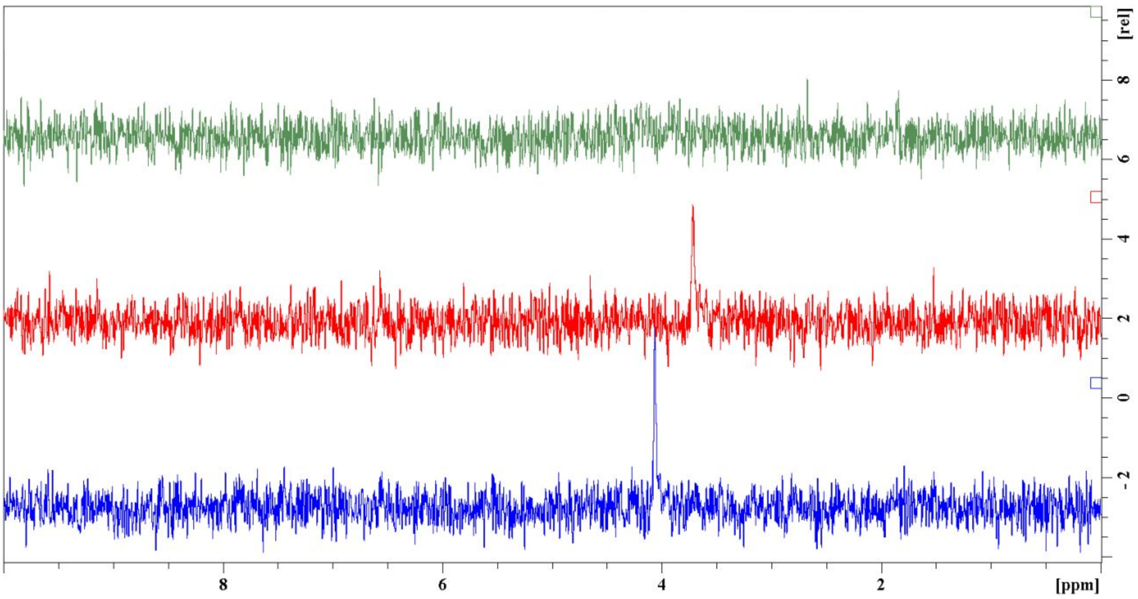

Figure S25. ^{2}H heat-cool NMR spectroscopy data for 25 mg/mL 2NapFF solution made with TBAOH. Green data was collected at 25 °C, before heating to 60 °C (red) and cooling to 25 °C (blue).

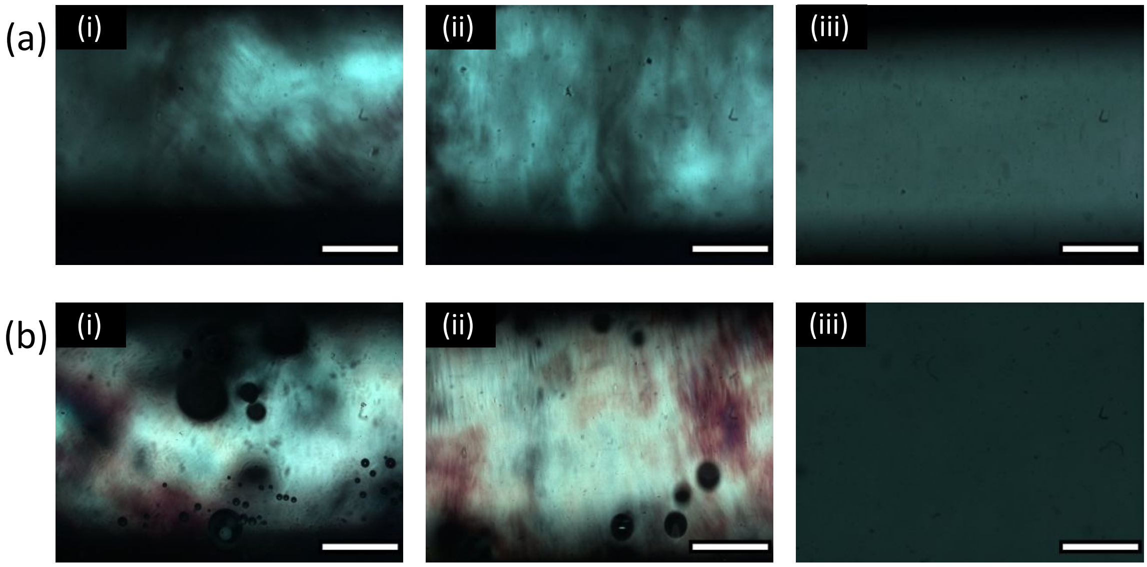

Figure S26. POM images of 2NapFF-TBA at exemplar concentrations of (a) 10 mg/mL; (b) 25 mg/mL; taken (i) before inserting the sample into the NMR spectrometer; (ii) after inserting the sample into the NMR spectrometer; (iii) after heating the sample to 60 °C and cooling back to 25 °C inside the NMR spectrometer. Images taken under cross-polarised light. Scale bars represent 500 μm.

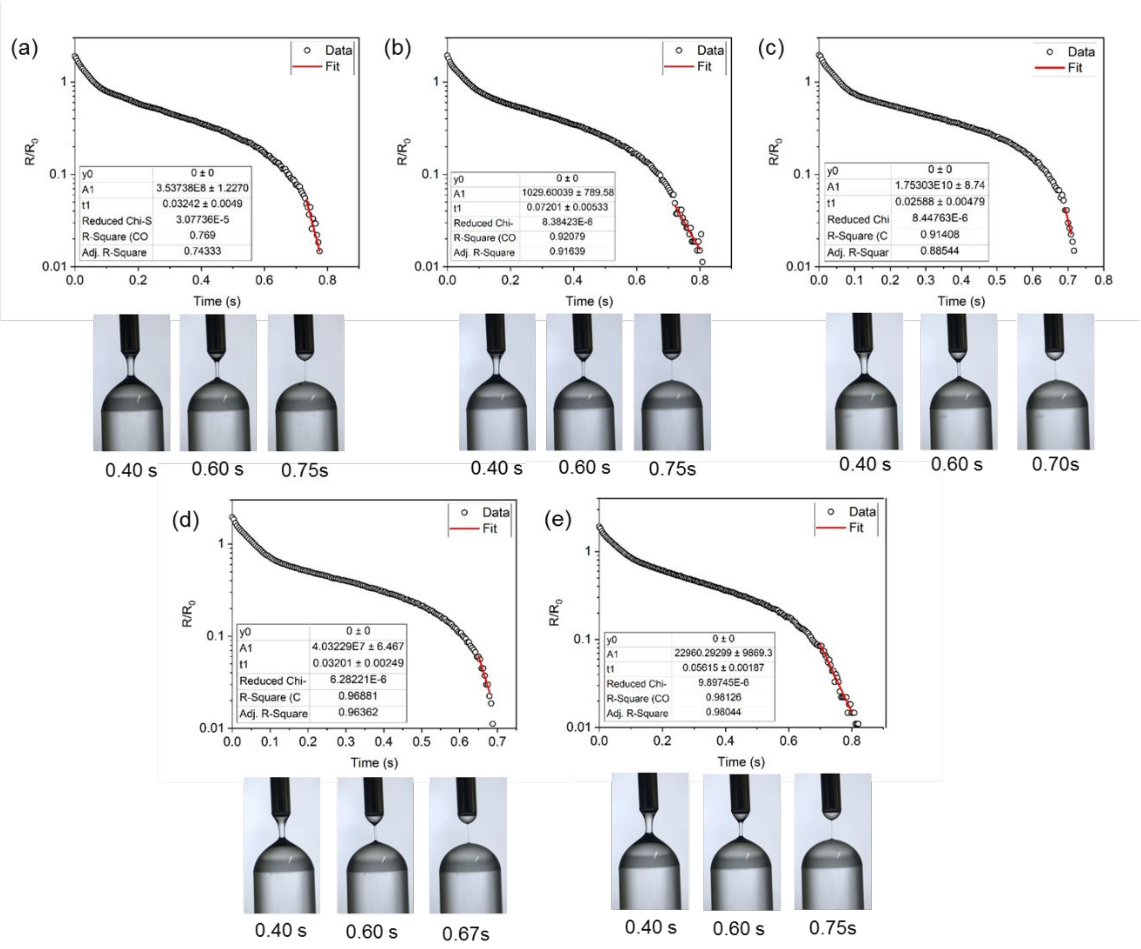

Figure S27. 25 mg/mL 2NapFF-Na DoS data with corresponding fits to the slender filament region with 5 repeats: (a) R1; (b) R2; (c) R3; (d) R4; (e) R5. Each graph has accompanying images of the filament with timestamps.

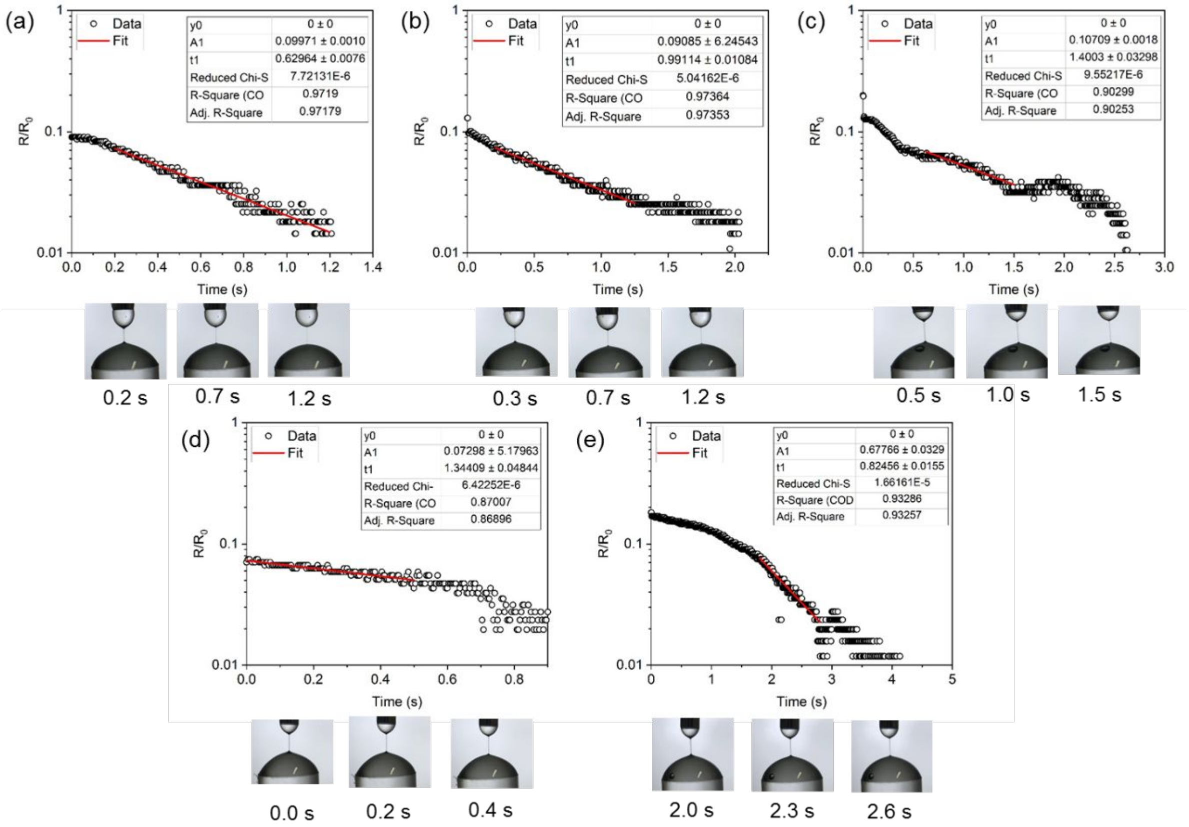

Figure S28. 25 mg/mL 2NapFF-TBA post-heat/cool DoS data with corresponding fits to the slender filament region with 5 repeats (R). (a) R1; (b) R2; (c) R3; (d) R4; (e) R5. Each graph has accompanying images of the filament with timestamps.

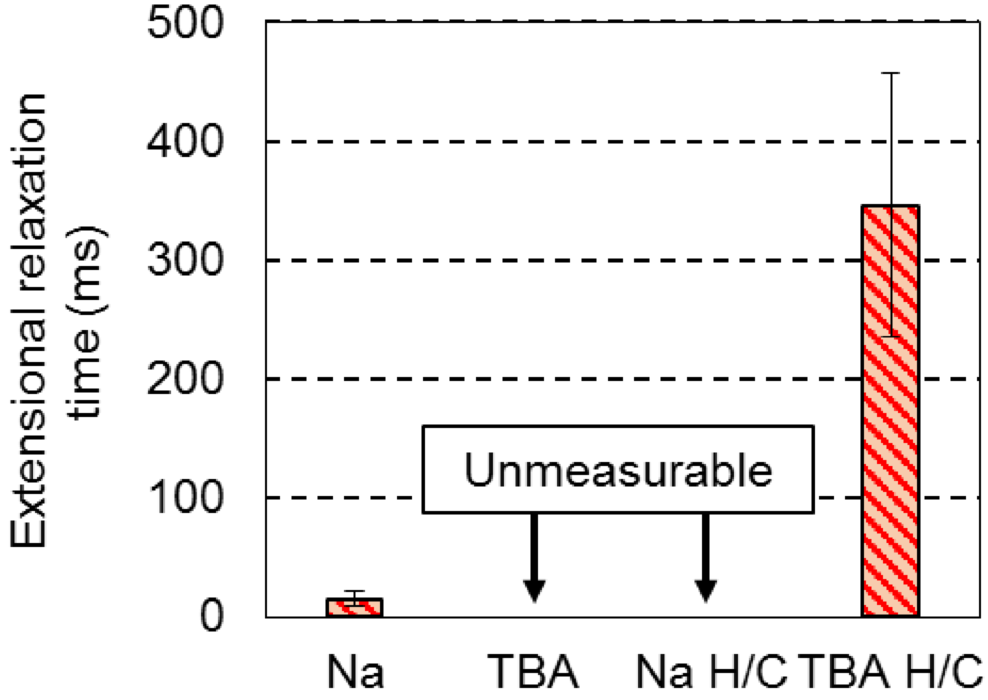

Figure S29. Bar graph showing extensional relaxation times for 25 mg/mL 2NapFF-Na or 2NapFF-TBA before and after heat/cool.

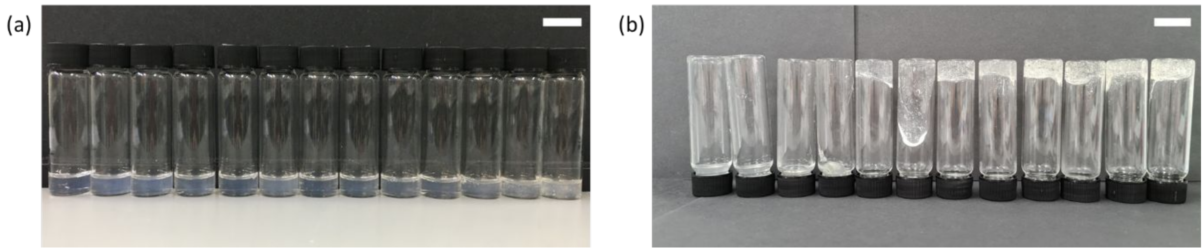

Figure S30. Photographs of (a) 2NapFF-Na at concentrations of 5-100 mg/mL (left-right) and (b) 2NapFF-TBA at concentrations of 5-75 mg/mL (left-right). No samples of 2NapFF-Na at any concentration are invertible, however 2NapFF-TBA from concentrations of 25 mg/mL and greater are able to support themselves for at least one minute. Scale bars represent 2 cm.

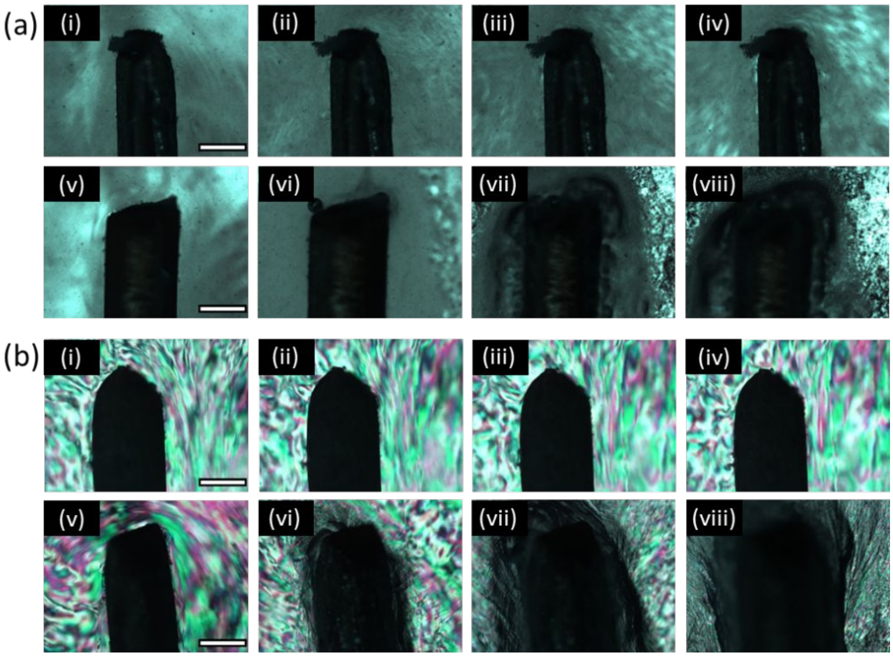

Figure S31. Heating of solutions of 2NapFF made using NaOH at concentrations (a) 10 mg/mL and (b) 75 mg/mL using a copper wire. (i) Control at 0 minutes; (ii) control at 10 minutes; (iii) control at 20 minutes; (iv) control at 30 minutes. (v) Before heating copper wire; (vi) after heating copper wire for 10 minutes; (vii) after heating copper wire for 20 minutes; (viii) after heating copper wire for 30 minutes. Images are taken under cross-polarised light. Scale bar represents 500 μm.

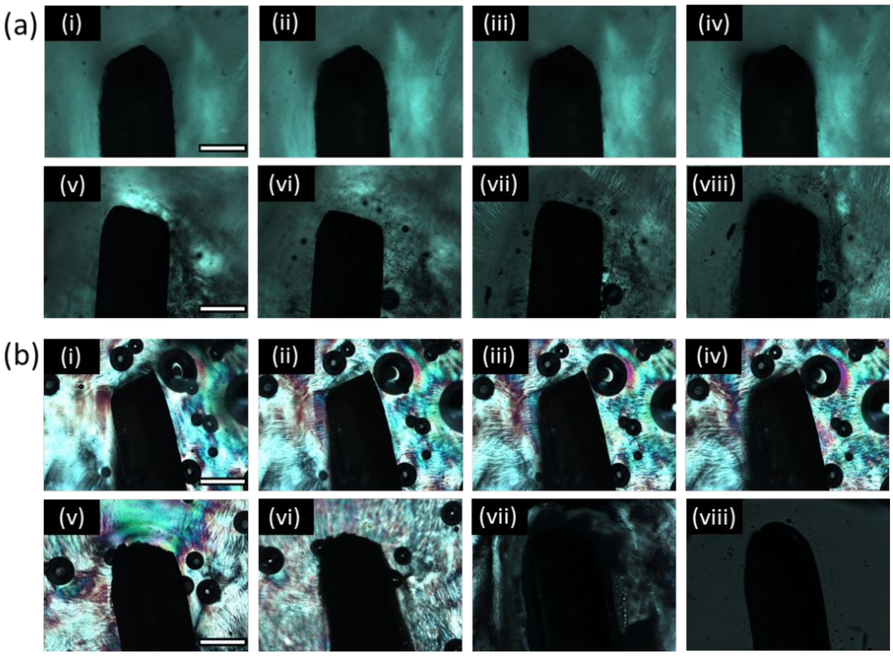

Figure S32. Heating of solutions of 2NapFF made using TBAOH at concentrations (a) 10 mg/mL and (b) 75 mg/mL using a copper wire. (i) Control at 0 minutes; (ii) control at 10 minutes; (iii) control at 20 minutes; (iv) control at 30 minutes. (v) Before heating copper wire; (vi) after heating copper wire for 10 minutes; (vii) after heating copper wire for 20 minutes; (viii) after heating copper wire for 30 minutes. Images are taken under cross-polarised light. Scale bar represents 500 μm.

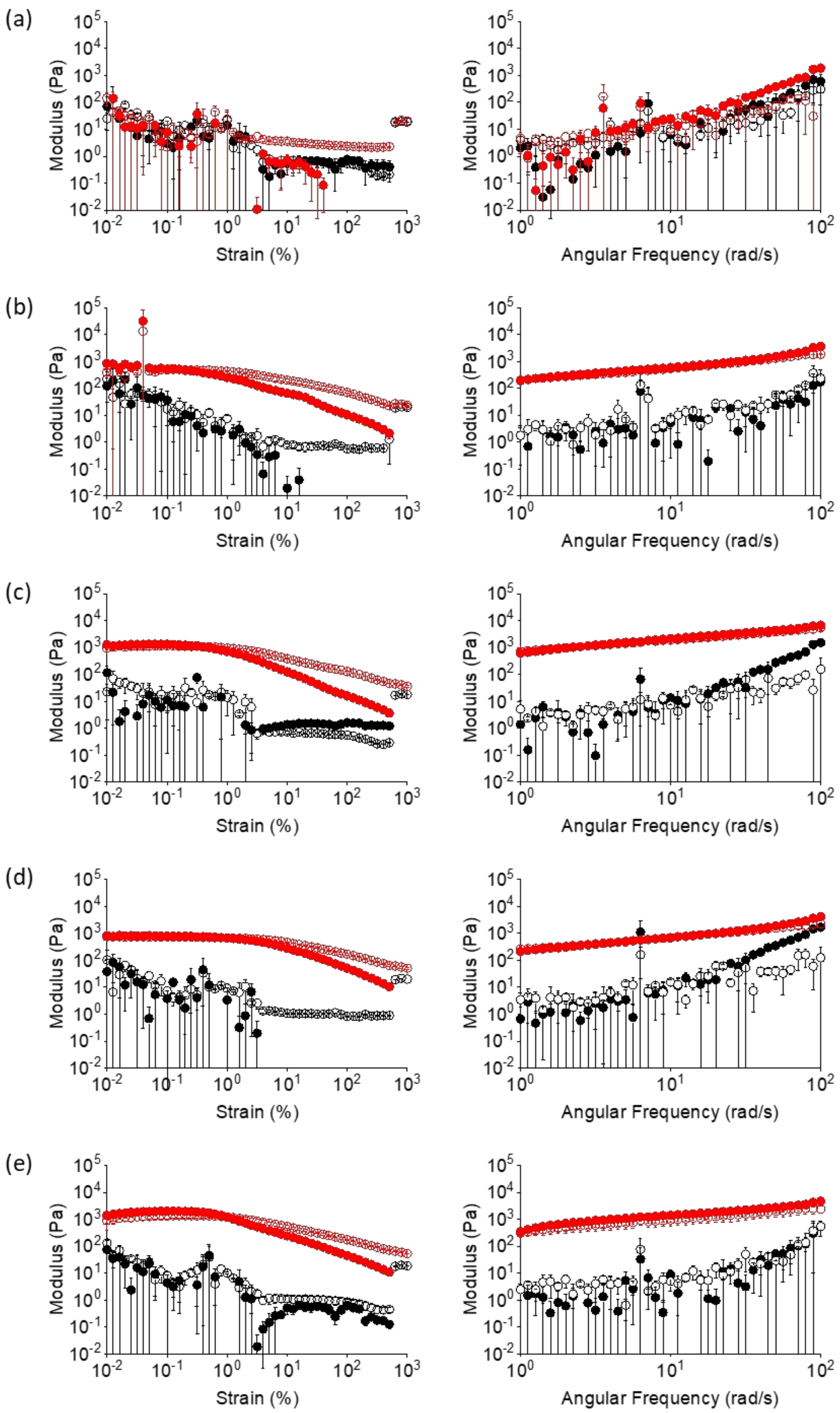

Figure S33. Full strain (left) and frequency (right) data for 2NapFF-Na solutions of concentration (a) 5 mg/mL; (b) 10 mg/mL; (c) 15 mg/mL; (d) 20 mg/mL; (e) 25 mg/mL. Black data represents measurements performed at room temperature and red data represents measurements performed at room temperature 2 hours after a heat-cool cycle to 60 ^{0}C. G′ is represented by filled circles and G″ by empty circles. Error bars represent the standard deviation between measurements, which were carried out in triplicate.

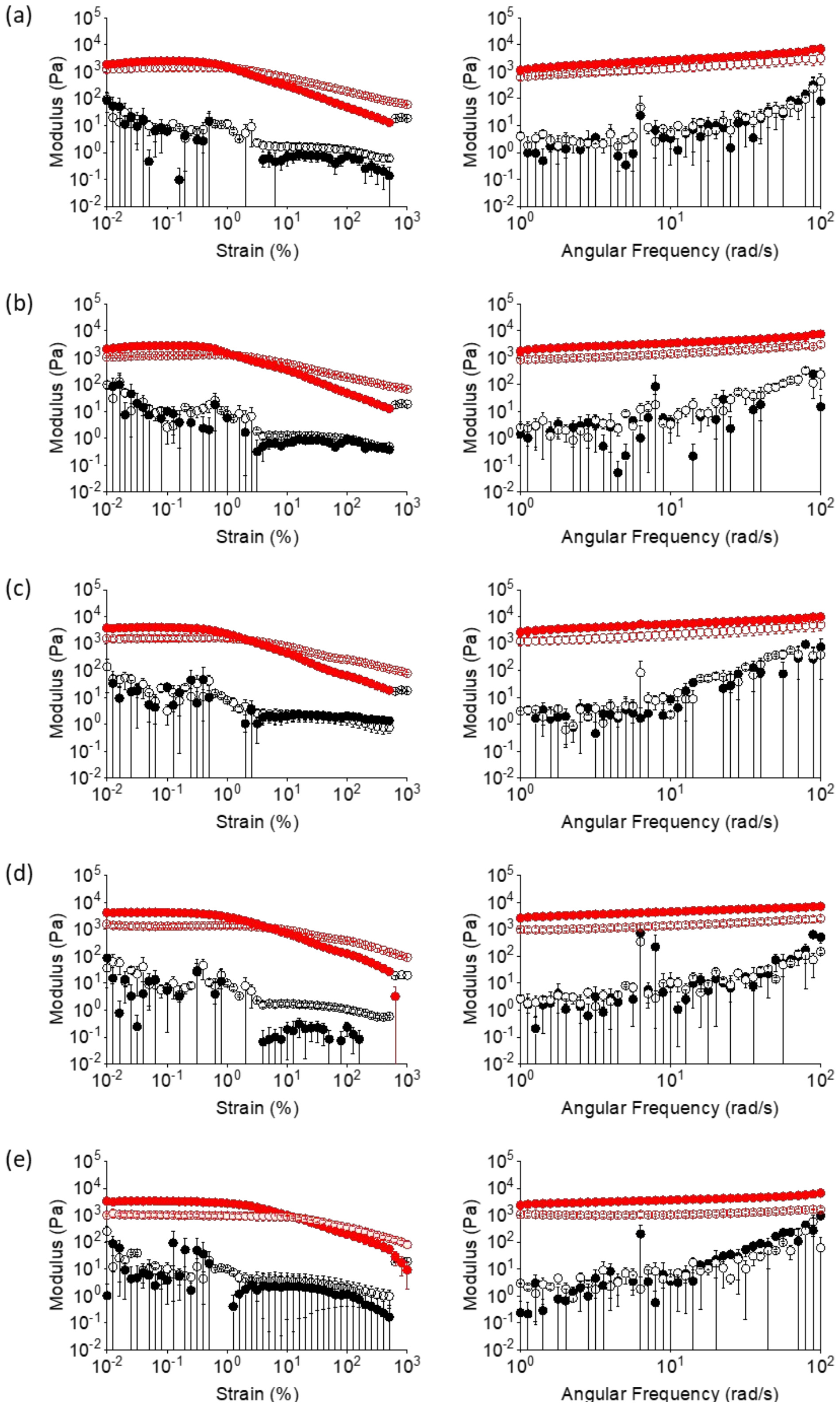

Figure S34. Full strain (left) and frequency (right) data for 2NapFF-Na solutions of concentration (a) 30 mg/mL; (b) 35 mg/mL; (c) 40 mg/mL; (d) 45 mg/mL; (e) 50 mg/mL. Black data represents measurements performed at room temperature and red data represents measurements performed at room temperature 2 hours after a heat-cool cycle to 60 ^{0}C. G′ is represented by filled circles and G″ by empty circles. Error bars represent the standard deviation between measurements, which were carried out in triplicate.

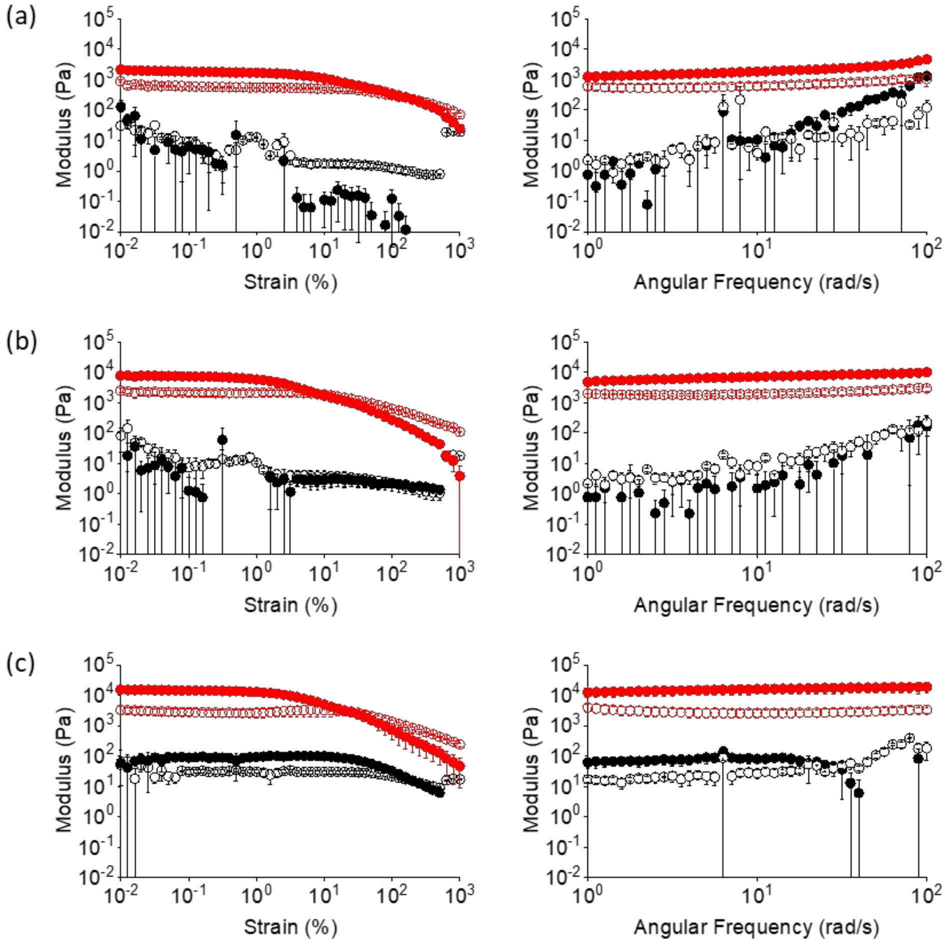

Figure S35. Full strain (left) and frequency (right) data for 2NapFF-Na solutions of concentration (a) 60 mg/mL; (b) 75 mg/mL; (c) 100 mg/mL. Black data represents measurements performed at room temperature and red data represents measurements performed at room temperature 2 hours after a heat-cool cycle to 60 ^{0}C. G′ is represented by filled circles and G″ by empty circles. Error bars represent the standard deviation between measurements, which were carried out in triplicate.

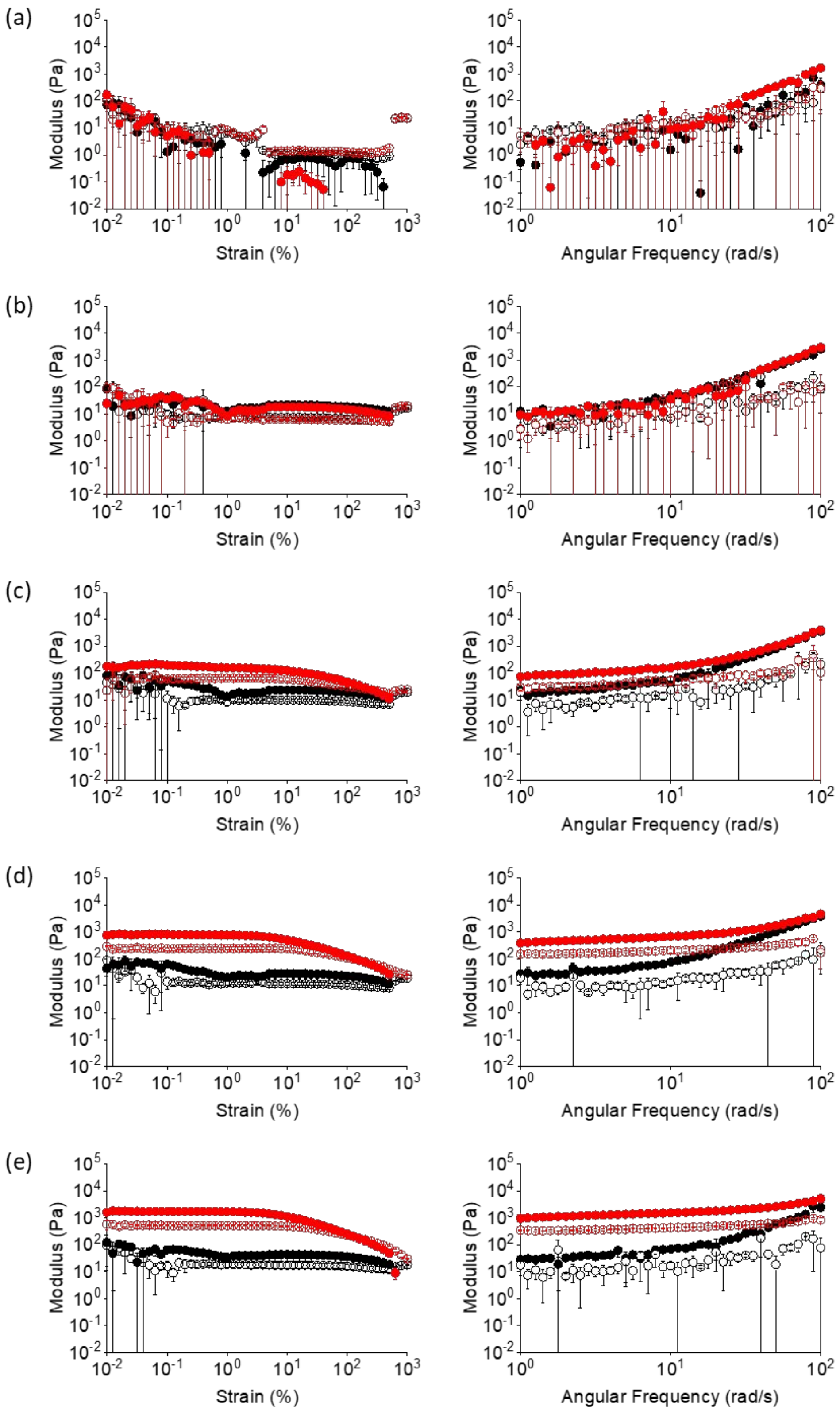

Figure S36. Full strain (left) and frequency (right) data for 2NapFF-TBA solutions of concentration (a) 5 mg/mL; (b) 10 mg/mL; (c) 15 mg/mL; (d) 20 mg/mL; (e) 25 mg/mL. Black data represents measurements performed at room temperature and red data represents measurements performed at room temperature 2 hours after a heat-cool cycle to 60 ^{0}C. G′ is represented by filled circles and G″ by empty circles. Error bars represent the standard deviation between measurements, which were carried out in triplicate.

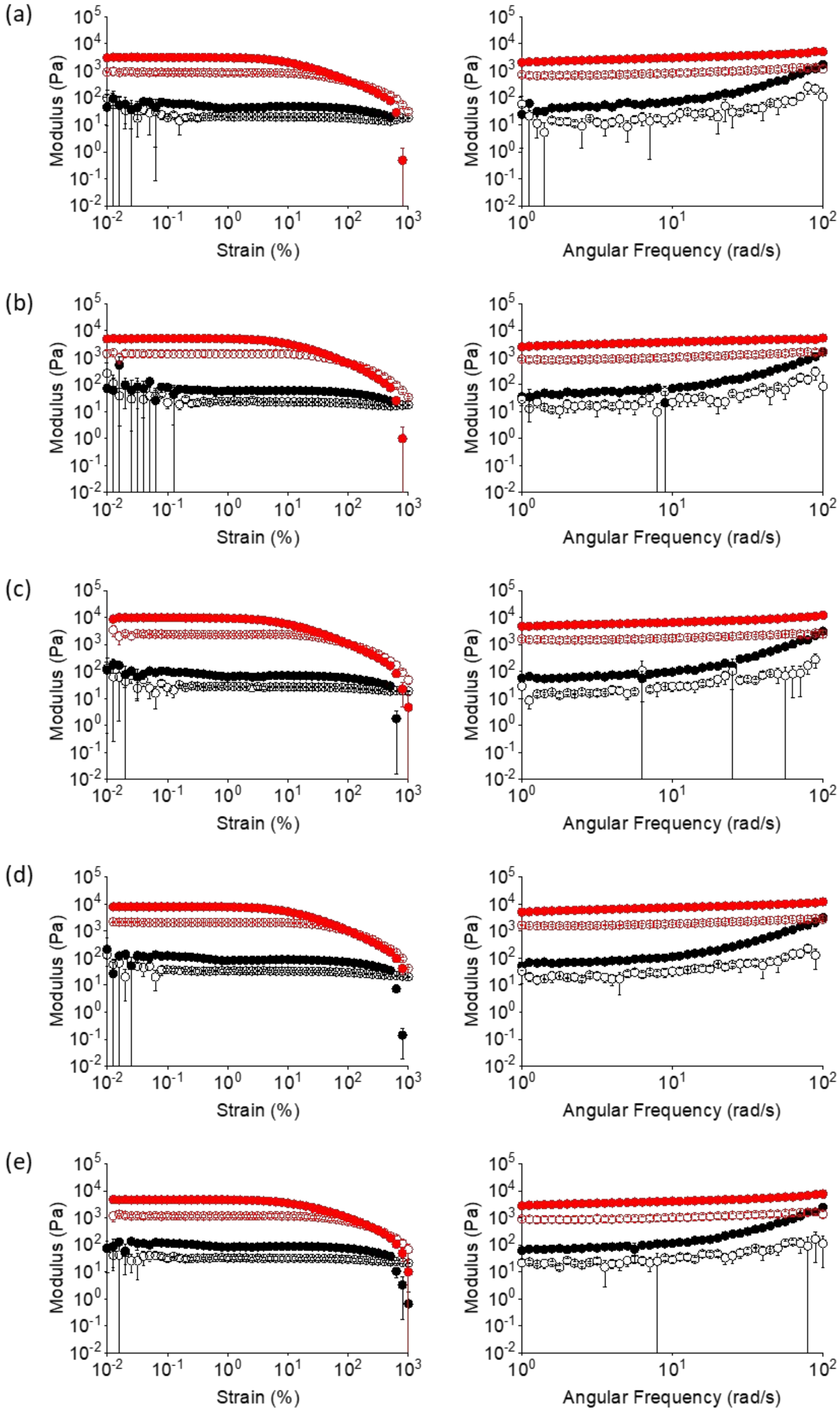

Figure S37. Full strain (left) and frequency (right) data for 2NapFF-TBA solutions of concentration (a) 30 mg/mL; (b) 35 mg/mL; (c) 40 mg/mL; (d) 45 mg/mL; (e) 50 mg/mL. Black data represents measurements performed at room temperature and red data represents measurements performed at room temperature 2 hours after a heat-cool cycle to 60 ^{0}C. G′ is represented by filled circles and G″ by empty circles. Error bars represent the standard deviation between measurements, which were carried out in triplicate.

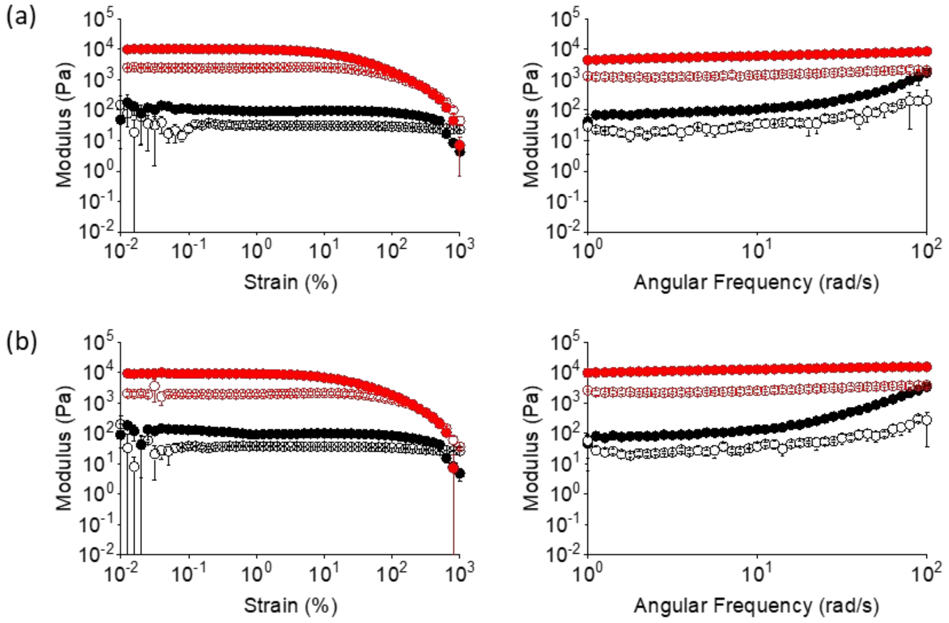

Figure S38. Full strain (left) and frequency (right) data for 2NapFF-TBA solutions of concentration (a) 60 mg/mL; (b) 75 mg/mL. Black data represents measurements performed at room temperature and red data represents measurements performed at room temperature 2 hours after a heat-cool cycle to 60 ^{0}C. G′ is represented by filled circles and G″ by empty circles. Error bars represent the standard deviation between measurements, which were carried out in triplicate.

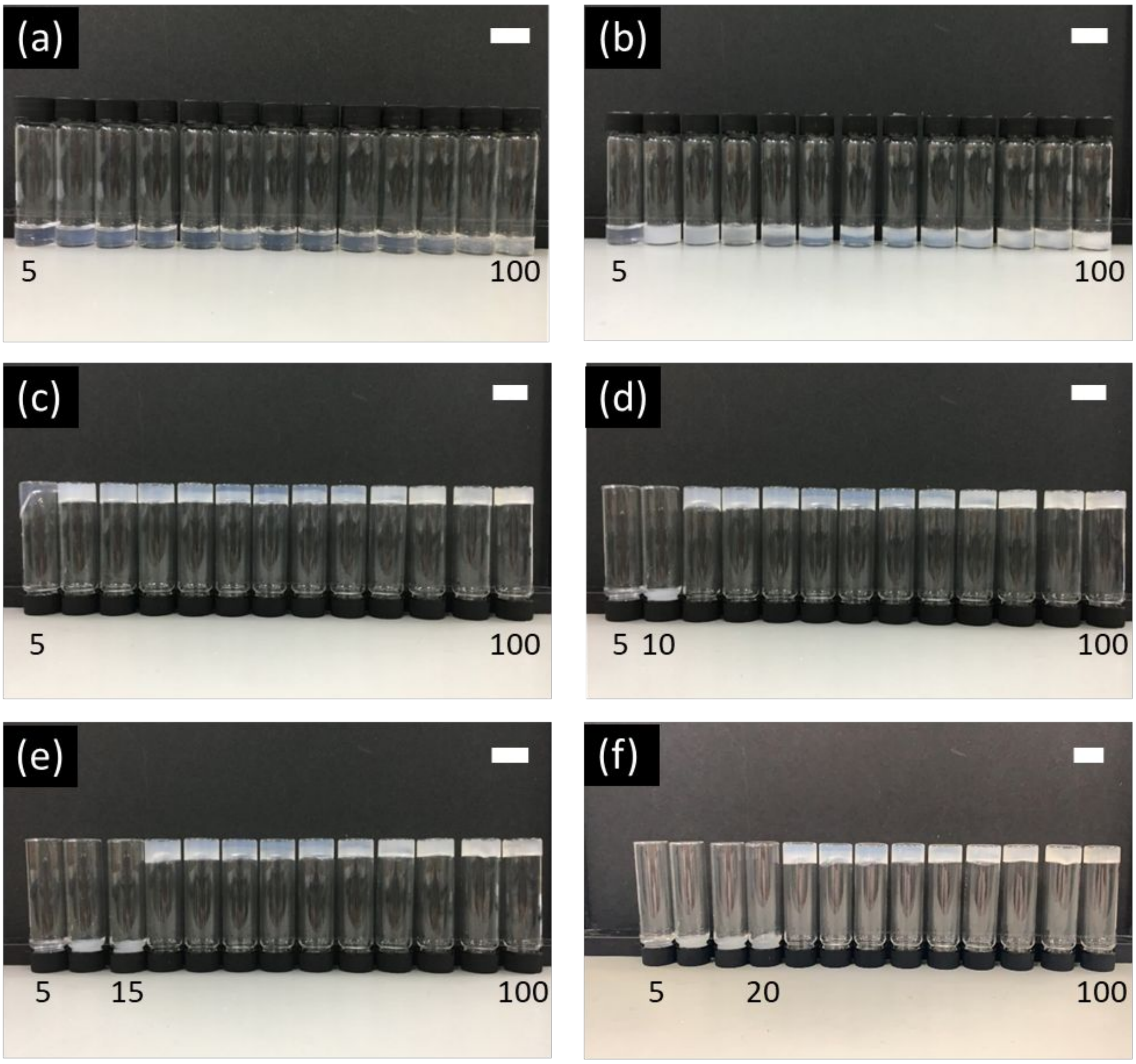

Figure S39. Heat-cool of 2NapFF made with Na (a) before heating; (b) straight after heating in a 60 °C oven for 1 hour; (c) 2 hours after heating; (d) 1 day after heating; (e) 3 days after heating; (f) 9 days after heating. Vials were left undisturbed and inverted for a 14 day period, after which the experiment was stopped due to potential evaporation issues. Scale bars represent 2 cm.

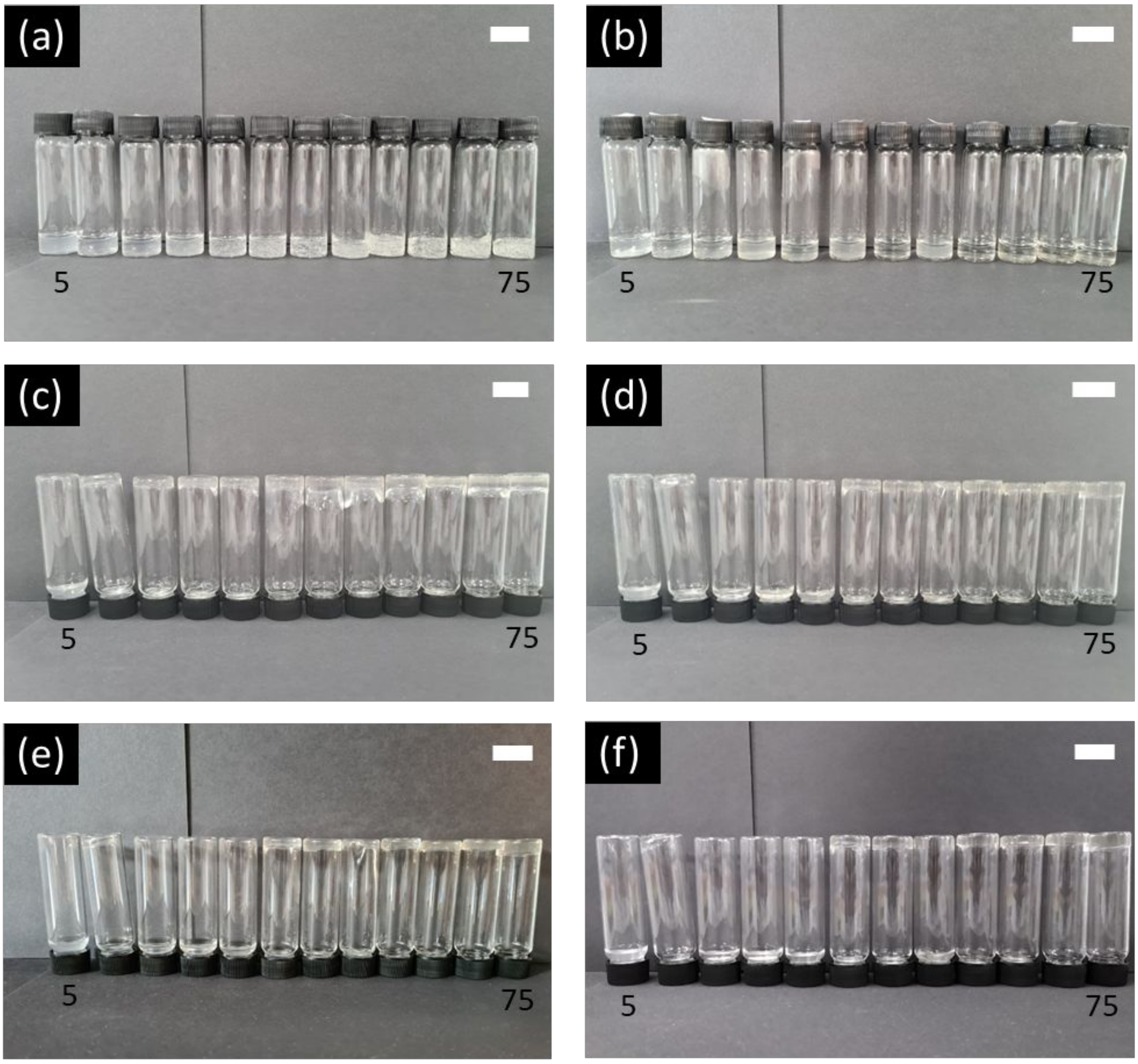

Figure S40. Heat-cool of 2NapFF made with TBA (a) before heating; (b) straight after heating in a 60 °C oven for 1 hour; (c) 2 hours after heating; (d) 1 day after heating; (e) 3 days after heating; (f) 9 days after heating. Vials were left undisturbed and inverted for a 14 day period, after which the experiment was stopped due to potential evaporation issues. Scale bars represent 2 cm.

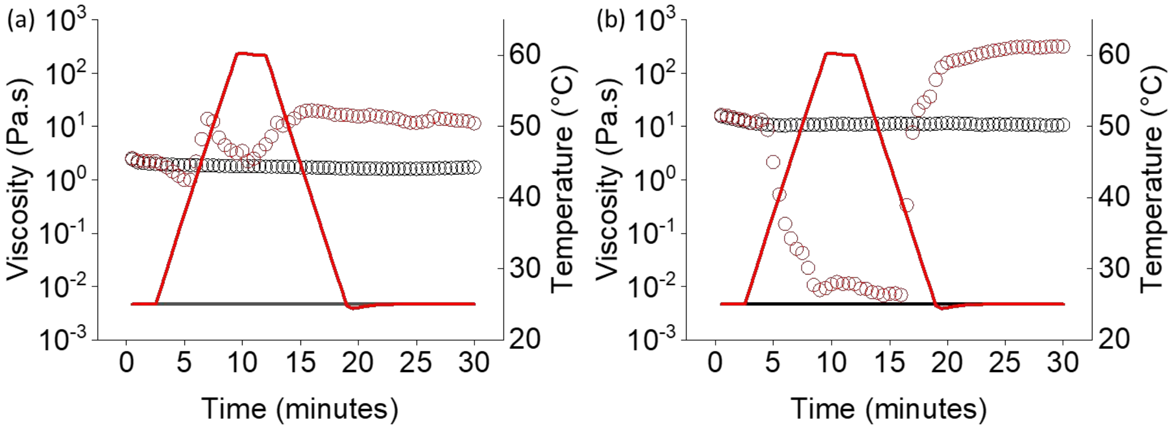

Figure S41. Viscosity data for 2NapFF at a concentration of 75 mg/mL made with (a) Na and (b) TBA during heating using a CP50 geometry and a shear rate fo 1 s^{-1}. Red points show the viscosity being measured for 2 minutes at 25 °C, then heated at a rate of 5 °C/min from 25 °C to 60 °C before being held at 60 °C for 2 minutes. This was then cooled back to 25 °C at the same rate with the viscosity continuously monitored. Black points show a control sample which was kept at 25 °C throughout. Line plots show the temperatures during the heat-cool (red) and control (black) viscosity measurements. Data shown are of single measurements.

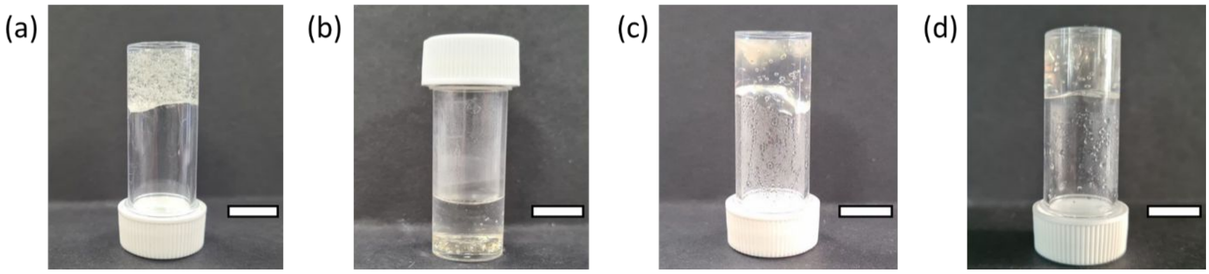

Figure S42. Photographs of 2NapFF-TBA at a concentration of 75 mg/mL taken (a) before heating; (b) straight after 1 hour heating at 60 °C; (c) after 2 hours cooling to room temperature; (d) the day following heating. Scale bars represent 1 cm.

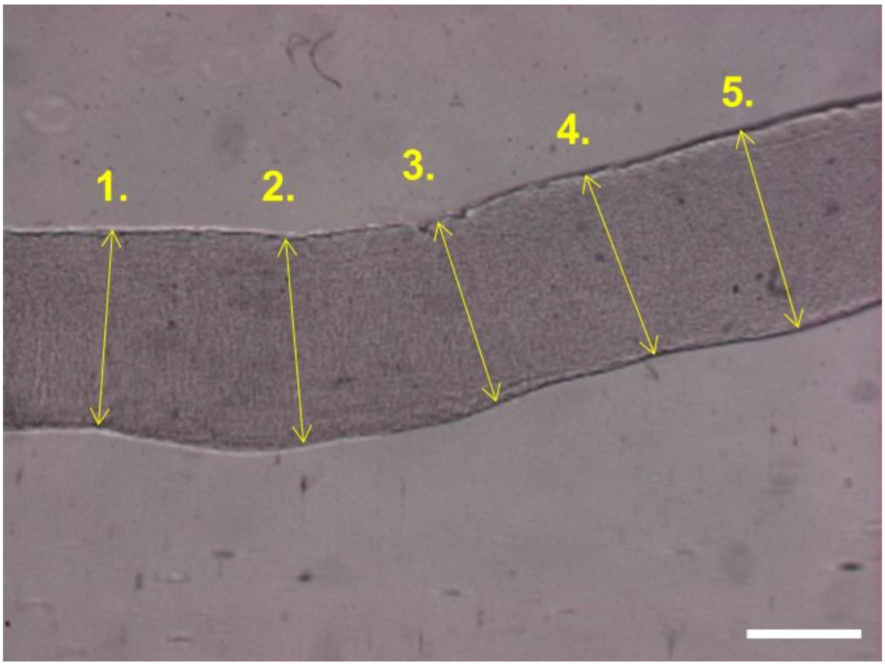

Figure S43. Exemplar image showing how measurements were taken for heat-cool noodles. The diameter was measured at 5 equidistant points along the length of the noodle. Scale bar represents 0.3 mm.

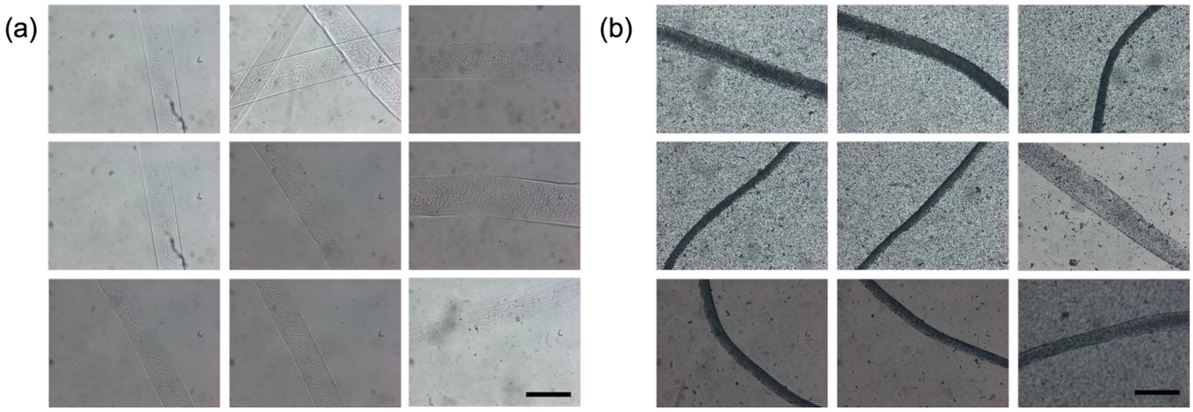

Figure S44. Representative microscope images of 2NapFF-Na gel noodles both (a) before heating and (b) after heating. The black scale bars apply for all images and represents 0.6 mm.

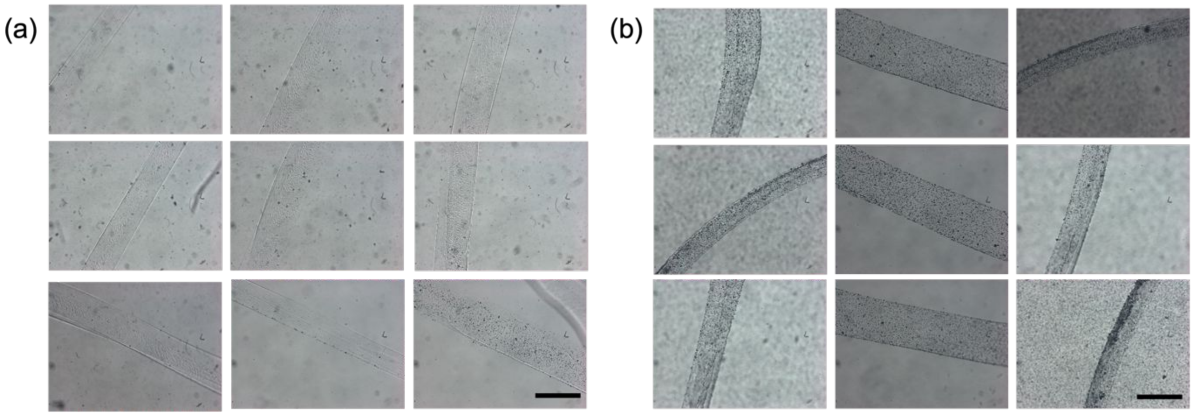

Figure S45. Representative microscope images of 2NapFF-Na gel noodles as a control (a) before and (b) after sitting for 4 hours. The black scale bars apply for all images and represents 0.6 mm.

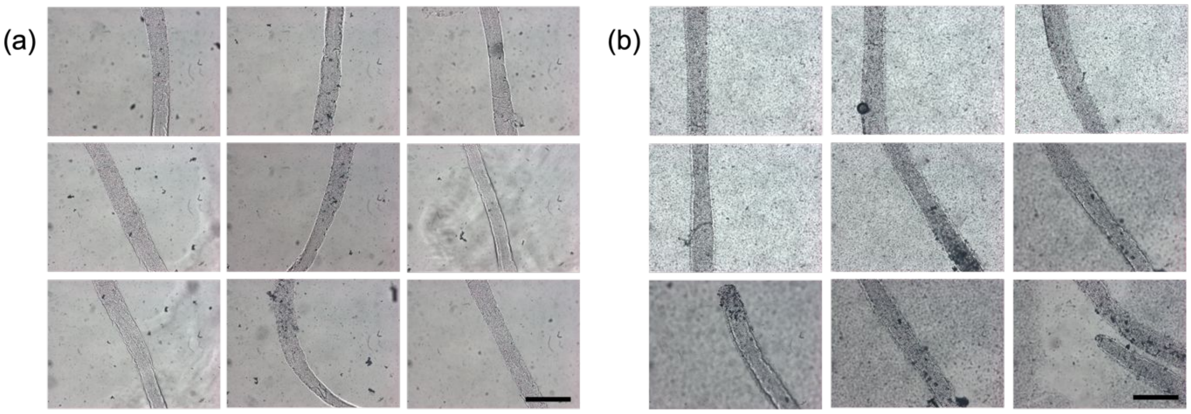

Figure S46. Representative microscope images of 2NapFF-TBA gel noodles as a control (a) before and (b) after sitting. The black scale bars apply for all images and represents 0.6 mm

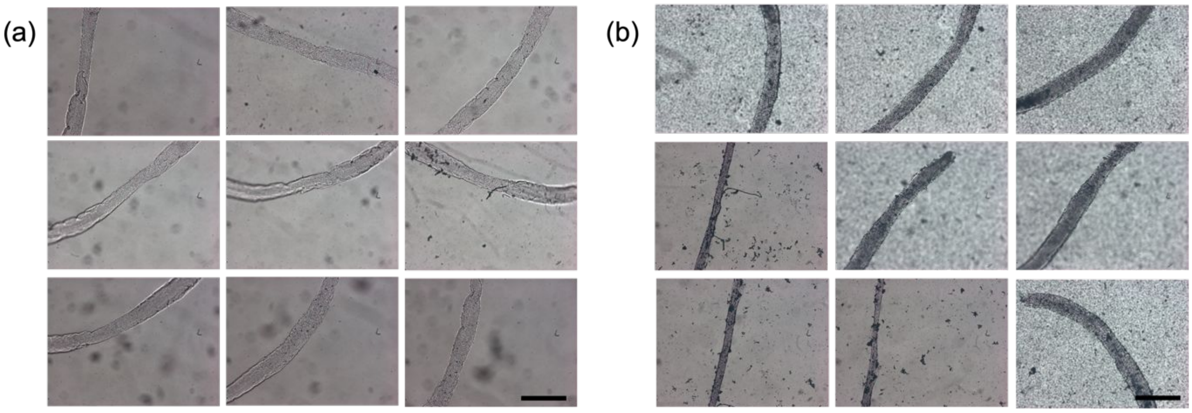

Figure S47. Representative microscope images of 2NapFF-TBA gel noodles as a control (a) before heating and (b) after heating. The black scale bars apply for all images and represents 0.6 mm.

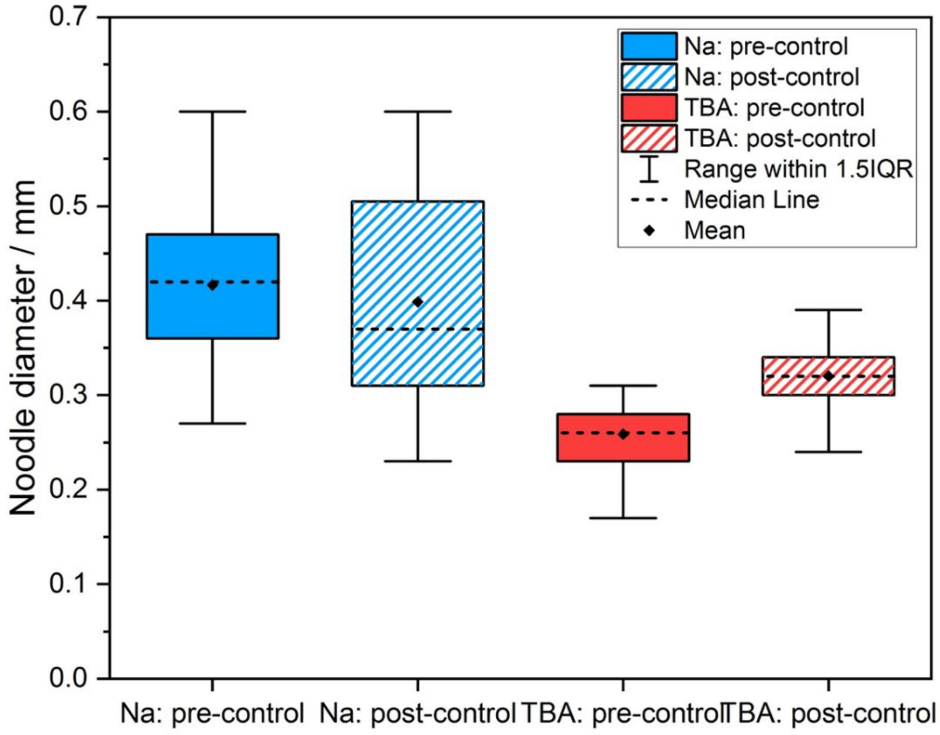

Figure S48. Summary of measurement of diameter of noodles before and after heating. Number of data points: 2NapFF-Na pre-heating (227); post-heating (155). 2NapFF-TBA pre-heating (200); post-heating (180).

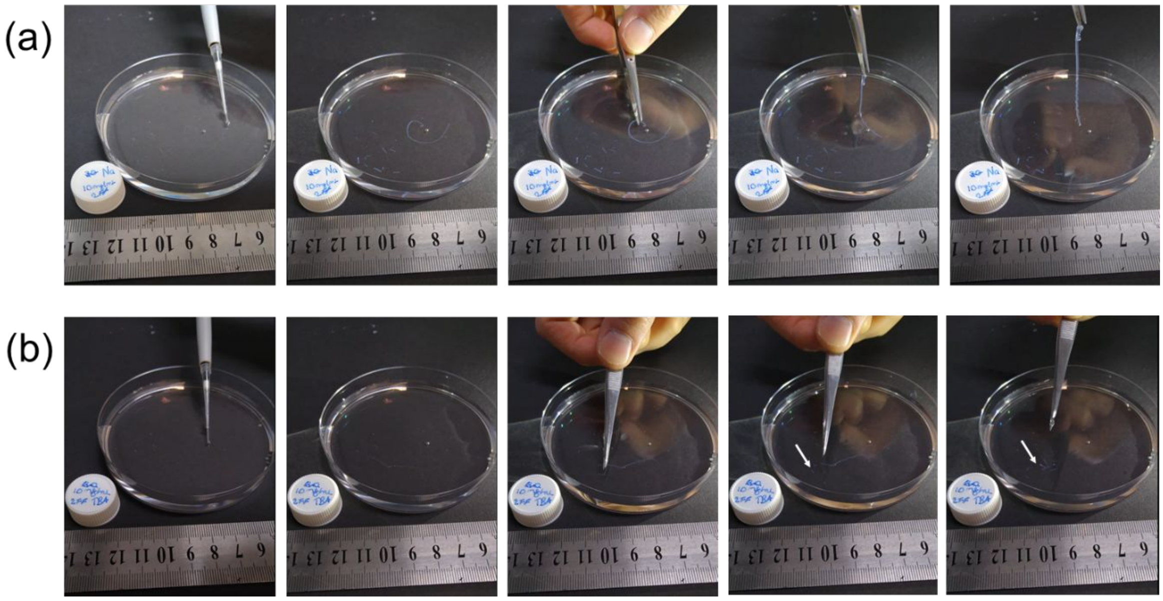

Figure S49. Images taken from videos showing the preparation and mechanical strength of gel noodles formed with 10 mg/mL 2NapFF with either (a) NaOH or (b) TBAOH. The 'NaOH' noodle can be readily lifted out of the trigger bath with tweezers whereas the 'TBAOH' breaks upon attempts at lifting. White arrows have been added to guide the eye to the broken pieces of gel noodle. 50 mM $CaCl_2$ was used as a trigger bath and a ruler added for scale.

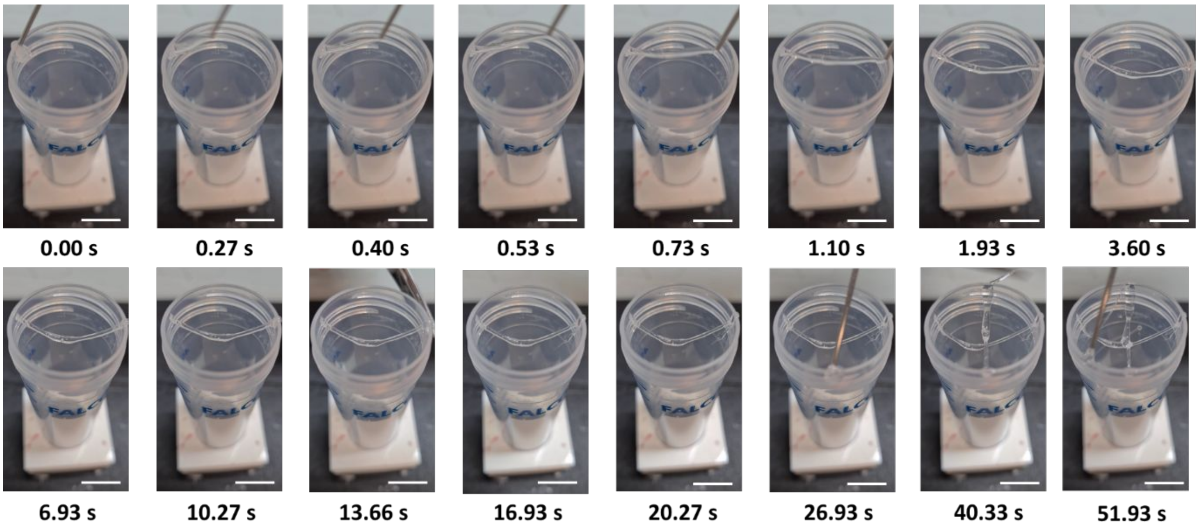

Figure S50. Frames (with time stamps) from a video showing the stringing of 75 mg/mL 2NapFF with TBAOH across a Falcon tube. Scale bars represent 1 cm.

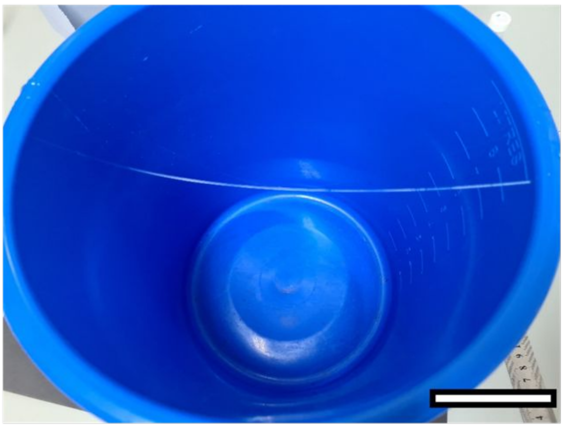

Figure S51. Stringing 2NapFF-TBA 75 mg/mL over larger surfaces. Scale bar represents 10 cm.

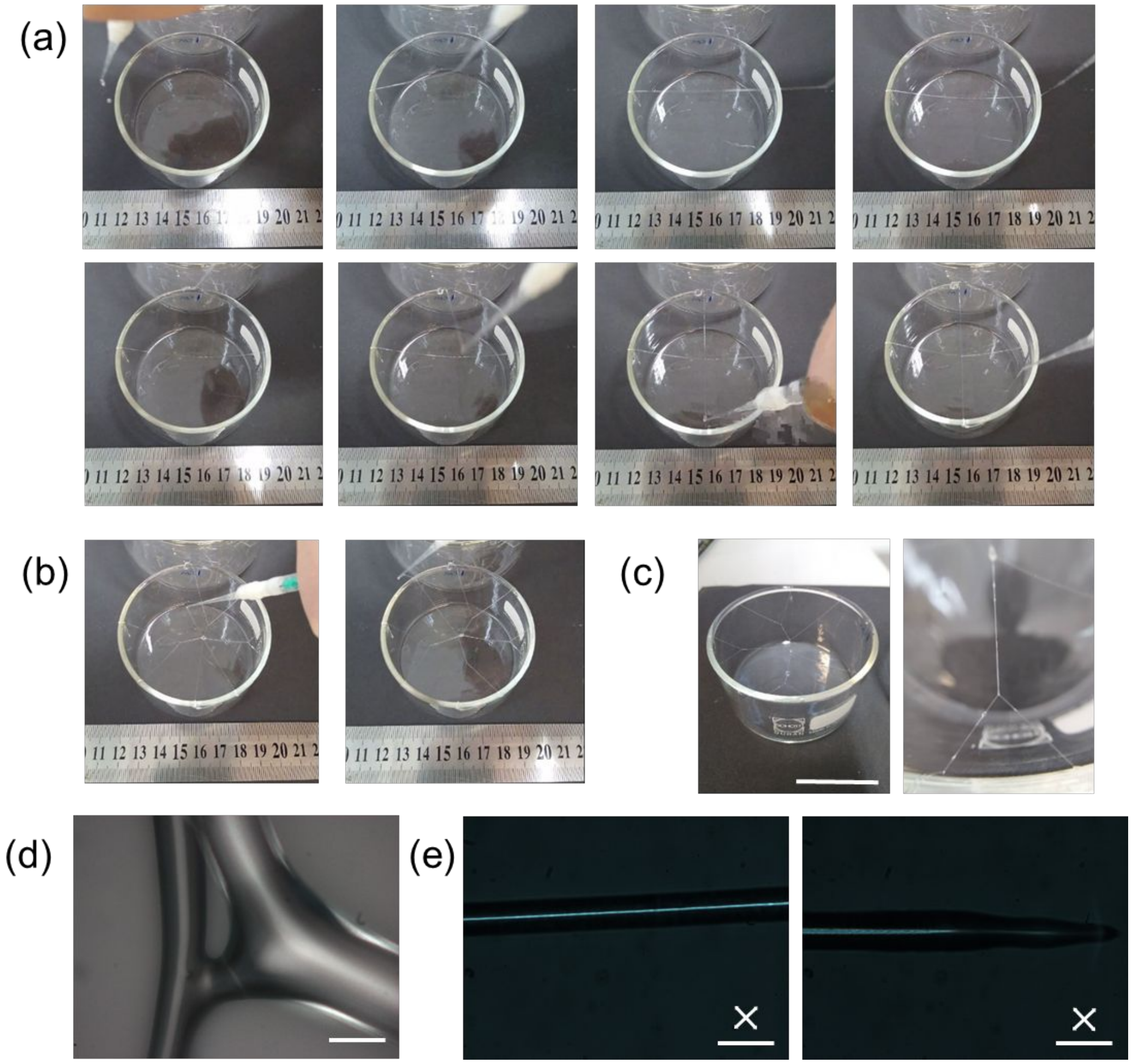

Figure S52. (a) Stills from a video showing how the liquid filaments can be bridged across an 8 cm gap and then connected to form a cross. (b) Stills from the video showing how this process can be repeated to build a complex web-like structure. (c) Photographs of the 'web' after being air dried overnight. Scale bar represents 3.5 mm. (d) Reflectance optical microscopy of the web and (e) cross polarised optical microscopy of the web. Optical microscope images at 20x magnification. Microscope scale bars represent 0.1 mm. White cross represents polariser direction.

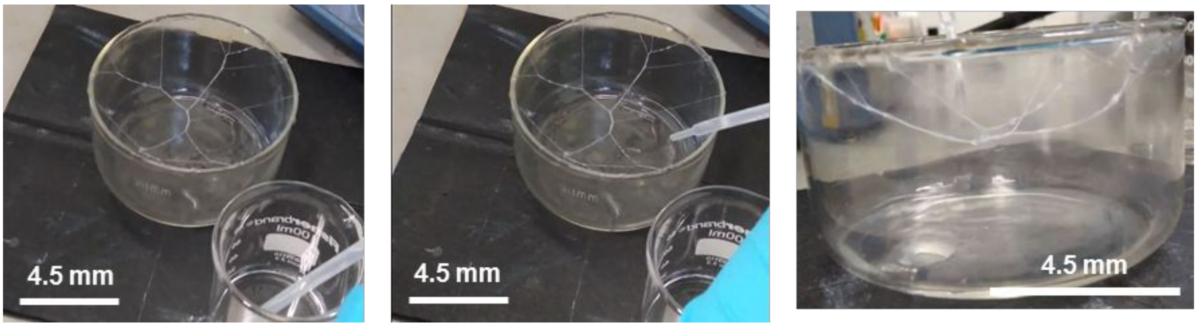

Figure S53. (a) Stills from a video showing the addition of 12 M HCl to the dish to create acidic vapour that gels the filaments of a wet 'web'. The opacity of the web increases as it is gelled.

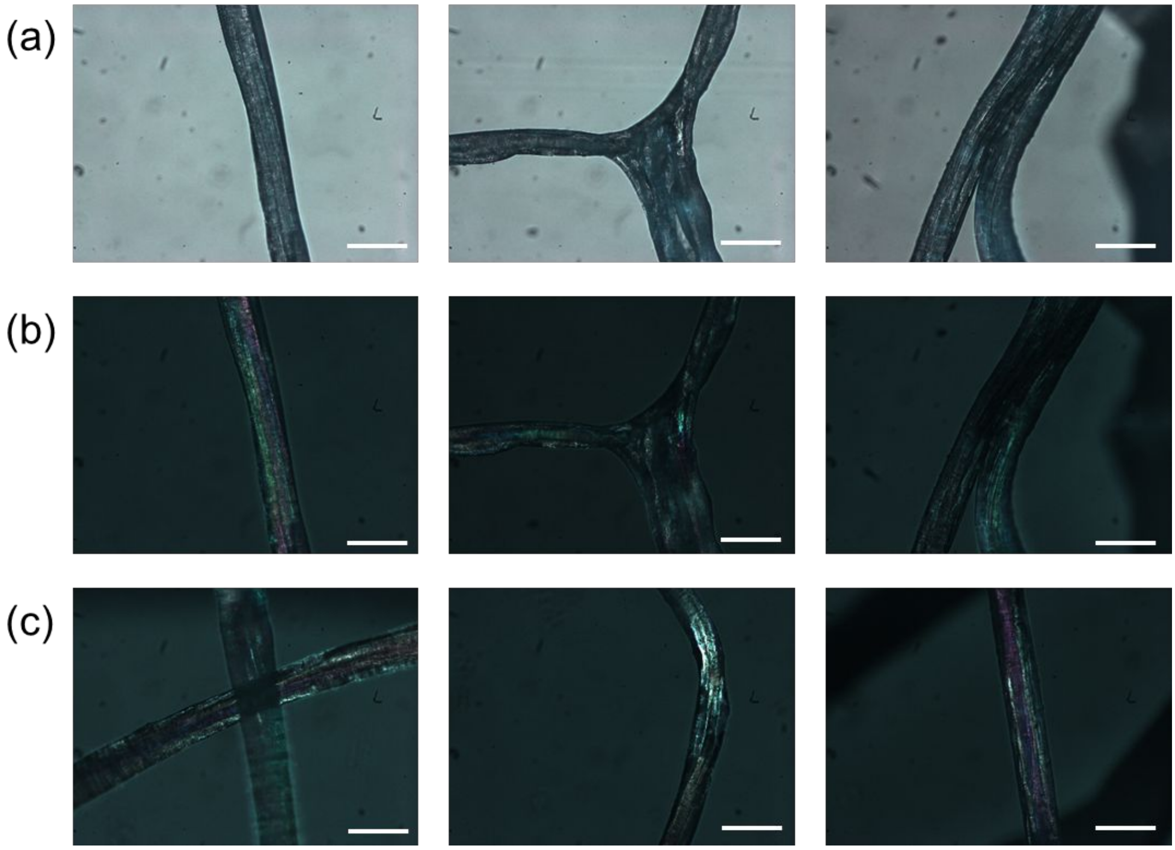

Figure S54. Optical microscope images of the acidified spider web at 10x magnification. Top two rows show (a) normal light and (b) cross polarised light images. (c) Additional cross polarised microscopy images. Scale bars represent 0.2 mm.

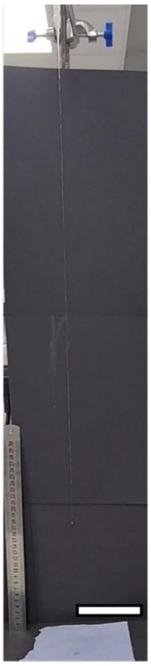

Figure S55. Long strings (77 cm) can be formed from 2NapFF-TBA when allowed to fall freely with gravity. Scale bars represent 10 cm.

Data for 1ThNapFF

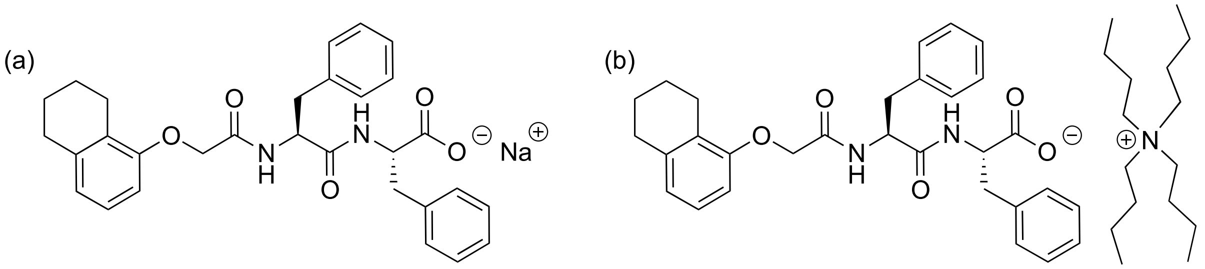

Figure S56. Chemical structures of (a) 1ThNapFF-Na and (b) 1ThNapFF-TBA.

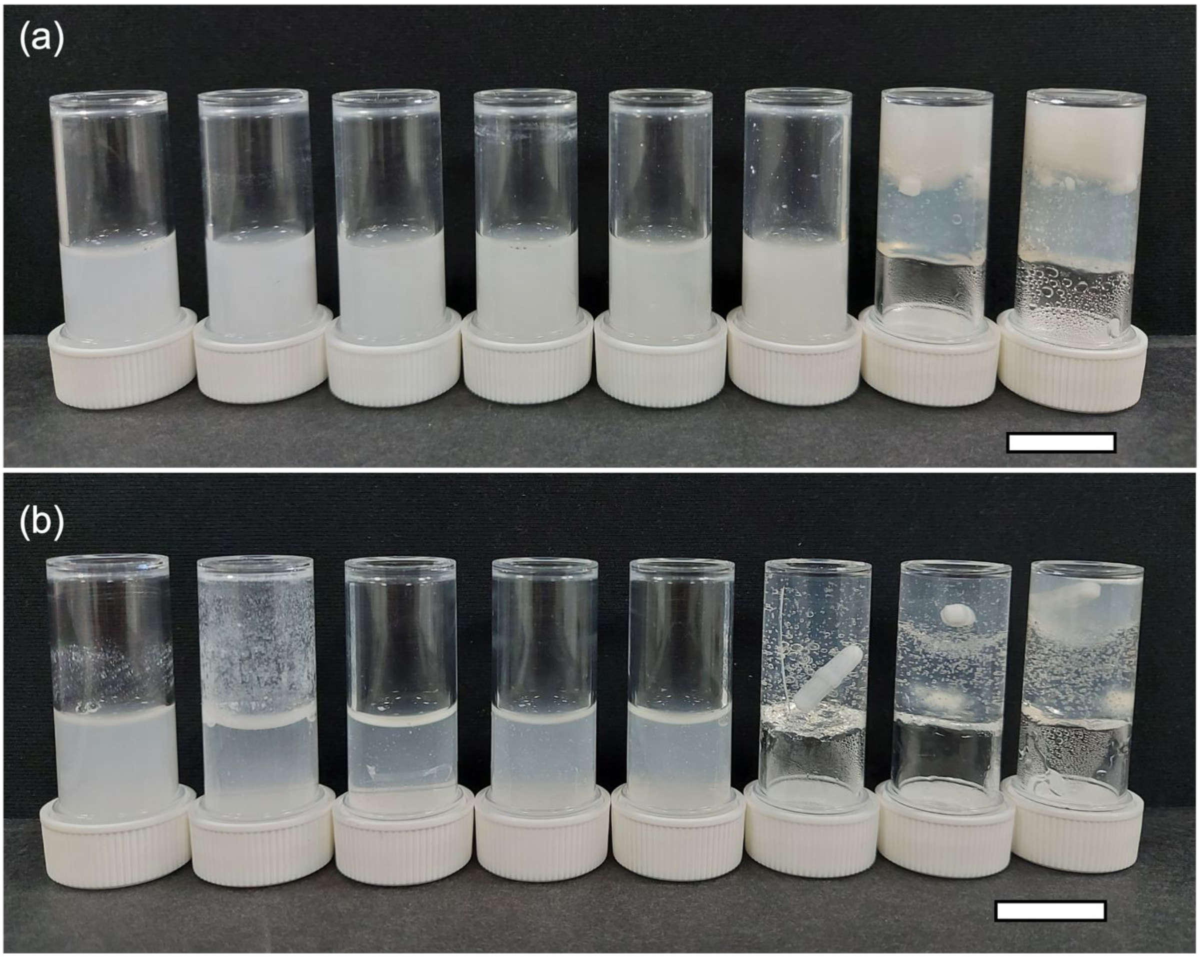

Figure S57. (a) Photographs of 1ThNapFF-Na prepared at different concentrations (left to right): 2 mg/mL, 5 mg/mL, 10 mg/mL, 15 mg/mL, 20 mg/mL, 30 mg/mL, 40 mg/mL and 50 mg/mL. (b) Photographs of 1ThNapFF-TBA prepared at different concentrations (left to right): 2 mg/mL, 4 mg/mL, 6 mg/mL, 8 mg/mL, 10 mg/mL, 15 mg/mL, 20 mg/mL and 25 mg/mL. For (a) and (b), the scale bar represents 2 cm.

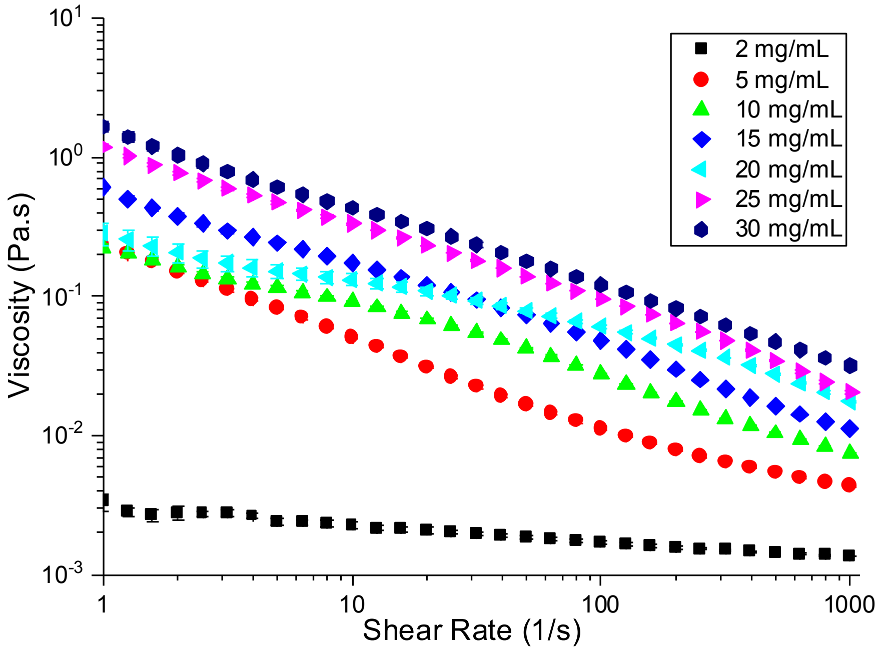

Figure S58. Viscosity data for 1ThNapFF-Na at different concentrations of gelator. Error bars represent the standard deviation between measurements, which were carried out in triplicate.

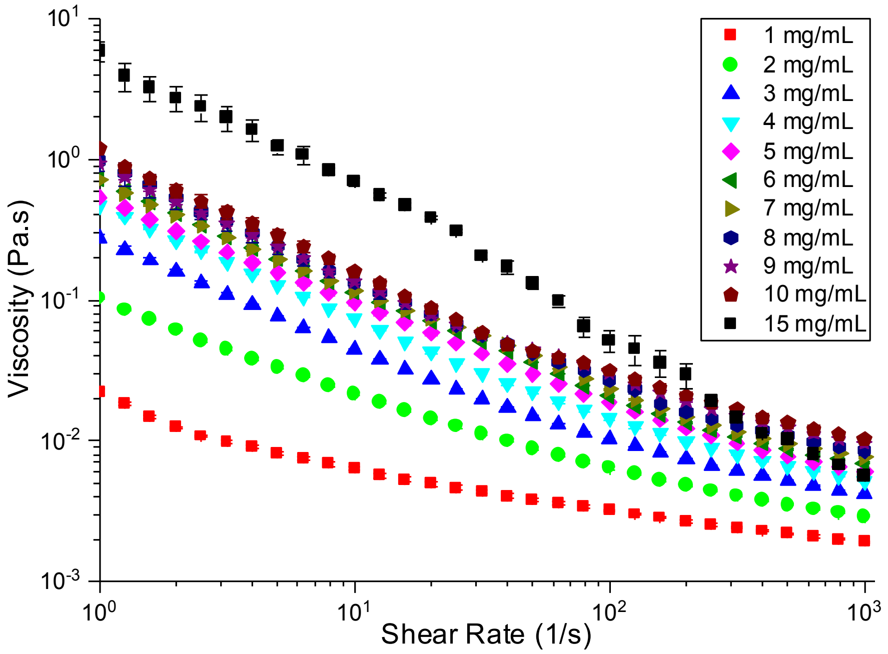

Figure S59. Viscosity data for 1ThNapFF-TBA at different concentrations of gelator. Error bars represent the standard deviation between measurements, which were carried out in triplicate.

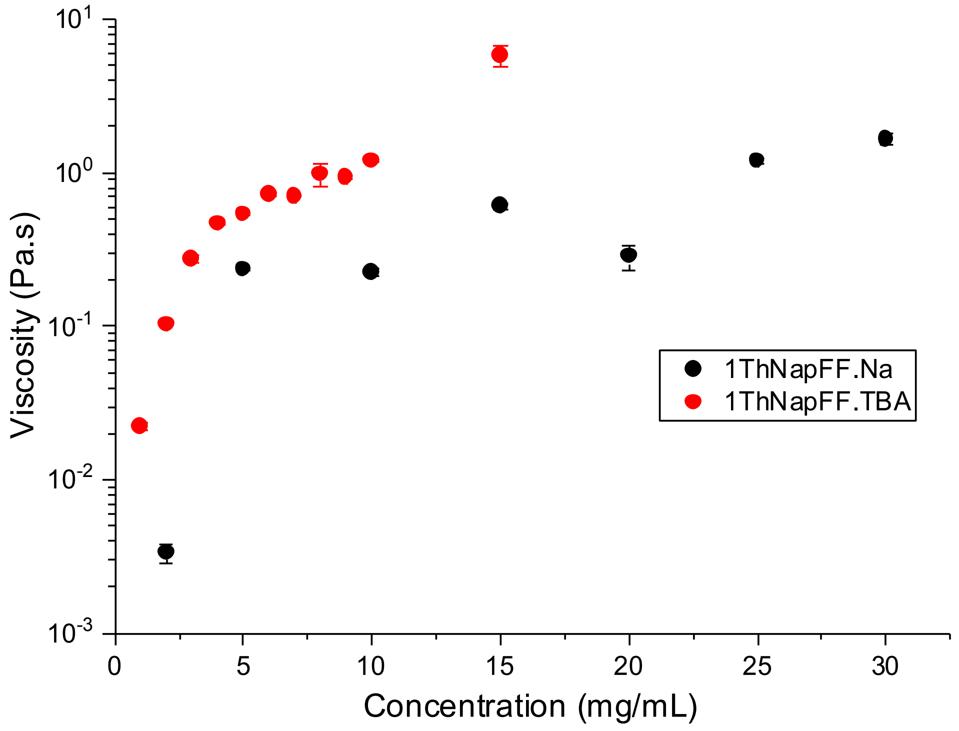

Figure S60. Comparison of viscosity at a shear rate of 1 s^{-1} for 1ThNapFF-Na (black) and 1ThNapFF-TBA (red).

Table S4. SAXS fitting parameters for increasing concentrations of 1ThNapFF-Na

	**5 mg/mL**	**10 mg/mL**	**15 mg/mL**	**20 mg/mL**	**25 mg/mL**	**30 mg/mL**
Scale	2.4 ± 0.02	6.2 ± 0.06	16.6 ± 0.1	14.2 ± 0.1	26.6 ± 0.1	40.1 ± 0.1
Background	0.009 (fixed)	0.02 ± 0.0008	0.02 ± 0.0008	0.01 ± 0.0009	0.03 ± 0.0009	0.03 ± 0.0009
Length / Å	>1000	>1000	>1000	>1000	>1000	>1000
Kuhn Length / Å	266.3 ± 7.9	123.1 ± 4.3	>1000	>1000	>1000	>1000
Radius / Å	32.8 ± 0.2	22.6 ± 0.2	22.9 ± 0.1	22.7 ± 0.1	22.5 ± 0.1	22.6 ± 0.03
Chi^2	1.60	1.09	1.16	1.15	1.63	1.46

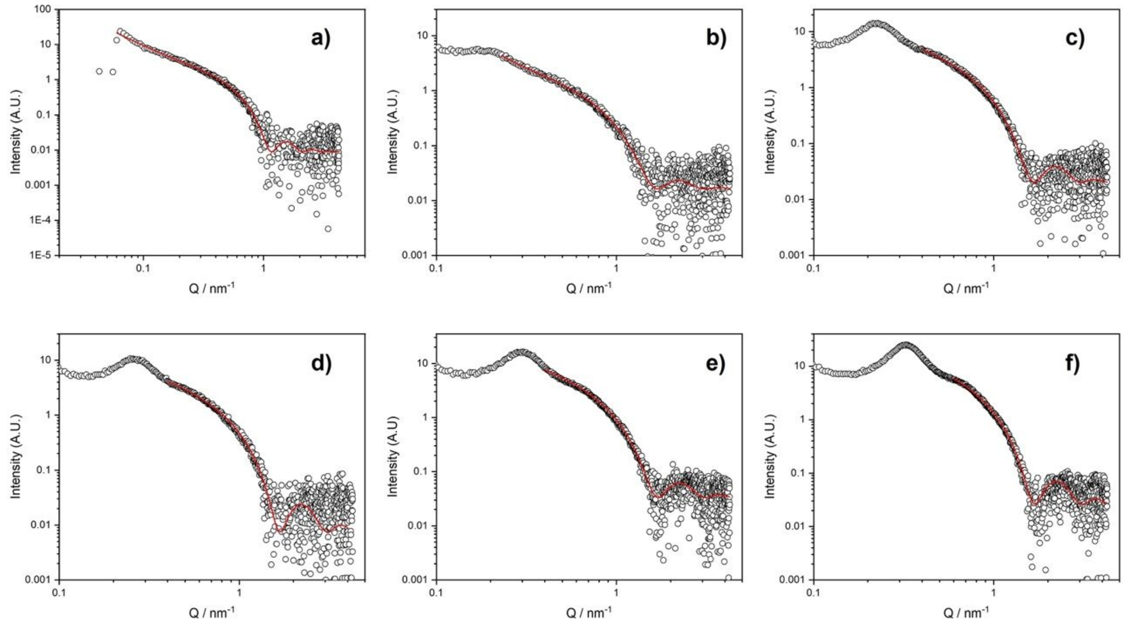

Figure S61. SAXS data (black circles) and form factor fits (red line) to 1ThNapFF-Na; a) 5 mg/mL; b) 10 mg/mL; c) 15 mg/mL; d) 20 mg/mL; e) 25 mg/mL; f) 30 mg/mL.

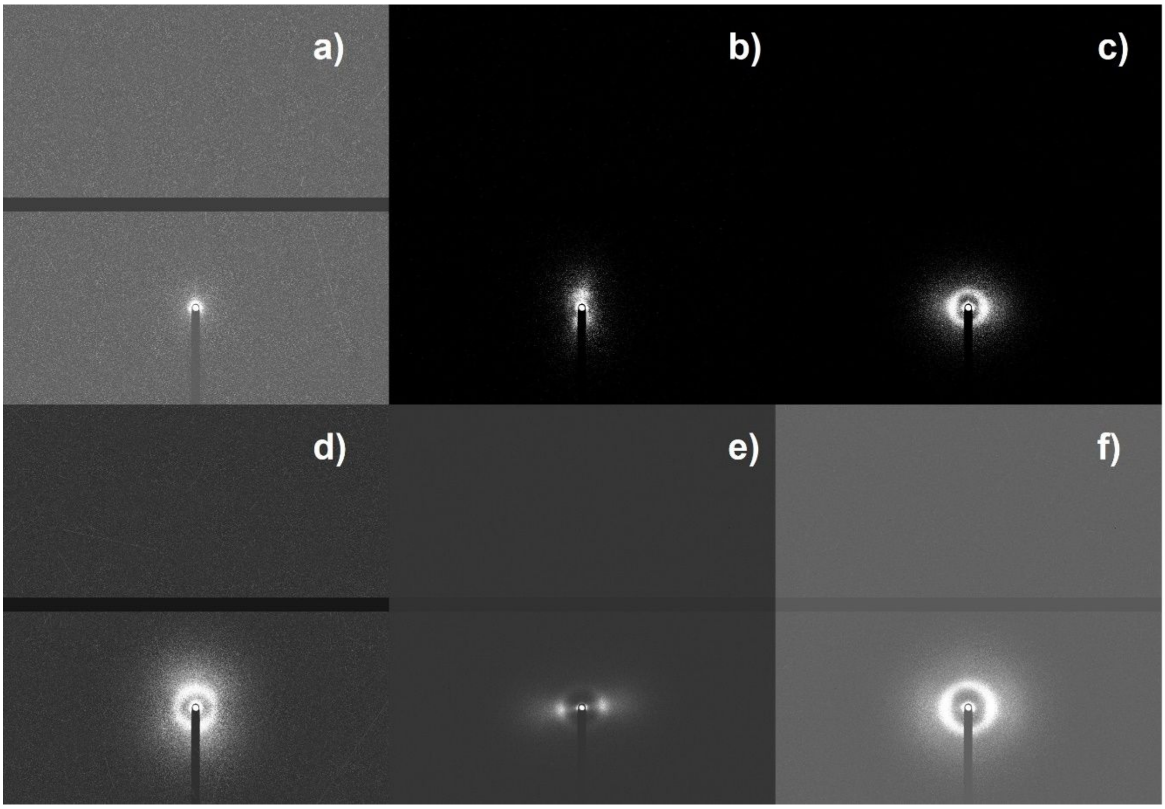

Figure S62. 2D SAXS data (black circles) and form factor fits (red line) to 1ThNapFF-Na; a) 5 mg/mL; b) 10 mg/mL; c) 15 mg/mL; d) 20 mg/mL; e) 25 mg/mL; f) 30 mg/mL.

Table S5. SAXS fitting parameters for increasing concentrations of 1ThNapFF-TBA

	4 mg/mL	6 mg/mL	7 mg/mL	8 mg/mL	9 mg/mL	10 mg/mL	15 mg/mL
Scale	1.8 ± 0.039	2.8 ± 0.05	3.6 ± 0.05	7.4 ± 0.1	4.2 ± 0.05	7.4 ± 0.1	12.5 ± 0.1
Background	0.03 ± 0.0008	0.01 (fixed)	0.02 (fixed)	0.2 ± 0.001	0.02 (fixed)	0.1 ± 0.001	0.04 ± 0.0009
Length / Å	>1000	>1000	>1000	>1000	>1000	>1000	>1000
Kuhn length / Å	68.0 ± 4.5	430.4 ± 210.8	>1000	>1000	>1000	>1000	>1000
Radius / Å	25.0 ± 0.5	20.3 ± 0.2	18.7 ± 0.2	14.1 ± 0.2	19.3 ± 0.2	14.9 ± 0.1	16.9 ± 0.08
Chi^2	2.66	1.27	1.37	1.17	2.36	1.19	1.13

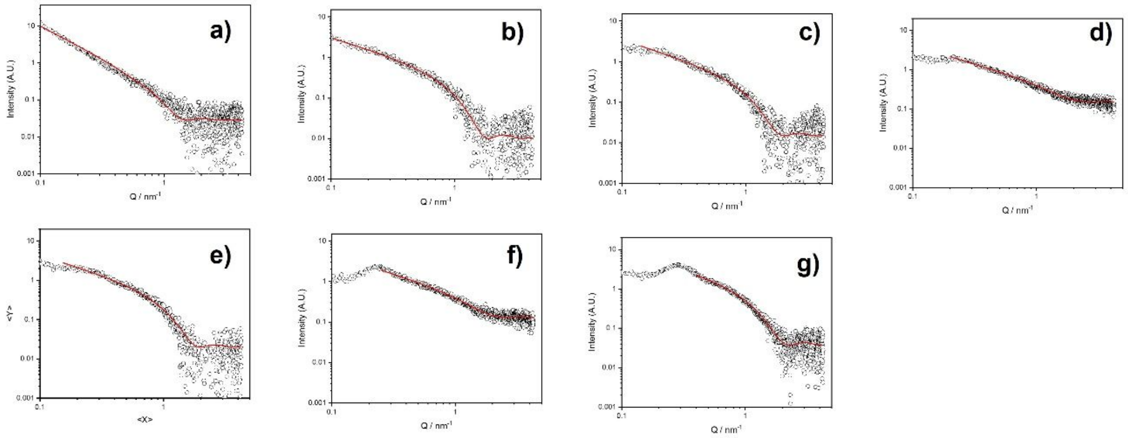

Figure S63. SAXS data (black circles) and form factor fits (red line) to 1ThNapFF-TBA; a) 4 mg/mL; b) 6 mg/mL; c) 7 mg/mL; d) 8 mg/mL; e) 9 mg/mL; f) 10 mg/mL; g) 15 mg/mL.

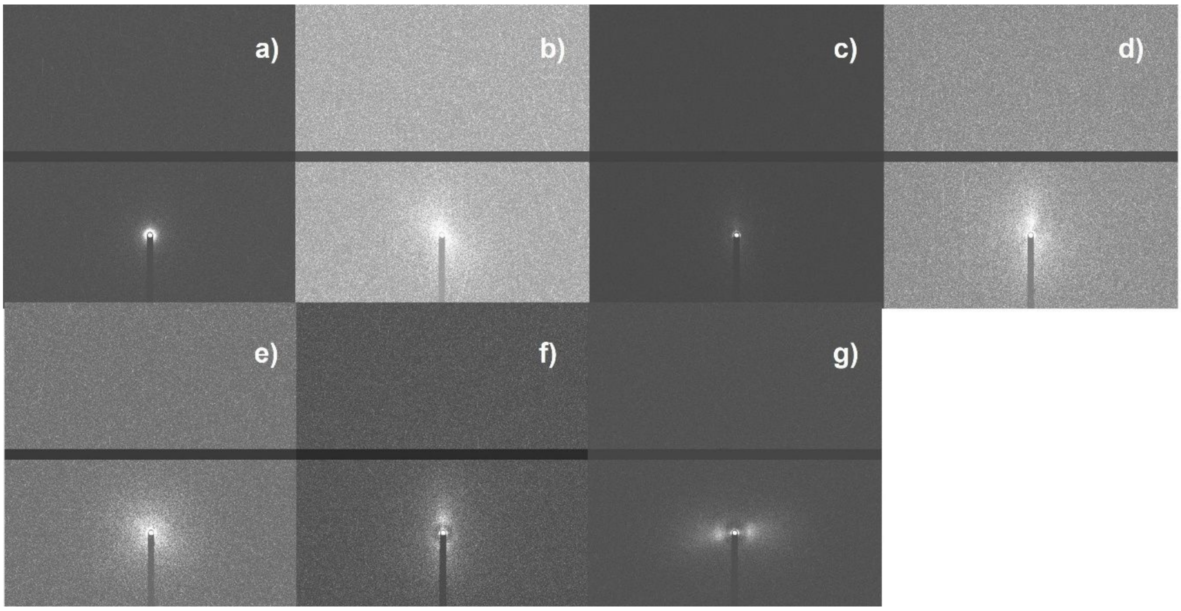

Figure S64. 2D SAXS data to 1ThNapFF-TBA a) 4 mg/mL; b) 6 mg/mL; c) 7 mg/mL; d) 8 mg/mL; e) 9 mg/mL; f) 10 mg/mL; g) 15 mg/mL.

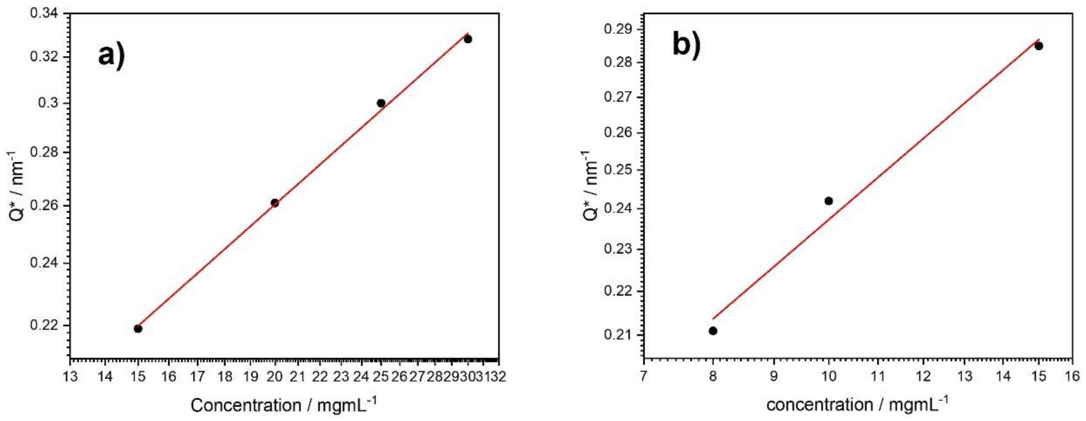

Figure S65. Concentration v Q* plots for a) 1ThNapFF-Na (gradient 0.59); b) 1ThNapFF-TBA (gradient 0.47)

References

1. C. A. Dreiss, *Soft Matter*, 2007, **3**, 956-970.
2. J. Yang, *Curr. Op. Coll. Int. Sci.*, 2002, **7**, 276-281.
3. S. A. Rogers, M. A. Calabrese and N. J. Wagner, *Curr. Op. Coll. Int. Sci.* 2014, **19**, 530-535.
4. X. Wu, Y. Wu, S. Yang, M. Zhao, M. Gao, H. Li and C. Dai, *Soft Matter*, 2016, **12**, 4549-4556.
5. K. Sakai, K. Nomura, R. G. Shrestha, T. Endo, K. Sakamoto, H. Sakai and M. Abe, *Langmuir*, 2012, **28**, 17617-17622.
6. T. Shikata, H. Hirata and T. Kotaka, *Langmuir*, 1988, **4**, 354-359.
7. J. Narayanan, C. Manohar, F. Kern, F. Lequeux and S. J. Candau, *Langmuir*, 1997, **13**, 5235-5243.
8. P. A. Hassan, S. J. Candau, F. Kern and C. Manohar, *Langmuir*, 1998, **14**, 6025-6029.
9. N. R. Agrawal, X. Yue, Y. Feng and S. R. Raghavan, *Langmuir*, 2019, **35**, 12782-12791.
10. E. R. Draper, H. Su, C. Brasnett, R. J. Poole, S. Rogers, H. Cui, A. Seddon and D. J. Adams, *Angew. Chem. Int. Ed.*, 2017, **56**, 10467-10470.
11. J. Fan, F. Liu and Z. Wang, *Int. J. Pharm.*, 2016, **497**, 248-254.
12. J. M. Dealy and T. K. P. Vu, *J. Non-Newton Fluid*, 1977, **3**, 127-140.
13. R. A. Stratton and A. F. Butcher, *J. Polym. Sci. Pol. Phys.*, 1973, **11**, 1747-1758.
14. J.-F. Berret, in *Molecular Gels: Materials with Self-Assembled Fibrillar Networks*, eds. R. G. Weiss and P. Terech, Springer Netherlands, Dordrecht, 1st edn., 2006, pp. 667-720.
15. K. McAulay, B. Dietrich, H. Su, M. T. Scott, S. Rogers, Y. K. Al-Hilaly, H. Cui, L. C. Serpell, Annela M. Seddon, E. R. Draper and D. J. Adams, *Chem. Sci.*, 2019, **10**, 7801-7806.
16. J. M. Buffa, U. Casado, V. Mucci and M. I. Aranguren, *Cellulose*, 2019, **26**, 2317-2332.
17. M. T. Sims, L. C. Abbott, R. M. Richardson, J. W. Goodby and J. N. Moore, *Liquid Crystals*, 2019, **46**, 11-24.
